# Ovicidal and Insecticidal Activities of Pyriproxyfen Derivatives with an Oxime Ester Group

**DOI:** 10.3390/molecules22060958

**Published:** 2017-06-08

**Authors:** Guo-Shao Sun, Xin Xu, Shu-Hui Jin, Le Lin, Jian-Jun Zhang

**Affiliations:** Department of Applied Chemistry, College of Science, China Agricultural University, Beijing 100193, China; guoshaosun@163.com (G.-S.S.); lijinming0711@cau.edu.cn (X.X.); linlel@163.com (L.L.)

**Keywords:** pyriproxyfen metabolite, oxime ester derivatives, insecticidal activity, ovicidal activity

## Abstract

Based on the structural framework of a pyriproxyfen metabolite, nineteen oxime ester derivatives were synthesized via reaction of the carboxylic acids with 4-(2-(2-pyridinyloxy)ethoxy)benzaldehyde oxime. The corresponding structures were comprehensively characterized by ^1^H-nuclear magnetic resonance (NMR), ^13^C-NMR, and electrospray ionization high-resolution mass spectrometry (ESI-HRMS). All of the compounds were screened for their insecticidal activities against *Plutella xylostella* and *Myzus persicae*, and for their ovicidal activities against *Helicoverpa armigera* eggs. The results obtained show that most of the oxime ester derivatives displayed moderate to high insecticidal activities and ovicidal activities at a concentration of 600 μg/mL. In particular, the ovicidal activity of compounds **5j**, **5o**, **5p**, **5q**, and **5s** was determined to be 100%. Importantly, some of the compounds presented even higher biological activities than the reference compound pyriproxyfen. For example, compound **5j** displayed an insecticidal activity value of 87.5% against *Myzus persicae*, whereas the activity value of pyriproxyfen was 68.3% at a concentration of 600 μg/mL. Among the synthesized compounds **5j** and **5s** exhibited broad biological activity spectra.

## 1. Introduction

**Figure 1:**
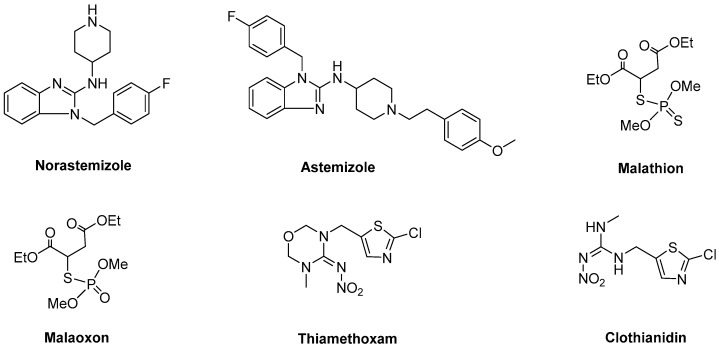
Chemical structures of malathion, malaoxon, thiamethoxam, clothianidin, astemizole, and norastemizole.

In the 1930s, various research groups reported that prontosil exhibits a suitable therapeutic effect in mice infected with different bacterial strands. However, the results were deemed abnormal with respect to the antibacterial activity of prontosil being absent in vitro [1]. The metabolite of prontosil, sulfanilamide, exhibited a strong antibacterial activity in vivo, indicating that the antibacterial activity of prontosil could mainly be attributed to this metabolite [1,2]. Since then, dozens of sulfonamide species have been developed and used for the treatment of bacterial diseases. Furthermore, the use of drug metabolites as lead compounds has become one of the new drug development approaches widely used in the pharmaceutical industry. For example, norastemizole (Figure 1), stemming from astemizole (Figure 1), was found to increase selectivity in the process of targeting receptors [3].

Concurrently, pesticides also generate a series of pesticide metabolites in organisms through a variety of metabolic pathways. In some cases, these pesticide metabolites may exhibit a good biological activity. For example, malaoxon (Figure 1) represents the metabolite of malathion (Figure 1). However, the inhibitory effect of malaoxon to acetylcholinesterase was found to be increased compared to malathion [4]. A more common example is thiamethoxam (Figure 1) that demonstrates a poor insecticidal activity compared to clothianidin (Figure 1), which is generated by degradation of thiamethoxam [5,6]. In our previous studies, a series of diamide derivatives were designed and synthesized based on a benalaxyl metabolite and the target compounds showed excellent fungicidal activities against *Phytophthora capsici* and *Rhizoctonia solani* [7]. From these findings, it can be hypothesized that the chemical structure of the pesticide metabolites may also provide critical information for the future development of pesticide lead compounds.

**Figure 2:**
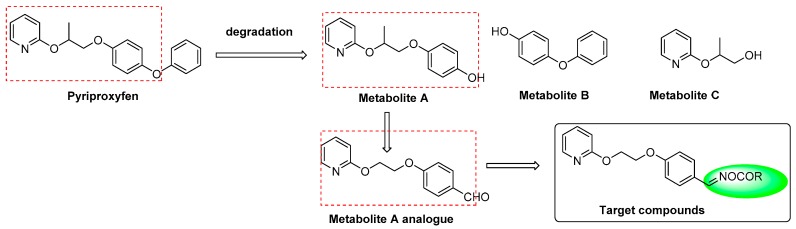
Molecular structure of the target compounds.

Pyriproxyfen, a juvenile hormone analogue, proves to be highly selective for target organisms with lower mammalian toxicity, and has been clinically used to control the spread of a wide range of arthropods [8,9]. 4-(2-(pyridin-2-yloxy)propoxy)-phenol (metabolite A, Figure 2), 4-phenoxyphenol (metabolite B, Figure 2), and 3-(pyridin-2-yloxy)butan-1-ol (metabolite C, Figure 2) were generated from degradation of pyriproxyfen ex vivo, as well as in animals and plants in vivo [10,11,12,13]. Metabolite A was synthesized and demonstrated a moderate insecticidal activity as well as ovicidal activity. 4-(2-(2-pyridinyloxy)ethoxy)benzaldehyde, a metabolite A analogue, retained the structural skeleton of pyriproxyfen but exhibited potent ovicidal activities against *Plutella xylostella*, *Myzus persicae*, and *Helicoverpa armigera* eggs, with overall activity values of 40.2%, 32.5%, and 75.2%, respectively. Therefore, we hypothesize that 4-(2-(2-pyridinyloxy)ethoxy) benzaldehyde may be used as a lead compound for further studies.

**Scheme 1:**
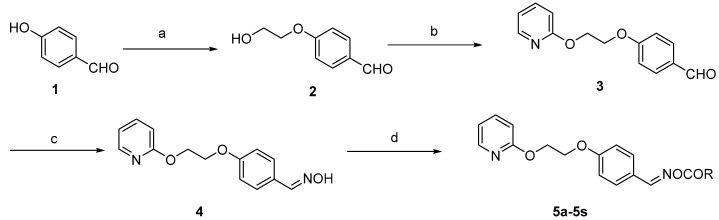
General synthetic route for target compounds. Reagents and conditions: (a) BrCH_2_CH_2_OH, K_2_CO_3_, dimethylformamide (DMF), 100 °C, 12 h; (b) i. NaH, DMF, 0–5 °C; ii. 2-fluoropyridine, DMF, 55 °C, 6 h; (c) NH_2_OH/NaOH, EtOH/H_2_O, r.t.; (**d**) substituted carboxylic acid, dicyclohexylcarbodiimide (DCC), 4-dimethylaminopyridine (DMAP), dichloromethane (DCM).

In 1963, tranid, the first oxime ester insecticide, was developed. Thereafter, oxime carbamate insecticides (e.g., methomyl) and oxime phosphate insecticides (e.g., phoxim) have been developed. These oxime ester compounds have attracted considerable attention in the scientific community due to their broad-spectrum biological activities as plant virucides [14], fungicides [15,16], insecticides [17,18,19], and herbicides [20]. In our group, oleanolic acid and avermectin oxime ester derivatives were synthesized and exhibited suitable biological activities, providing further incentive to study the oxime ester group in more detail [16,21]. Continuing this work, nineteen novel oxime ester derivatives were synthesized (cf. Scheme 1) and their ovicidal activities against *Plutella xylostella*, *Myzus persicae*, and *Helicoverpa armigera* eggs were evaluated.

## 2. Results and Discussion

### 2.1. Chemistry

As shown in Scheme 1, nineteen oxime ester derivatives were synthesized. Compound **2** was prepared by reacting commercially available 4-hydroxybenzaldehyde with 2-bromoethanol in *N*,*N*-dimethylformamide as previously reported in the literature [22]. A subsequent reaction with 2-fluoropyridine in the presence of sodium hydride led to the formation of intermediate **3** [23]. Upon addition of sodium hydride, the mixture was placed in an ice bath due to the release of a large amount of heat as the reaction progressed. Next, intermediate **3** was directly converted to the key intermediate, **4**, by treatment with hydroxylamine hydrochloride [24]. Finally, the corresponding target oxime esters (**5a**–**5s**) were obtained via intermediate **4** and reactions with carboxylic acid functionalities in the presence of dicyclohexylcarbodiimide (DCC) and 4-dimethylaminopyridine (DMAP). The physical data of the target compounds **5a**–**5s** are given in Table 1. ^1^H- and ^13^C-NMR spectra of all compounds are presented in the Supplementary Materials.

Since the oxime group can exist both in the E- or Z-configuration, it was necessary to determine the geometries of all the oxime target compounds **5a**–**5s**. Attempts to obtain X-ray quality single crystals of the oxime intermediates or target products proved unsuccessful in this study. However, the oxime geometry was considered to be in the E-configuration, an assumption that is in agreement with published data [24].

### 2.2. Biological Activities

#### 2.2.1. Insecticidal Activities

As highlighted in Table 2, the insecticidal activities of all target compounds against *Myzus persicae* and *Plutella xyllostella* were evaluated. With the substituent being a phenyl group, the results indicated that compounds **5a**–**5i** possessed 3.6% to 15.7% insecticidal activities against *Myzus persicae* at a concentration of 600 μg/mL, demonstrating that the substituent species and the position of the phenyl ring had no significant effect on the insecticidal activity. Compounds **5l**, 5**m**, and 5**s** showed 49.4%, 58.1%, and 25.9% insecticidal activities against *Myzus persicae* at a concentration of 600 μg/mL. As the substituent was changed to a pyridine group, compounds **5l** and **5m**, but not compound **5r**, exhibited improved insecticidal activities compared to compounds **5a**–**5i**. Compounds **5o** and **5p**, bearing an alkyl substituent, displayed moderate activity against *Myzus persicae*. Some of the synthesized compounds exhibited higher insecticidal activities than the reference compound pyriproxyfen. For instance, the activity rates of compounds **5j**, **5q** and **5s** against *Myzus persicae* were 87.5%, 73.6%, and 72.8% at 600 μg/mL, respectively, whereas pyriproxyfen featured a value no higher than 70% at the same concentration. Moreover, some of the compounds exhibited moderate insecticidal activities against *Myzus persicae* upon reduction of the concentration to 200 μg/mL; for example, compounds **5j**, **5q**, and **5s** showed insecticidal activities of 53.8%, 36.4%, and 43.1%, respectively, further suggesting that the futyl, thienyl and 2,2,3,3-Me-cyclopropyl groups exhibit a great influence on the activities. Many of the synthesized compounds exhibited no to moderate insecticidal activities against *Plutella xyllostella* at a concentration of 600 μg/mL. Compounds **5b**, **5e**, **5f**, **5g**, **5l**, **5n**, **5o**, and **5p** showed no or very weak insecticidal activities against *Plutella xyllostella*. The activity rates of compounds **5j** and **5s** were 68.7% and 57.1%, respectively. Considering *Plutella xyllostella*, any structure–activity relationships were not obvious.

#### 2.2.2. Ovicidal Activities

Besides insecticidal activities, the ovicidal activities of the new compounds against *Helicoverpa armigera* eggs were also explored. As shown in Table 2, most of the target compounds exhibited moderate to excellent ovicidal activities against *Helicoverpa armigera* eggs at a concentration of 600 μg/mL. Compounds **5a**, **5b**, **5c**, **5d**, **5f**, and **5m** showed limited ovicidal activities at 600 μg/mL, with activity values of less than 30%. Compounds **5j**, **5o**, **5p**, **5q**, and **5s** all featured 100% activity at 600 μg/mL. At a concentration of 200 μg/mL, compounds **5o**, **5p**, and **5s** still exhibited ovicidal activities of 67.8%, 79.5%, and 64.3% against *Helicoverpa armigera* eggs, values that prove to be higher than that of pyriproxyfen (i.e., 54.8%). Compound **5j** featured an ovicidal activity value of 45.3% against *Helicoverpa armigera* eggs. Similar to that of insecticidal activities against *Myzus persicae*, compounds **5j**, **5q**, and **5s** exhibited more potent ovicidal activities against *Helicoverpa armigera* eggs. However, compounds **5o** and **5p** with moderate insecticidal activities against *Myzus persicae*, showed the best ovicidal activities against *Helicoverpa armigera* eggs. The latter finding may indicate that the presence of alkyl groups could improve the overall ovicidal activity.

## 3. Materials and Methods

### 3.1. Chemistry

#### 3.1.1. General Information

All reagents used were of analytical grade and the solvents were dried according to standard procedures. ^1^H-NMR and ^13^C-NMR spectra were recorded on a Bruker Avance (Bruker Co., Karlsruhe, Germany) 300 spectrometer (300 MHz, ^1^H; 75 MHz, ^13^C) in CDCl_3_ as a solvent and with tetramethylsilane as the internal standard. High-resolution mass spectra (ESI-HRMS) were recorded on a Bruker Daltonics Bio-TOF-Q III mass spectrometer (Bruker Co., Karlsruhe, Germany). The melting points were measured on a YRT-3 apparatus and the values are shown in the uncorrected form. Column chromatographic purification was carried out using 200–300 mesh silica gel. The reactions were monitored by analytical thin-layer chromatography (TLC) carried out on silica gel GF/UV 254 TLC plates.

#### 3.1.2. Synthesis of 4-(2-Hydroxyethoxy)benzaldehyde (**2**)

To a solution of 4-hydroxybenzaldehyde (10.0 g, 82.0 mmol) in dry DMF (80 mL), K_2_CO_3_ (22.6 g, 163.8 mmol) and 2-bromoethanol (12.3 g, 98.3 mmol) were added. The reaction mixture was heated to 100 °C for 12 h with monitoring by TLC. The resulting solution was extracted with ethyl acetate (3 × 80 mL) and dried over anhydrous Na_2_SO_4_. The solvents of the combined organic layer were removed under reduced pressure and the residue was purified by flash chromatography on silica gel (200–300 mesh) using petroleum ether and ethyl acetate (*v/v* = 8:1) as eluents to afford compound **2** as a pale yellow liquid (11.3 g, 83%). ^1^H-NMR (300 MHz, CDCl_3_) δ 9.86 (s, 1H, PhCHO), 7.74–7.90 (m, 2H, Ar-H), 6.92–7.10 (m, 2H Ar-H), 4.13–4.20 (m, 2H, PhOCH_2_), 3.95–4.04 (m, 2H, C H_2_OH).

#### 3.1.3. Synthesis of 4-(2-(2-Pyridinyloxy)ethoxy)benzaldehyde (**3**)

To an ice-cooled solution of compound **2** (5.0 g, 30.1 mmol) in DMF (40 mL), 60% sodium hydride (1.4 g, 36.1 mmol) was slowly added within 10 min. After stirring for an additional 30 min, 2-fluoropyridine (3.5 g, 36.1 mmol) was added. The resulting mixture was then heated to 55 °C and allowed to react for another 6 h. The reaction was quenched by addition of ice water and the resulting crude product was extracted with ethyl acetate (3 × 50 mL) and dried over anhydrous Na_2_SO_4_. The combined organic layers were concentrated in vacuo and then purified by flash chromatography on silica gel (200–300 mesh) using petroleum ether and ethyl acetate (*v/v* = 10:1) as eluent to provide compound **3** as a white solid (6.4 g, 88%). ^1^H-NMR (300 MHz, CDCl_3_): δ 9.86 (s, 1H, PhCHO), 8.11–8.17 (m, 1H, Ar-H), 7.78–7.86 (m, 2H, Ar-H), 7.52–7.60 (m, 1H, Ar-H), 6.99–7.08 (m, 2H Ar-H), 6.84–6.92 (m, 1H Ar-H), 6.74–6.80 (m, 1H Ar-H), 4.58–4.78 (m, 2H, PyOCH_2_), 4.31–4.45 (m, 2H, PhOCH_2_).

#### 3.1.4. Synthesis of 4-(2-(2-Pyridinyloxy)ethoxy)benzaldehyde oxime (**4**)

Hydroxylamine hydrochloride (1.5 g, 26.1 mmol) was dissolved in water (10 mL) and neutralized with aqueous sodium hydroxide solution (10%). A solution of compound **3** (4.4 g, 18.1 mmol) in ethanol was slowly added to this mixture. The resulting suspension was stirred at room temperature with monitoring by TLC. After reaction completion, the solvent ethanol was removed in vacuo. The resulting solution was extracted with ethyl acetate (3 × 30 mL) and dried over anhydrous Na_2_SO_4_. The solvent of the combined organic layers was removed in vacuo to provide a residue which was then purified by silica gel (200–300 mesh) column chromatography using petroleum ether and ethyl acetate (*v/v* = 6:1) as eluents to afford compound **4** as a white solid (4.2 g, 90%). m.p.: 81–83 °C; ^1^H-NMR (300 MHz, CDCl_3_) δ 8.19 (dd, *J* = 5.0 Hz, 1.6 Hz, 1H, Ar-H), 8.12 (s, 1H, CH=N), 7.58–7.67 (m, 1H, Ar-H), 7.51–7.57 (m, 2H, Ar-H), 7.48 (s, 1H, NOH), 6.96–7.03 (m, 2H Ar-H), 6.89–6.96 (m, 1H Ar-H), 6.84 (d, *J* = 8.4 Hz, 1H, Ar-H), 4.65–4.84 (m, 2H, PyOCH_2_), 4.26–4.51 (m, 2H, PhOCH_2_). ^13^C-NMR (75 MHz, CDCl_3_): 163.18, 159.96, 149.44, 146.49, 138.79, 128.31, 125.03, 116.95, 114.78, 111.40, 66.43, 64.11; HRMS (ESI) calcd. for C_14_H_15_N_2_O_4_ (M + H)^+^ 275.1032, found 275.1031.

#### 3.1.5. General Procedure for the Synthesis of 4-(2-(2-Pyridinyloxy)ethoxy)benzaldehyde oxime ester derivatives (**5a**–**5s**)

A solution of the appropriate carboxylic acid (7.5 mmol) and DCC (7.5 mmol) was stirred in DCM (20 mL) in the presence of DMAP (100 mg). Compound **4** (5.8 mmol) was added to the reaction mixture upon stirring and stirring was continued for another 10 h with monitoring by TLC. The resulting liquid was collected by filtration and evaporated under reduced pressure. The resulting residue was purified by column chromatography on silica (200–300 mesh) using petroleum ether and ethyl acetate (*v/v* = 4:1~16:1) as eluents.

*4-(2-(2-Pyridinyloxy)ethoxy)benzaldehyde-O-benzoyl-oxime* (**5a**). ^1^H-NMR (300 MHz, CDCl_3_) δ 8.50 (s, 1H, CH=N), 8.24–8.01 (m, 3H, Ar-H), 7.84–7.68 (m, 2H, Ar-H), 7.65–7.54 (m, 2H, Ar-H), 7.49 (t, *J* = 7.5 Hz, 2H, Ar-H), 7.05–6.96 (m, 2H, Ar-H), 6.87–6.93 (m, 1H, Ar-H), 6.80 (d, *J* = 8.4 Hz, 1H, Ar-H), 4.76–4.66 (m, 2H, PyOCH_2_), 4.45–4.33 (m, 2H, PhOCH_2_); ^13^C-NMR (75 MHz, CDCl_3_) δ 163.31, 161.70, 156.30, 146.73, 138.73, 133.28, 130.23, 129.68, 128.85, 128.52, 122.83, 117.10, 115.08, 111.37, 66.69, 63.86; HRMS (ESI) calcd. for C_21_H_19_N_2_O_4_ (M + H)^+^ 363.1339, found 363.1341.

*4-(2-(2-Pyridinyloxy)ethoxy)benzaldehyde-O-(4-methoxybenzoyl)-oxime* (**5b**). ^1^H-NMR (300 MHz, CDCl_3_) δ 8.47 (s, 1H, CH=N), 8.16 (dd, *J* = 5.0 Hz, 1.5 Hz, 1H, Ar-H), 8.02–8.12 (m, 2H, Ar-H), 7.75 (d, *J* = 8.8 Hz, 2H, Ar-H), 7.66–7.52 (m, 1H, Ar-H), 7.07–6.87 (m, 5H, Ar-H), 6.80 (d, *J* = 8.4 Hz, 1H, Ar-H), 4.75–4.65 (m, 2H, PyOCH_2_), 4.43–4.30 (m, 2H, PhOCH_2_), 3.87 (s, 3H, OCH_3_); ^13^C-NMR (75 MHz, CDCl_3_) δ 163.84, 163.66, 163.30, 161.58, 155.92, 146.70, 138.71, 131.74, 130.14, 122.97, 121.03, 117.07, 115.03, 113.84, 111.34, 66.66, 63.86, 55.44; HRMS (ESI) calcd. for C_22_H_21_N_2_O_5_ (M + H)^+^ 393.1445, found 393.1444.

*4-(2-(2-Pyridinyloxy)ethoxy)benzaldehyde-O-(2-fluorobenzoyl)-oxime* (**5c**). 1H-NMR (300 MHz, CDCl_3_) δ 8.48 (s, 1H, CH=N), 8.22–8.011 (m, 1H, Ar-H), 8.07–7.95 (m, 1H, Ar-H), 7.80–7.67 (m, 2H, Ar-H), 7.66–7.51 (m, 2H, Ar-H), 7.26–7.14 (m, 2H, Ar-H), 7.07–6.95 (m, 2H, Ar-H), 6.86–6.92 (m, 1H, Ar-H), 6.80 (d, *J* = 8.4 Hz, 1H, Ar-H), 4.64–4.78(m, 2H, PyOCH_2_), 4.33–4.42 (m, 2H, PhOCH_2_); ^13^C-NMR (75 MHz, CDCl_3_) δ 163.29, 161.75, 156.69, 146.71, 138.71, 134.81, 132.30, 130.27, 124.18, 122.67, 117.13, 116.83, 115.07, 111.34, 66.67, 63.83; HRMS (ESI) calcd. for C_21_H_18_FN_2_O_4_ (M + H)^+^ 381.1245, found 381.1243.

*4-(2-(2-Pyridinyloxy)ethoxy)benzaldehyde-O-(2-(4-methoxyphenyl)acetyl)-oxime* (**5d**) ^1^H-NMR (300 MHz, CDCl_3_) δ 8.27 (s, 1H, CH=N), 8.20–8.09 (m, 1H, Ar-H), 7.72–7.61 (m, 2H, Ar-H), 7.61–7.51 (m, 1H, Ar-H), 7.30–7.23 (m, 2H, Ar-H), 7.02–6.93 (m, 2H, Ar-H), 6.92–6.85 (m, 3H, Ar-H), 6.78 (d, *J* = 8.4 Hz, 1H, Ar-H), 4.75–4.62 (m, 2H, PyOCH_2_), 4.45–4.26 (m, 2H, PhOCH_2_), 3.79 (s, 3H, OCH_3_), 3.73 (s, 2H, PhCH_2_CO); ^13^C-NMR (75 MHz, CDCl_3_) δ 169.27, 163.21, 161.54, 158.77, 155.74, 146.64, 138.66, 130.25, 130.02, 125.31, 122.63, 117.02, 114.95, 114.03, 111.25, 66.58, 63.77, 55.17, 39.05; HRMS (ESI) calcd. for C_23_H_23_N_2_O_5_ (M + H)^+^ 407.1601, found 407.1601.

*4-(2-(2-Pyridinyloxy)ethoxy)benzaldehyde-O-(2-(4-methylphenyl)acetyl)-oxime* (**5e**). ^1^H-NMR (300 MHz, CDCl_3_) δ 8.27 (s, 1H, CH=N), 8.15 (dd, *J* = 4.9 Hz, 1.6 Hz, 1H, Ar-H), 7.66 (d, *J* = 8.8 Hz, 2H, Ar-H), 7.54–7.62 (m, 1H, Ar-H), 7.30–7.18 (m, 2H, Ar-H), 7.15 (d, *J* = 7.9 Hz, 2H, Ar-H), 6.97 (d, *J* = 8.8 Hz, 2H, Ar-H), 6.86–6.94 (m, 1H, Ar-H), 6.79 (d, *J* = 8.3 Hz, 1H, Ar-H), 4.84–4.60 (m, 2H, PyOCH_2_), 4.44–4.28 (m, 2H, PhOCH_2_), 3.77 (s, 2H, PhCH_2_CO), 2.34 (s, 3H, PhCH_3_); ^13^C-NMR (75 MHz, CDCl_3_) δ 16919, 163.29, 161.61, 155.78, 146.72, 138.71, 136.88, 130.26, 130.10, 129.34, 129.13, 122.72, 117.08, 115.01, 111.34, 66.65, 63.83, 39.64, 21.06; HRMS (ESI) calcd. for C_23_H_23_N_2_O_4_ (M + H)^+^ 391.1652, found 391.1653.

*4-(2-(2-Pyridinyloxy)ethoxy)benzaldehyde-O-(4-ethoxybenzoyl)-oxime* (**5f**). ^1^H-NMR (300 MHz, CDCl_3_) δ 8.47 (s, 1H, CH=N), 8.16 (dd, *J* = 5.1 Hz, 1.3 Hz, 1H, Ar-H), 8.06–7.98 (m, 2H, Ar-H), 7.79–7.71 (m, 2H, Ar-H), 7.64–7.51 (m, 1H Ar-H), 7.06–6.84 (m, 5H, Ar-H), 6.80 (d, *J* = 8.3 Hz, 1H, Ar-H), 4.77–4.60 (m, 2H, PyOCH_2_), 4.33–4.42 (m, 2H, PhOCH_2_), 4.09 (q, *J* = 7.4 Hz, 2H, OCH_2_CH_3_), 1.45 (t, *J* = 7.4 Hz, 3H, OCH_2_CH_3_); ^13^C-NMR (75 MHz, CDCl_3_) δ 163.90, 163.32, 163.10, 161.59, 155.89, 146.72, 138.73, 131.75, 130.15, 117.08, 115.04, 114.27, 111.37, 66.68, 63.88, 63.76, 14.65; HRMS (ESI) calcd. for C_23_H_23_N_2_O_5_ (M + H)^+^ 407.1601, found 407.1604.

*4-(2-(2-Pyridinyloxy)ethoxy)benzaldehyde-O-(2-(2*,*4-dichlorobenzoyl))acetyl)-oxime* (**5g**). ^1^H-NMR (300 MHz, CDCl_3_) δ 8.27 (s, 1H, CH=N), 8.14–8.17 (m, 1H, Ar-H), 7.64–7.68 (m, 2H, Ar-H), 7.57–7.61 (m, 1H, Ar-H), 7.28–7.32 (m, 3H, Ar-H), 7.02–6.94 (m, 2H, Ar-H), 6.93–6.86 (m, 1H, Ar-H), 6.85–6.69 (m, 1H, Ar-H), 4.76–4.62 (m, 2H, PyOCH_2_), 4.42–4.29 (m, 2H, PhOCH_2_), 3.75 (s, 2H, PhCH_2_CO); ^13^C-NMR (75 MHz, CDCl_3_) δ 168.63, 163.26, 161.70, 155.96, 146.69, 138.72, 130.64, 130.12, 128.79, 122.51, 117.08, 115.05, 111.32, 66.65, 63.82, 39.29; HRMS (ESI) calcd. for C_22_H_20_ClN_2_O_4_ (M + H)^+^ 411.1106, found 411.1107.

*4-(2-(2-Pyridinyloxy)ethoxy)benzaldehyde-O-(2-(2-naphthalenyl)acetyl)-oxime* (**5h**). ^1^H-NMR (300 MHz, CDCl_3_) δ 8.20 (s, 1H, CH=N), 8.12–8.17 (m, 1H, Ar-H), 8.06 (d, *J* = 8.4 Hz, 1H, Ar-H), 7.93–7.75 (m, 2H, Ar-H), 7.43–7.64 (m, 7H, Ar-H), 6.95 (d, *J* = 8.8 Hz, 2H, Ar-H), 6.91–6.84 (m, 1H, Ar-H), 6.75-6.81 (m, 1H, Ar-H), 4.58–4.67 (m, 2H, PyOCH_2_), 4.40–4.30 (m, 2H, PhOCH_2_), 4.24 (s, 2H, PhCH_2_CO); ^13^C-NMR (75 MHz, CDCl_3_) δ 168.95, 163.29, 161.63, 155.90, 146.71, 138.71, 130.10, 128.73, 128.24, 128.02, 126.48, 125.84, 125.46, 123.78, 117.08, 115.01, 111.34, 66.64, 63.83, 37.88; HRMS (ESI) calcd. for C_26_H_23_N_2_O_4_ (M + H)^+^ 427.1652, found 427.1651.

*4-(2-(2-Pyridinyloxy)ethoxy)benzaldehyde-O-(2-(4-chlorophenyl)-3-methyl-1-oxobutyl)-oxime* (**5i**). ^1^H-NMR (300 MHz, CDCl_3_) δ 8.26 (s, 1H, CH=N), 8.13 (dd, *J* = 1.9 Hz, 5.0 Hz, 1H, Ar-H), 7.64 (s, 1H, Ar-H), 7.61 (s, 1H, Ar-H), 7.50–7.55 (m, 1H, Ar-H), 7.26–7.35 (m, 4H, Ar-H), 6.95 (s, 1H, Ar-H), 6.92 (s, 1H, Ar-H), 6.82–6.86 (m, 1H, Ar-H), 6.75 (d, *J* = 8.3 Hz, 1H, Ar-H), 4.56–4.88 (m, 2H, PyOCH_2_), 4.11–4.50 (m, 2H, PhOCH_2_), 3.26 (d, *J* = 10.4 Hz, 1H, CHCO), 2.29–2.45 (m, 1H, CH(CH_3_)_2_), 1.11 (d, *J* = 6.5 Hz, 3H, CHCH_3_), 0.74 (d, *J* = 6.7 Hz, 3H, CHCH_3_); ^13^C-NMR (75 MHz, CDCl_3_) δ 170.66, 163.03, 161.45, 155.89, 146.54, 138.51, 136.12, 133.08, 129.96, 129.74, 128.53, 122.44, 116.90, 114.82, 111.08, 66.44, 63.63, 57.55, 31.95, 21.20, 19.95; HRMS (ESI) calcd. for C_25_H_26_ClN_2_O_4_ (M + H)^+^ 453.1576, found 453.1575.

*4-(2-(2-Pyridinyloxy)ethoxy)benzaldehyde-O-(2-furanylcarbonyl)-oxime* (**5j**). ^1^H-NMR (300 MHz, CDCl_3_) δ 8.44 (s, 1H, CH=N), 8.15 (dd, *J* = 1.3 Hz, 5.1 Hz, 1H, Ar-H), 7.69–7.73 (m, 2H, Ar-H), 7.61–7.67 (m, 1H, Ar-H), 7.50–7.61 (m, 1H, Ar-H), 7.30 (dd, *J* = 0.7 Hz, 3.5 Hz, 1H, Ar-H), 7.00 (s, 1H, Ar-H), 6.98 (s, 1H, Ar-H), 6.86–6.90 (m, 1H, Ar-H), 6.78 (d, *J* = 8.4 Hz, 1H, Ar-H), 6.55 (dd, *J* = 1.7 Hz, 3.5 Hz, 1H, Ar-H), 4.44–4.91 (m, 2H, PyOCH_2_), 4.09–4.46 (m, 2H, PhOCH_2_); ^13^C-NMR (75 MHz, CDCl_3_) δ 163.13, 161.59, 156.31, 156.16, 146.72, 146.60, 142.85, 138.57, 130.09, 122.44, 118.62, 116.95, 114.92, 111.89, 111.14, 66.53, 63.68.; HRMS (ESI) calcd. for C_19_H_17_N_2_O_5_ (M + H)^+^ 353.1132, found 353.1133.

*4-(2-(2-Pyridinyloxy)ethoxy)benzaldehyde-O-((3-(*2-*chloro-3*,*3*,*3-trifluoro-1-propen-1-yl)-2*,*2-dimethylcyclop ropyl)carbonyl)-oxime* (**5k**). ^1^H-NMR (300 MHz, CDCl_3_) δ 8.28 (s, 1H, CH=N), 8.12 (dd, *J* = 1.9 Hz, 5.1 Hz, 1H, Ar-H), 7.59–7.75 (m, 2H, Ar-H), 7.52–7.57 (m, 1H, Ar-H), 6.94–7.01 (m, 3H, Ar-H), 6.81–6.89 (m, 1H, ArH), 6.76 (d, *J* = 8.4 Hz, 1H, CH=C), 4.58–4.79 (m, 2H, PyOCH_2_), 4.22–4.49 (m, 2H, PhOCH_2_), 2.26 (t, *J* = 8.9 Hz, 1H, CH-CH=C), 2.06 (d, *J* = 8.3 Hz, 1H, CHCO), 1.34, 1.32 (2s, 6H, (CH_3_)_2_C); ^13^C-NMR (75 MHz, CDCl_3_) δ 167.76, 163.19, 161.60, 155.45, 146.62, 138.61, 130.00, 129.64, 122.58, 118.51, 116.99, 114.96, 111.20, 66.57, 63.72, 31.16, 30.98, 29.10, 28.17, 14.75; HRMS (ESI) calcd. for C_23_H_23_ClF_3_N_2_O_4_ (M + H)^+^ 483.1293, found 483.1290.

*4-(2-(2-Pyridinyloxy)ethoxy)benzaldehyde-O-((2-chloro-3-pyridinyl)carbonyl)-oxime* (**5l**). ^1^H-NMR (300 MHz, CDCl_3_) δ 8.55 (dd, *J* = 1.9 Hz, 4.8 Hz, 1H, Ar-H), 8.46 (s, 1H, CH=N), 8.06–8.24 (m, 2H, Ar-H), 7.71–7.75 (m, 2H, Ar-H), 7.55–7.61 (m, 1H, Ar-H), 7.37 (dd, *J* = 4.8 Hz, 7.7 Hz, 1H, Ar-H), 7.00–7.04 (m, 2H, Ar-H), 6.87–6.91 (m, 1H, Ar-H), 6.79 (d, *J* = 8.4 Hz, 1H, Ar-H), 4.57–4.83 (m, 2H, PyOCH_2_), 4.02–4.54 (m, 2H, PhOCH_2_); ^13^C-NMR (75 MHz, CDCl_3_) δ 163.17, 162.38, 161.86, 157.06, 151.95, 149.65, 146.63, 139.99, 138.63, 130.26, 126.21, 122.13, 122.06, 117.00, 115.05, 111.19, 66.61, 63.70; HRMS (ESI) calcd. for C_20_H_17_ClN_3_O_4_ (M + H)^+^ 398.0902, found 398.0906.

*4-(2-(2-Pyridinyloxy)ethoxy)benzaldehyde-O-((3*,*6-dichloro-2-pyridinyl)carbonyl)-oxime*(**5m**). ^1^H-NMR (300 MHz, CDCl_3_) δ 8.48 (s, 1H, CH=N), 8.15 (dd, *J* = 1.9 Hz, 5.1 Hz, 1H, Ar-H), 7.80 (d, *J* = 8.5 Hz, 1H, Ar-H), 7.63–7.75 (m, 2H, Ar-H), 7.52–7.62 (m, 1H, Ar-H), 7.44 (d, *J* = 8.5 Hz, 1H, Ar-H), 6.98–7.02 (m, 2H, Ar-H), 6.83–6.94 (m, 1H, Ar-H), 6.78 (d, *J* = 8.4 Hz, 1H, Ar-H), 4.68–4.71 (m, 2H, PyOCH_2_), 4.36–4.39 (m, 2H, PhOCH_2_); ^13^C-NMR (75 MHz, CDCl_3_) δ 163.11, 161.79, 160.67, 157.34, 148.90, 146.58, 146.51, 140.96, 138.59, 130.24, 129.88, 127.47, 122.03, 116.97, 114.99, 111.14, 66.56, 63.67; HRMS (ESI) calcd. for C_20_H_16_Cl_2_N_3_O_4_ (M + H)^+^ 432.0512, found 432.0510.

*4-(2-(2-Pyridinyloxy)ethoxy)benzaldehyde-O-((2*,*2-dimethyl-3-(2-methyl-1-propen-1-yl)cyclopropyl)carbony l)-oxime* (**5n**). ^1^H-NMR (300 MHz, CDCl_3_) δ 8.27 (s, 1H, CH=N), 8.07–8.14 (m, 1H, Ar-H), 7.64 (d, *J* = 8.8 Hz, 2H, Ar-H), 7.44–7.58 (m, 1H, Ar-H), 6.93 (d, *J* = 8.8 Hz, 2H, Ar-H), 6.80–6.87 (m, 1H, Ar-H), 6.70–6.79 (m, 1H, Ar-H), 4.80–5.03 (m, 1H, CH=C(CH_3_)_2_), 4.51–4.74 (m, 2H, PyOCH_2_), 4.14–4.42 (m, 2H, PhOCH_2_), 2.07–2.23 (m, 1H, CH-CH=C), 1.69 (s, 6H, C=C(CH_3_)_2_), 1.47 (d, *J* =5.3 Hz, 1H, CHCO), 1.31, 1.16 (2s, 6H, (CH_3_)_2_C); ^13^C-NMR (75 MHz, CDCl_3_) δ 169.94, 163.14, 161.31, 154.76, 146.57, 138.54, 135.84, 129.85, 122.95, 120.63, 116.91, 114.83, 111.15, 66.48, 63.69, 33.11, 32.89, 29.18, 25.40, 21.97, 20.25, 18.38; HRMS (ESI) calcd. for C_24_H_29_N_2_O_4_ (M + H)^+^ 409.2122, found 409.2122.

*4-(2-(2-Pyridinyloxy)ethoxy)benzaldehyde-O-(1-oxobutyl)-oxime* (**5o**). ^1^H-NMR (300 MHz, CDCl_3_) δ 8.29 (s, 1H, CH=N), 8.13–8.16 (m, 1H, Ar-H), 7.62–7.75 (m, 2H, Ar-H), 7.50–7.61 (m, 1H, Ar-H), 6.93–7.05 (m, 2H, Ar-H), 6.86–6.90 (m, 1H, Ar-H), 6.79 (d, *J* = 8.4 Hz, 1H, Ar-H), 4.57–4.84 (m, 2H, PyOCH_2_), 4.24–4.53 (m, 2H, PhOCH_2_), 2.44 (t, *J* = 7.4 Hz, CH_2_CO), 1.71–1.79 (m, 2H, CH_3_CH_2_), 1.01 (t, *J* = 7.4 Hz, 3H, CH_3_CH_2_); ^13^C-NMR (75 MHz, CDCl_3_) δ 171.10, 163.18, 161.45, 155.33, 146.61, 138.61, 129.94, 122.78, 116.98, 114.90, 111.20, 66.55, 63.73, 34.60, 18.27, 13.56; HRMS (ESI) calcd. for C_18_H_21_N_2_O_4_ (M + H)^+^ 329.1496, found 329.1500.

*4-(2-(2-Pyridinyloxy)ethoxy)benzaldehyde-O-acetyl-oxime* (**5p**). ^1^H-NMR (300 MHz, CDCl_3_) δ 8.29 (s, 1H, CH=N), 8.06–8.22 (m, 1H, Ar-H), 7.62–7.73 (m, 2H, Ar-H), 7.55–7.61 (m, 1H, Ar-H), 6.93–7.04 (m, 2H, Ar-H), 6.87–6.91 (m, 1H, Ar-H), 6.79 (d, *J* = 8.4 Hz, 1H, Ar-H), 4.56–4.80 (m, 2H, PyOCH_2_), 4.18–4.45 (m, 2H, PhOCH_2_), 2.21 (s, 3H, COCH_3_); ^13^C-NMR (75 MHz, CDCl_3_) δ 168.66, 163.17, 161.49, 155.30, 146.59, 138.63, 129.94, 122.67, 116.98, 114.93, 111.20, 66.56, 63.74, 19.47; HRMS (ESI) calcd. for C_16_H_17_N_2_O_4_ (M + H)^+^ 301.1183, found 301.1186.

*4-(2-(2-Pyridinyloxy)ethoxy)benzaldehyde-O-(2-thienylcarbonyl)-oxime* (**5q**). ^1^H-NMR (300 MHz, CDCl_3_) δ 8.46 (s, 1H, CH=N), 8.15–8.18 (m, 1H, Ar-H), 7.94 (dd, *J* = 1.2 Hz, 3.8 Hz, 1H, Ar-H), 7.68–7.80 (m, 2H, Ar-H), 7.49–7.68 (m, 2H, Ar-H), 7.16 (dd, *J* = 3.8 Hz, 5.0 Hz, 1H, Ar-H), 6.95–7.04 (m, 2H, Ar-H), 6.86–6.95 (m, 1H, Ar-H), 6.81 (d, *J* = 8.4 Hz, 1H, Ar-H), 4.55–4.94 (m, 2H, PyOCH_2_), 4.20–4.53 (m, 2H, PhOCH_2_); ^13^C-NMR (75 MHz, CDCl_3_) δ 163.16, 161.57, 159.68, 156.08, 146.61, 138.61, 134.06, 132.87, 131.32, 130.11, 127.78, 122.56, 116.98, 114.95, 111.20, 66.55, 63.72; HRMS (ESI) calcd. for C_19_H_17_N_2_O_4_S (M + H)^+^ 369.0904, found 369.0906.

*4-(2-(2-Pyridinyloxy)ethoxy)benzaldehyde-O-(3-pyridinylcarbonyl)-oxime* (**5r**). ^1^H-NMR (300 MHz, CDCl_3_) δ 9.31–9.36 (m, 1H, Ar-H), 8.85 (dd, *J* = 4.9 Hz, 1.7 Hz, 1H, Ar-H), 8.53 (s, 1H, CH=N), 8.39–8.43 (m, 1H, Ar-H), 8.17–8.19 (dd, *J* = 1.3 Hz, 5.1 Hz, 1H, Ar-H), 7.75–7.80 (m, 2H, Ar-H), 7.58–7.64 (m, 1H, Ar-H), 7.47 (dd, *J* = 4.9 Hz, 8.0 Hz, 1H, Ar-H), 6.97–7.15 (m, 2H, Ar-H), 6.87–6.95 (m, 1H, Ar-H), 6.82 (d, *J* = 8.4 Hz, 1H, Ar-H), 4.64–4.80 (m, 2H, PyOCH_2_), 4.33–4.45 (m, 2H, PhOCH_2_); ^13^C-NMR (75 MHz, CDCl_3_) δ 163.25, 162.74, 161.85, 156.80, 153.68, 150.70, 146.69, 138.68, 137.11, 130.28, 124.93, 123.41, 122.39, 117.05, 115.10, 111.28, 66.67, 63.77; HRMS (ESI) calcd. for C_20_H_18_N_3_O_4_ (M + H)^+^ 364.1292, found 364.1294.

*4-(2-(2-Pyridinyloxy)ethoxy)benzaldehyde-O-((2*,*2*,*3*,*3 tetramethylcyclopropyl)carbonyl)-oxime* (**5s**). ^1^H-NM R (300 MHz, CDCl_3_) δ 8.30 (s, 1H, CH=N), 8.16 (dd, *J* = 1.5 Hz, 5.1 Hz, 1H, Ar-H), 7.63–7.76 (m, 2H, Ar-H), 7.53–7.62 (m, 1H, Ar-H), 6.95–7.01 (m, 2H, Ar-H), 6.85–6.93 (m, 1H, Ar-H), 6.80 (d, *J* = 8.4 Hz, 1H, Ar-H), 4.59–4.85 (m, 2H, PyOCH_2_), 4.18–4.54 (m, 2H, PhOCH_2_), 1.33 (s, 6H, C(CH_3_)_2_), 1.29 (s, 1H, CHCO), 1.25 (s, 6H, C(CH_3_)_2_); ^13^C-NMR (75 MHz, CDCl_3_) δ 169.36, 163.21, 161.28, 154.41, 146.62, 138.61, 129.84, 123.21, 116.97, 114.86, 111.24, 66.54, 63.78, 33.80, 30.97, 23.42, 16.46; HRMS (ESI) calcd. for C_22_H_27_N_2_O_4_ (M + H)^+^ 383.1965, found 383.1968.

### 3.2. Biological Evaluation

#### 3.2.1. Insecticidal Activity against *Plutella xylostella*

The test samples were dissolved in acetone and then diluted with water to obtain a final concentration of 600 μg/mL. Pyriproxyfen was used as a positive control whereas acetone was used as a negative control. Cabbage leaves were dipped into the obtained solutions for 5 s. The larvae of *P. xyllostella* were fed with the discs. Cohorts of about 15 *P. xyllostella* were treated each time and bioassays were repeated in triplicate. After 72 h, the numbers of knocked down larvae were counted and recorded [25,26].

#### 3.2.2. Insecticidal Activity against *Myzus persicae*

The test samples were dissolved in *N*,*N*-dimethylformamide and then diluted to the required concentration with water. Peach leaves with about 40 *Myzus persicae* were dipped for 5 s into a solution with compound concentrations ranging from 200 to 600 μg/mL. After removing any excess solution on the leaves, *Myzus persicae* were raised in the leaves at 25 °C and 85% relative humidity for 48 h. Each experiment for every compound was repeated in triplicates [26].

#### 3.2.3. Ovicidal Activity against *Helicoverpa armigera* Eggs

The test samples were dissolved in *N*,*N*-dimethylformamide and then diluted with water to final concentrations of 600 μg/mL and 200 μg/mL. Pieces of gauze with about 60 *Helicoverpa armigera* eggs were dipped into a corresponding solution for 10 s. After drying, the gauzes were moved into disks. The disks were then kept at 25 °C in plastic Petri dishes with moist filter paper. The number of eggs hatched in the control and treatment samples were recorded and the percentage of ovicidal activity was calculated.

## 4. Conclusions

In summary, nineteen oxime ester derivatives, **5a**–**5s,** were synthesized. A preliminary evaluation of the ovicidal and insecticidal activities of the target compounds was performed. The bioassay results demonstrated that most of the synthesized compounds displayed moderate insecticidal activities and high ovicidal activities at a concentration of 600 μg/mL. Some derivatives, including compounds **5o** and **5p** bearing alkyl groups, exhibited respective ovicidal activities of 67.8% and 79.5% against *Helicoverpa armigera* eggs at a lower concentration of 200 μg/mL, higher than that of the reference compound, pyriproxyfen. Among the synthesized compounds, compounds **5j** and **5s** demonstrated broad biological activity spectra, with potential insecticidal activities against *P. xylostella* and *Myzus persicae*, and satisfactory ovicidal activities against *Helicoverpa armigera* eggs. Encouraged by the results described above, our laboratory is currently studying similar derivatives based on pesticide metabolites. A report of these advanced studies is currently being written up and will be published in due course.

## Figures and Tables

**Table 1:** Physical data for compounds **5a**–**5s**.

Compound	R	Formula	Status	m.p./°C	Yield (%)
**5a**	Ph-	C_21_H_18_N_2_O_4_	White solid	102–104	87
**5b**	4-OCH_3_-Ph-	C_22_H_20_N_2_O_5_	White solid	101–103	91
**5c**	2-F-Ph-	C_21_H_17_FN_2_O_4_	White solid	107–109	91
**5d**	4-OCH_3_-Ph-CH_2_-	C_23_H_22_N_2_O_5_	White solid	97–99	90
**5e**	4-CH_3_-Ph-CH_2_-	C_23_H_22_N_2_O_4_	White solid	102–104	88
**5f**	4-OC_2_H_5_-Ph-	C_23_H_22_N_2_O_5_	White solid	103–105	83
**5g**	2,4-Cl-Ph-CH_2_-	C_22_H_18_Cl_2_N_2_O_4_	White solid	102–104	71
**5h**	β-Naphthyl-CH_2_-	C_26_H_22_N_2_O_4_	White solid	112–114	94
**5i**	2-(4-chlorophenyl)-3-methylpropyl-	C_25_H_25_ClN_2_O_4_	Pale yellow solid	75–77	85
**5j**	2-furyl-	C_19_H_16_N_2_O_5_	White solid	130–132	92
**5k**	3-(2-Cl-3,3,3-F-1-propenyl)-2,2-Me-cycloproyl-	C_23_H_22_ClF_3_N_2_O_4_	Yellow solid	141–143	86
**5l**	3-(2-Cl-Py)-	C_20_H_16_ClN_3_O_4_	White solid	133–135	90
**5m**	2-(3,6-Cl-Py)-	C_20_ H_15_Cl_2_N_3_O_4_	Yellow solid	88–90	87
**5n**	2,2-Me-3-(2-Me-1-propenyl)-cycloproyl-	C_24_H_28_N_2_O_4_	White solid	99–101	85
**5o**	CH_3_CH_2_CH_2_-	C_18_H_20_N_2_O_4_	White solid	71–73	93
**5p**	CH_3_-	C_16_H_16_N_2_O_4_	White solid	85–87	96
**5q**	2-thienyl-	C_19_H_16_N_2_O_4_S	White solid	132–134	88
**5r**	3-Py-	C_20_H_17_N_3_O_4_	White solid	163–165	90
**5s**	2,2,3,3-Me-cyclopropyl-	C_22_H_26_N_2_O_4_	Yellow solid	91–93	89

**Table 2:** Insecticidal and ovicidal activities of oxime ester derivatives (activity provided in %).

Compound	*Myzus persicae*		*Plutella xyllostella*	*Helicoverpa armigera* Eggs	
	600 μg/mL	200 μg/mL	600 μg/mL	600 μg/mL	200 μg/mL
**5a**	3.6 ± 0.7 ^a^		41.7± 1.9	20.9 ± 0.7	
**5b**	13.3 ± 1.6		7.7 ± 2.1	23.2 ± 0.8	
**5c**	12.6 ± 2.1		38.5 ± 3.0	28.8 ± 1.1	
**5d**	8.7 ± 1.5		20.0 ± 1.3	15.3 ± 0.4	
**5e**	11.3 ± 0.4		0 ± 0.0	47.6 ± 0.1	
**5f**	15.7 ± 0.2		10.0 ± 2.5	9.2 ± 1.8	
**5g**	11.6 ± 1.0		0 ± 0.0	55.7 ± 3.1	
**5h**	6.7 ± 0.4		40.0 ± 2.0	51.6 ± 3.3	
**5i**	11.3 ± 0.4		33.3 ± 1.9	45.7 ± 1.7	
**5j**	87.5 ± 5.3	53.8 ± 4.5	68.7 ± 3.6	100.0 ± 0.0	45.3 ± 2.7
**5k**	45.2 ± 2.6		26.7 ± 1.8	67.6 ± 3.3	
**5l**	49.4 ± 5.7		15.4 ± 0.4	62.4 ± 3.1	
**5m**	58.1 ± 1.9		34.3 ± 1.0	28.5 ± 0.8	
**5n**	39.6 ± 2.7		15.1 ± 0.8	43.3 ± 3.5	
**5o**	57.7 ± 0.4		9.6 ± 2.1	100.0 ± 0.0	67.8 ± 5.2
**5p**	53.2 ± 3.6		12.8 ± 1.3	100.0 ± 0.0	79.5 ± 4.2
**5q**	73.6 ± 2.9	36.4 ± 2.1	43.1 ± 3.3	100.0 ± 0.0	37.1 ± 1.6
**5r**	25.9 ± 2.2		27.7 ± 1.0	73.4 ± 5.1	
**5s**	72.8 ± 1.2	41.3 ± 2.4	57.1 ± 1.4	100.0 ± 0.0	64.3 ± 2.0
**Pyriproxyfen**	68.3 ± 4.7	38.5 ± 1.3	76.0 ± 5.3	100.0 ± 0.0	54.8 ± 1.7

^a^ Each value represents the mean ± the standard error of the mean (SEM) of three replicates.

## Supplementary Materials

Supplementary materials are available online. ^1^H- and ^13^C-NMR spectra of all compounds are presented in the Supporting Information section.
