# Supplementary material for: Ovicidal and Insecticidal Activities of Pyriproxyfen Derivatives with an Oxime Ester Group
Source: Molecules. 2017 Jun 8;22(6):958. doi: 10.3390/molecules22060958 (PMC6152621; doi:10.3390/molecules22060958)

molecules

Supplementary Materials:

Ovicidal and Insecticidal Activities of Pyriproxyfen Derivatives with an Oxime Ester group

Guo-Shao Sun, Xin Xu, Shu-Hui Jin *, Le Lin and Jian-Jun Zhang *

Department of Applied Chemistry, College of Science, China Agricultural University, Beijing 100193, China; guoshaosun@163.com (G.S.); lijinming0711@cau.edu.cn (X.X.); linlel@163.com (L.L.);

* Correspondence: shuhuij@cau.edu.cn (S.-H.J.); zhangjianjun@cau.edu.cn (J.-J.Z.); Tel.: +86-10-6273-2219 (J.-J.Z.); +86-10-6273-3003 (S.-H.J.).

**Contents**	**Page**
^{1}H-NMR spectrum of compound **2.**	S4
^{1}H-NMR spectrum of compound **3.**	S4
^{1}H-NMR spectrum of compound **4.**	S5
^{13}C-NMR spectrum of compound **4.**	S5
^{1}H-NMR spectrum of compound **5a.**	S6
^{13}C-NMR spectrum of compound **5a.**	S6
HRMS of compound **5a.**	S7
^{1}H-NMR spectrum of compound **5b.**	S7
^{13}C-NMR spectrum of compound **5b.**	S8
HRMS of compound **5b.**	S8
^{1}H-NMR spectrum of compound **5c.**	S9
^{13}C-NMR spectrum of compound **5c.**	S9
HRMS of compound **5c.**	S10
^{1}H-NMR spectrum of compound **5d.**	S10
^{13}C-NMR spectrum of compound **5d.**	S11
HRMS of compound **5d.**	S11
^{1}H-NMR spectrum of compound **5e.**	S12
^{13}C-NMR spectrum of compound **5e.**	S12
HRMS of compound **5e.**	S13
^{1}H-NMR spectrum of compound **5f.**	S13
^{13}C-NMR spectrum of compound **5f.**	S14
HRMS of compound **5f.**	S14
^{1}H-NMR spectrum of compound **5g.**	S15

^{13}C-NMR spectrum of compound **5g.**	S15
HRMS of compound **5g.**	S16
^{1}H-NMR spectrum of compound **5h.**	S16
^{13}C-NMR spectrum of compound **5h.**	S17
HRMS of compound **5h.**	S17
^{1}H-NMR spectrum of compound **5i.**	S18
^{13}C-NMR spectrum of compound **5i.**	S18
HRMS of compound **5i.**	S19
^{1}H-NMR spectrum of compound **5j.**	S19
^{13}C-NMR spectrum of compound **5j.**	S20
HRMS of compound **5j.**	S20
^{1}H-NMR spectrum of compound **5k.**	S21
^{13}C-NMR spectrum of compound **5k.**	S21
HRMS of compound **5k.**	S22
^{1}H-NMR spectrum of compound **5l.**	S22
^{13}C-NMR spectrum of compound **5l.**	S23
HRMS of compound **5l.**	S23
^{1}H-NMR spectrum of compound **5m.**	S24
^{13}C-NMR spectrum of compound **5m.**	S24
HRMS of compound **5m.**	S25
^{1}H-NMR spectrum of compound **5n.**	S25
^{13}C-NMR spectrum of compound **5n.**	S26
HRMS of compound **5n.**	S26
^{1}H-NMR spectrum of compound **5o.**	S27
^{13}C-NMR spectrum of compound **5o.**	S27
HRMS of compound **5o.**	S28
^{1}H-NMR spectrum of compound **5p.**	S28
^{13}C-NMR spectrum of compound **5p.**	S29
HRMS of compound **5p.**	S29
^{1}H-NMR spectrum of compound **5q.**	S30
^{13}C-NMR spectrum of compound **5q.**	S30
HRMS of compound **5q.**	S31
^{1}H-NMR spectrum of compound **5r.**	S31
^{13}C-NMR spectrum of compound **5r.**	S32

HRMS of compound **5r.**	S32
^{1}H-NMR spectrum of compound **5s.**	S33
^{13}C-NMR spectrum of compound **5s.**	S33
HRMS of compound **5s.**	S34

^{1}H-NMR spectrum of compound **2.**

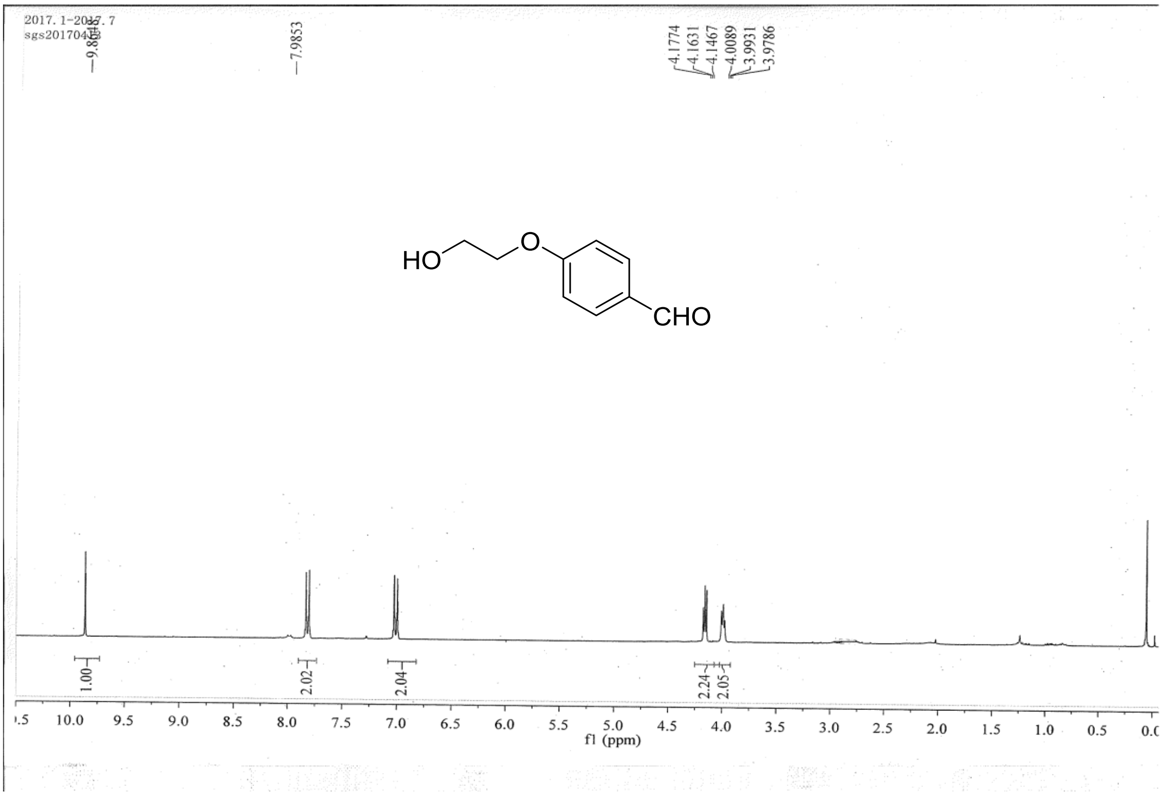

^{1}H-NMR spectrum of compound **3.**

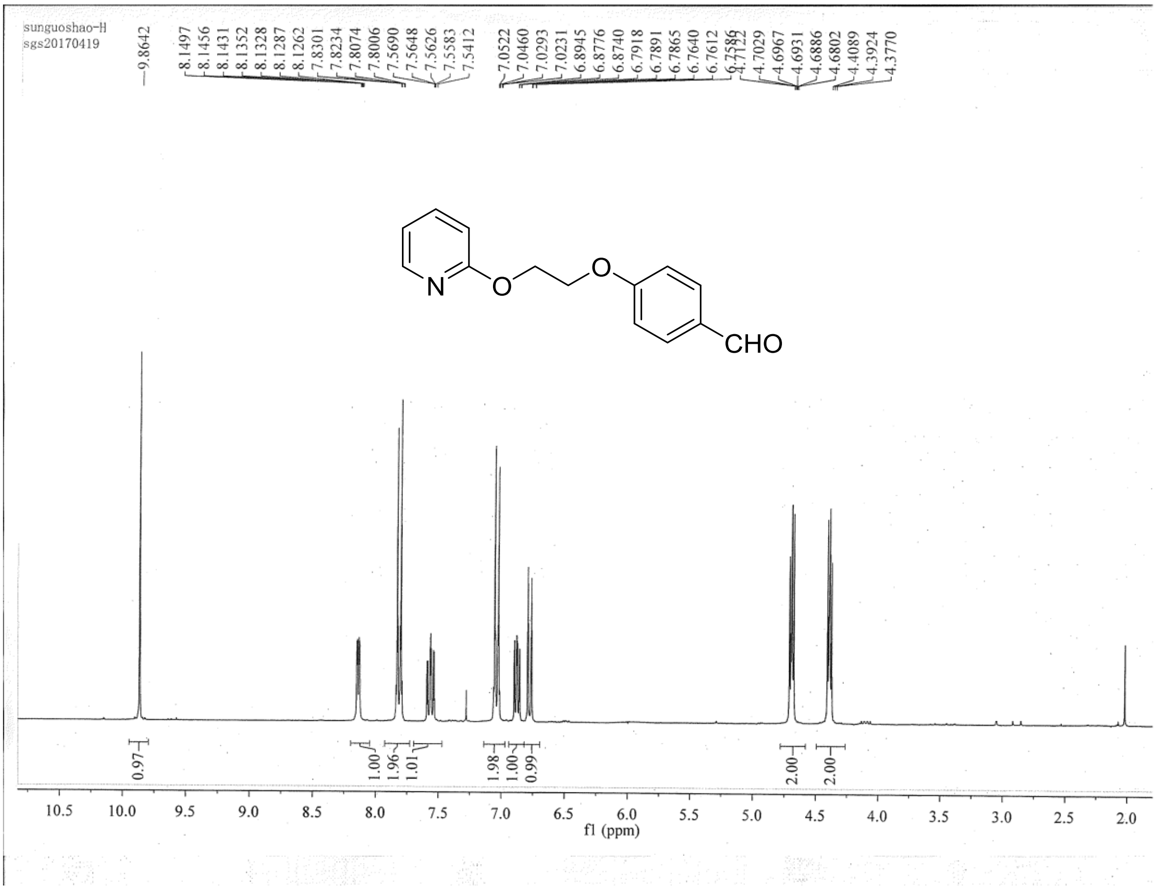

^{1}H-NMR spectrum of compound **4.**

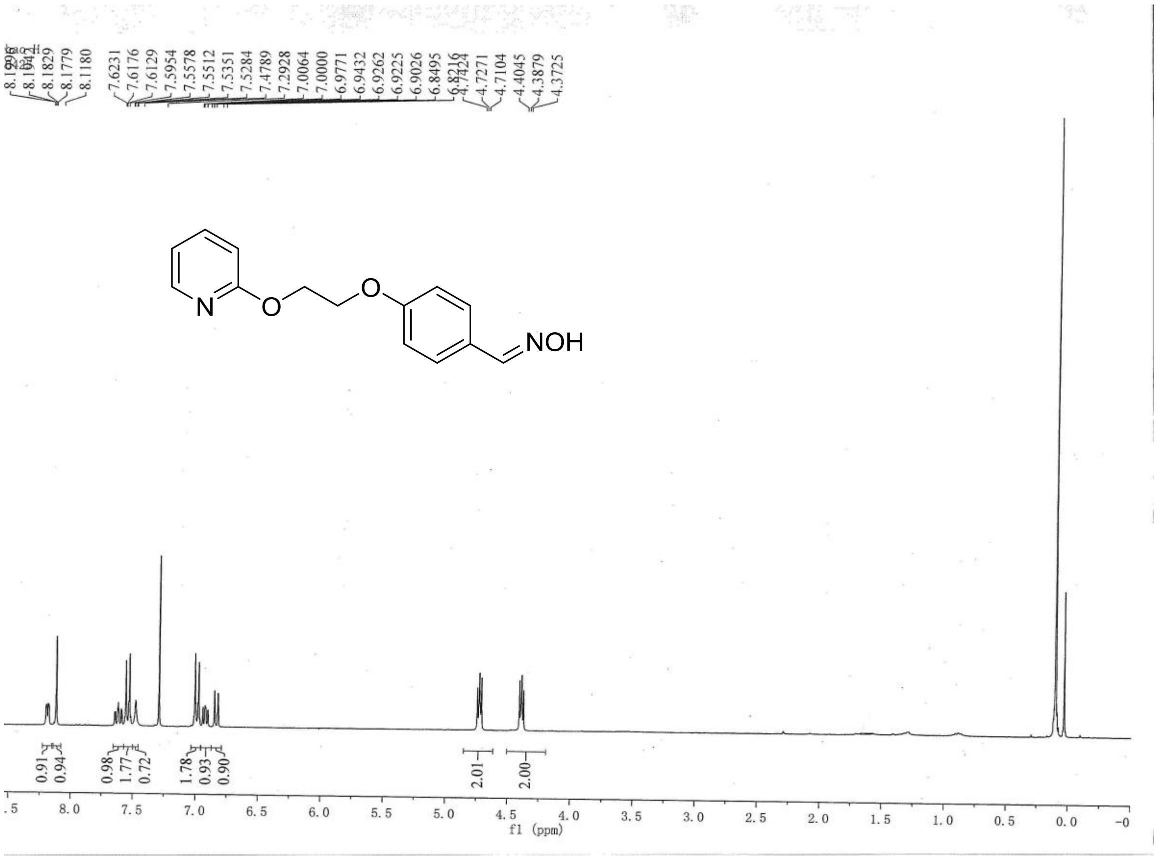

^{13}C-NMR spectrum of compound **4.**

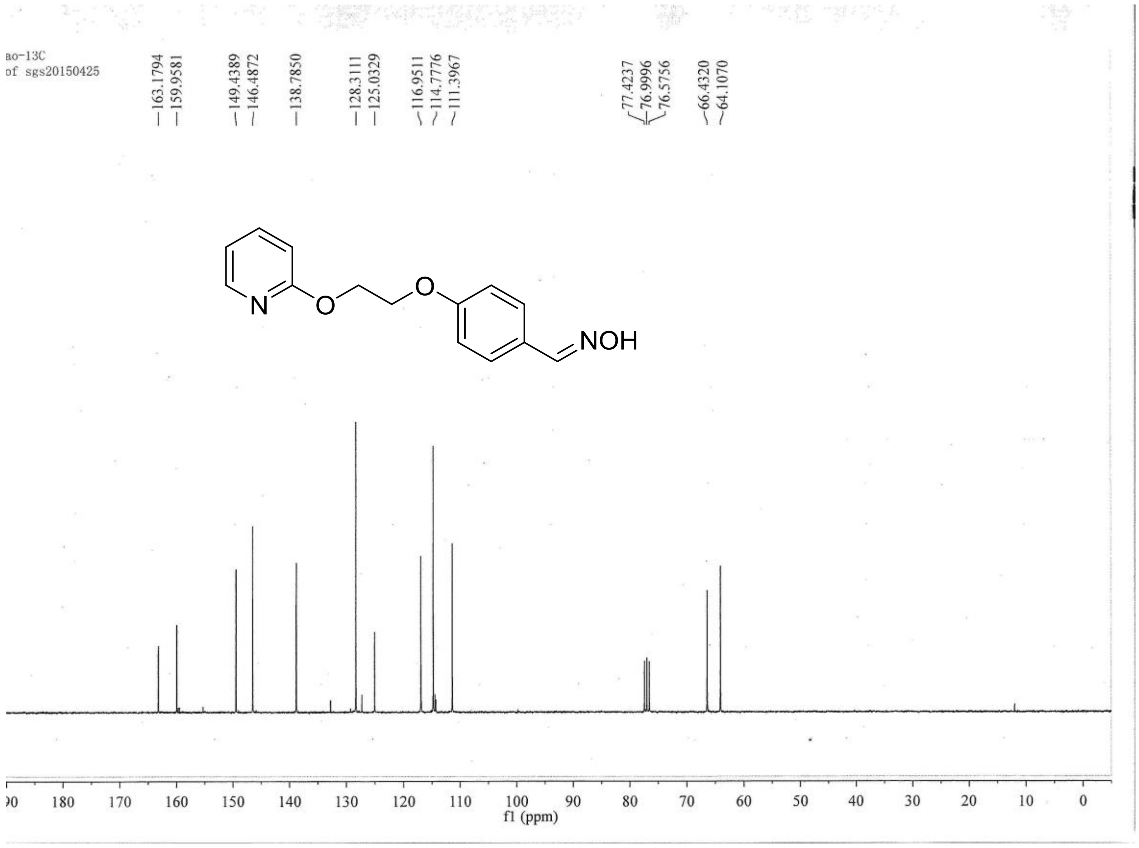

^{1}H-NMR spectrum of compound **5a.**

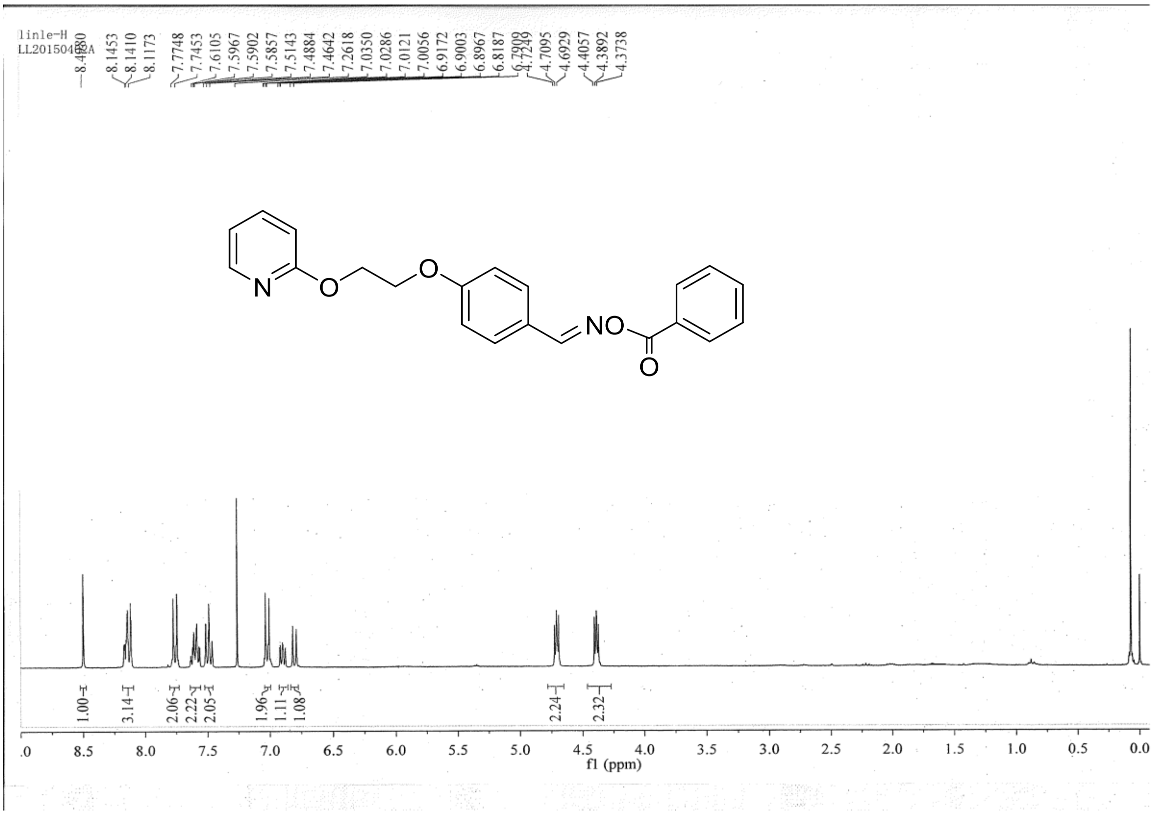

^{13}C-NMR spectrum of compound **5a.**

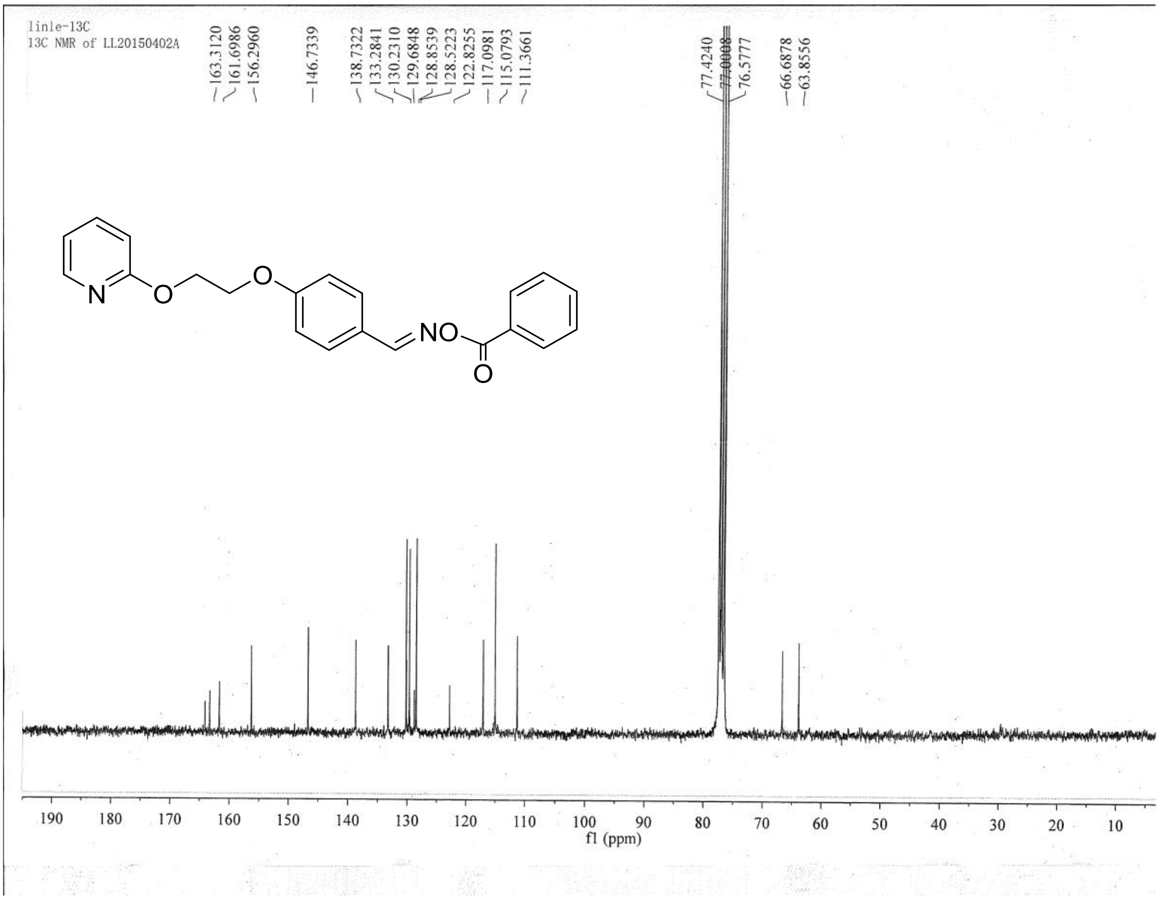

HRMS of compound **5a.**

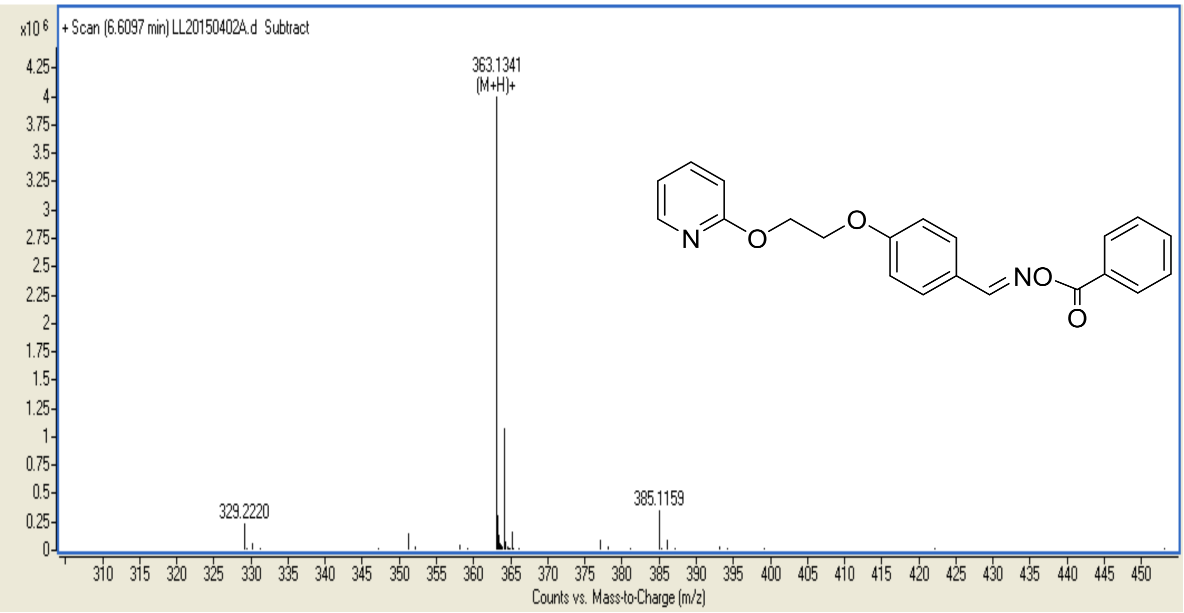

^{1}H-NMR spectrum of compound **5b.**

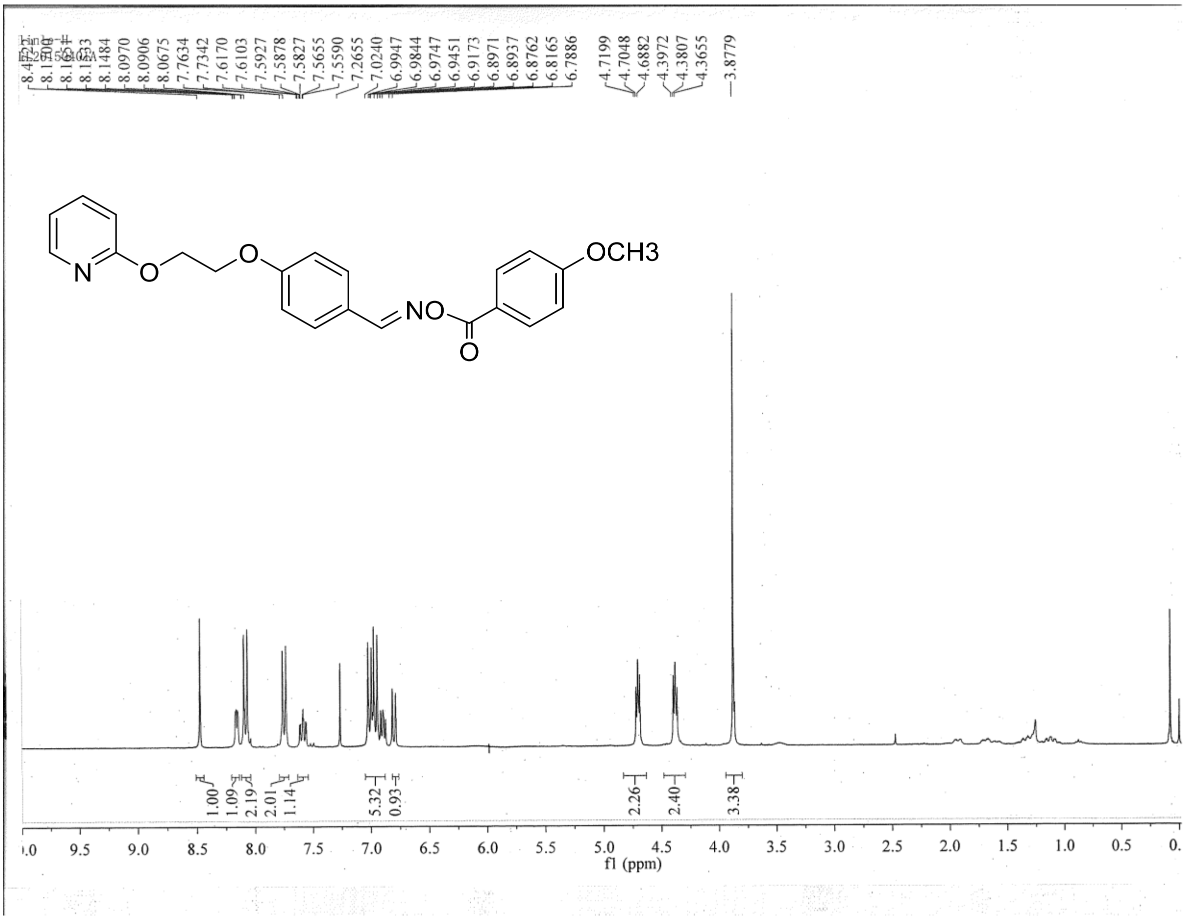

^{13}C-NMR spectrum of compound **5b.**

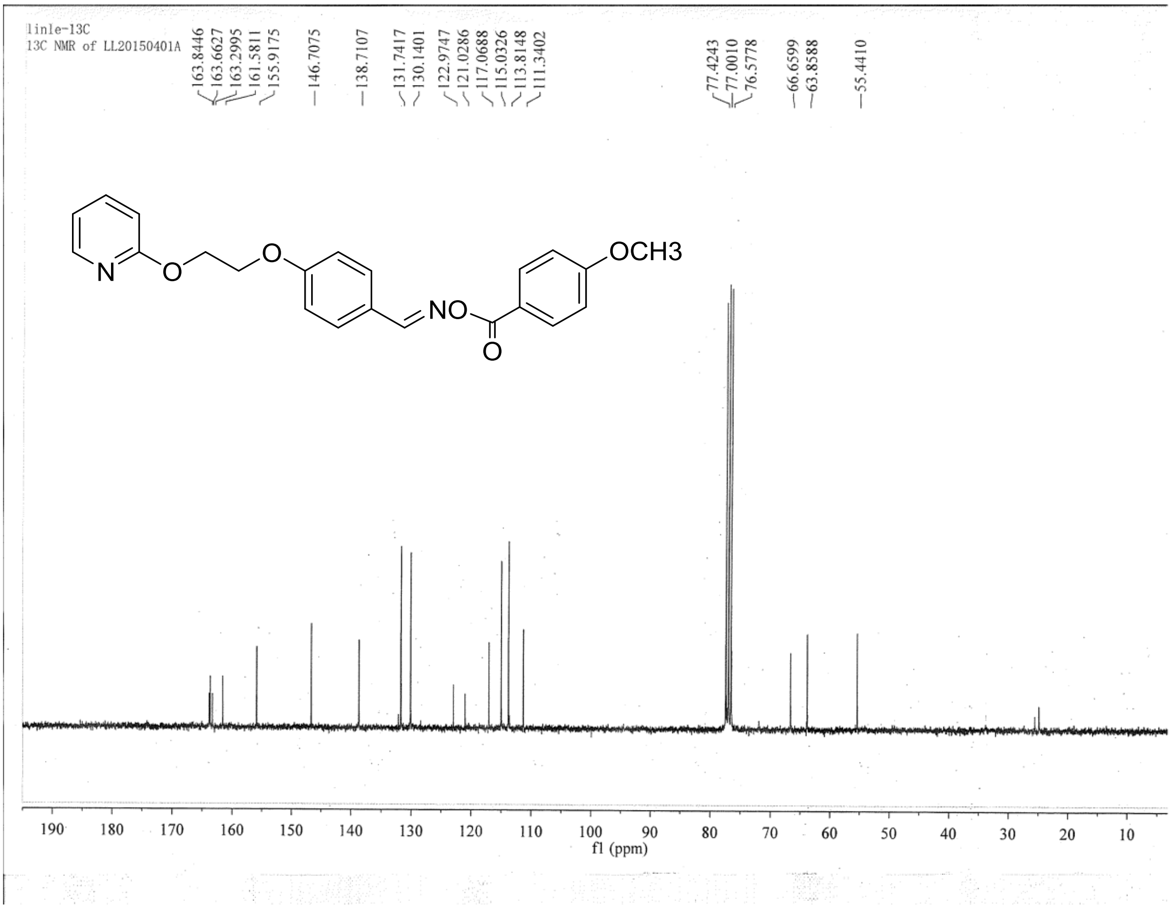

HRMS of compound **5b.**

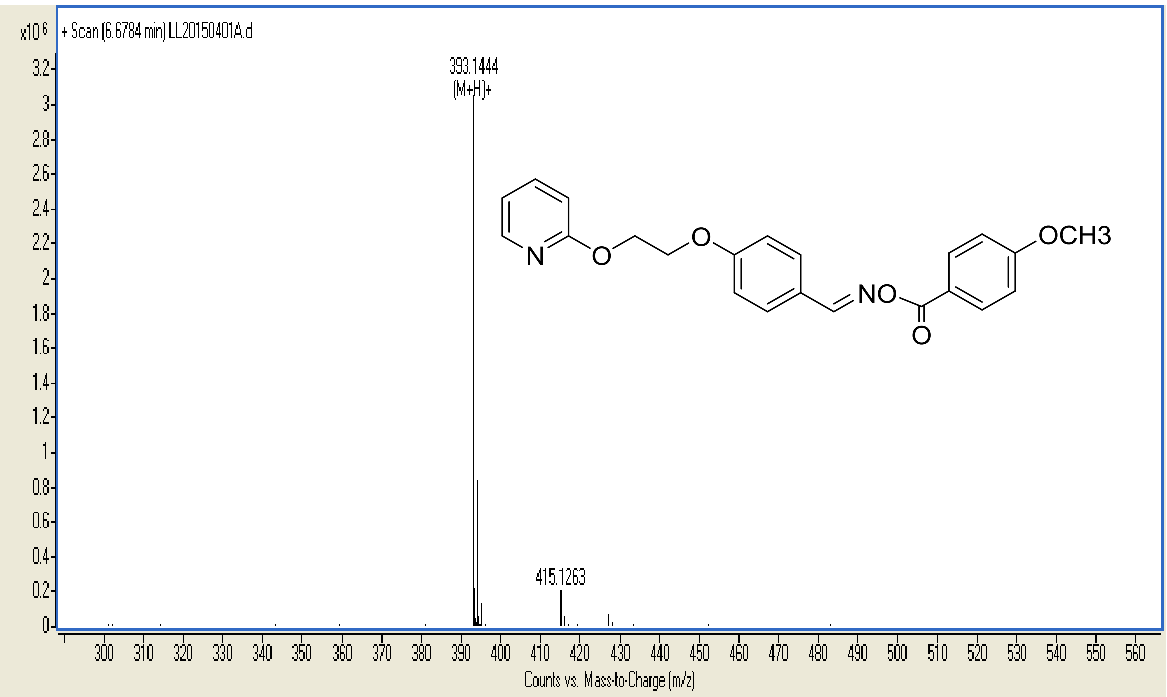

^{1}H-NMR spectrum of compound **5c.**

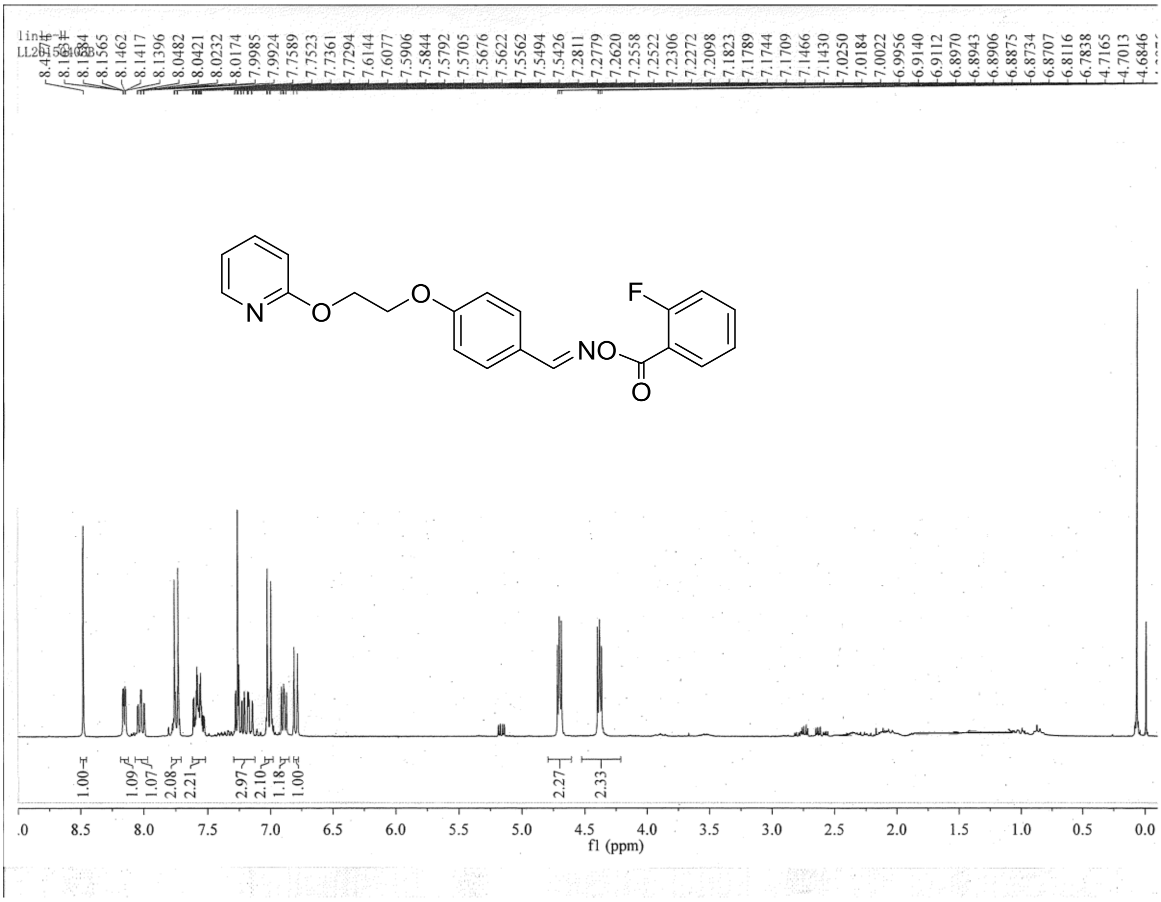

^{13}C-NMR spectrum of compound **5c.**

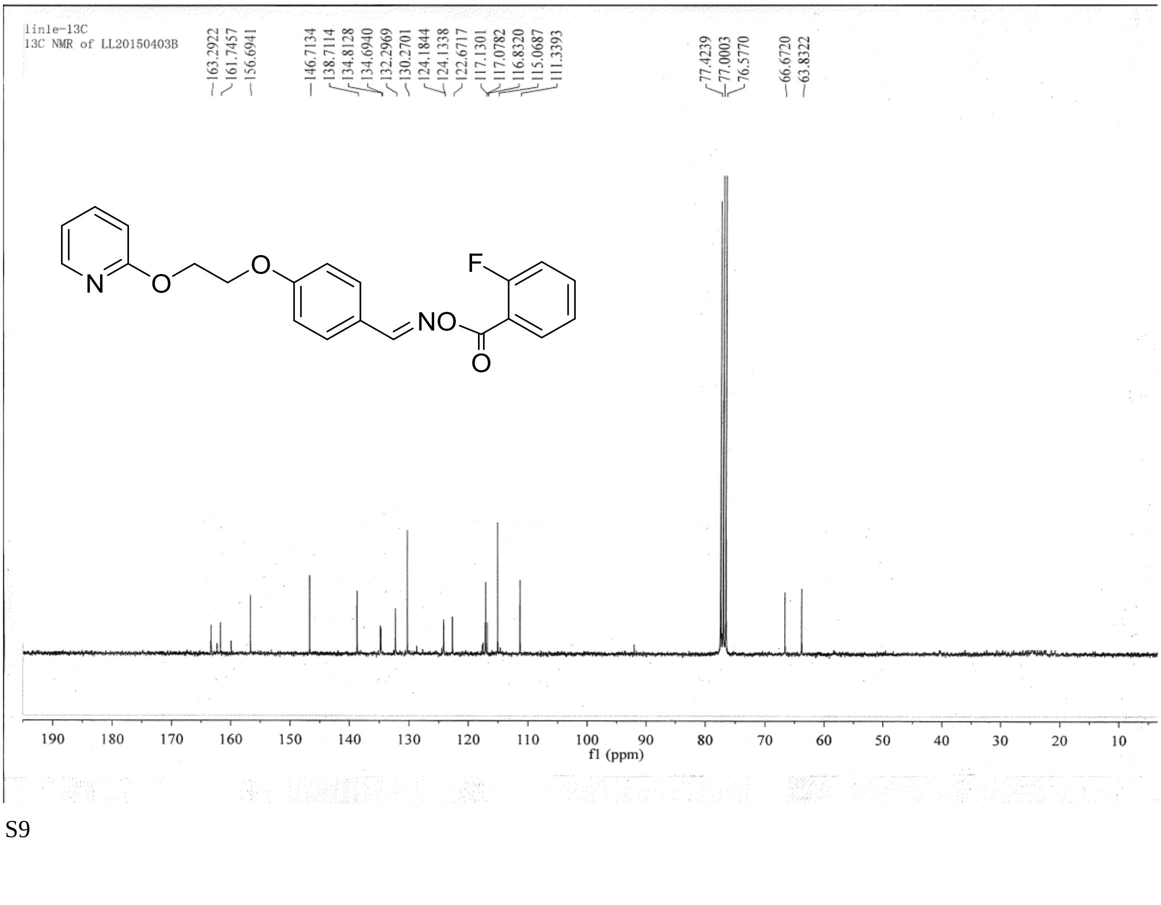

HRMS of compound **5c.**

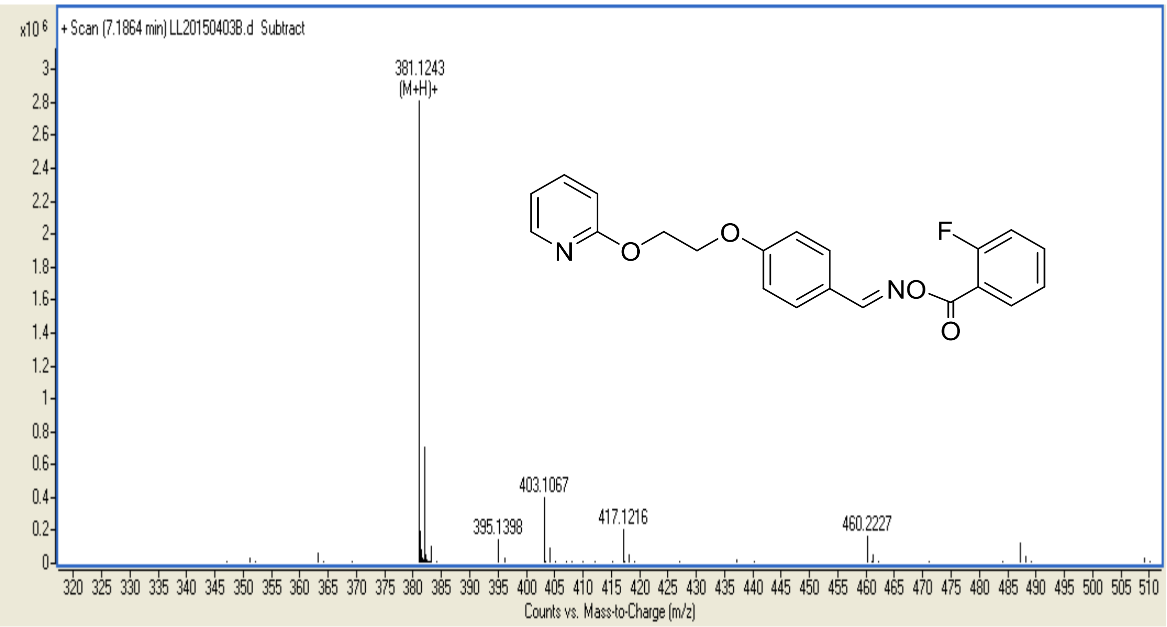

^{1}H-NMR spectrum of compound **5d.**

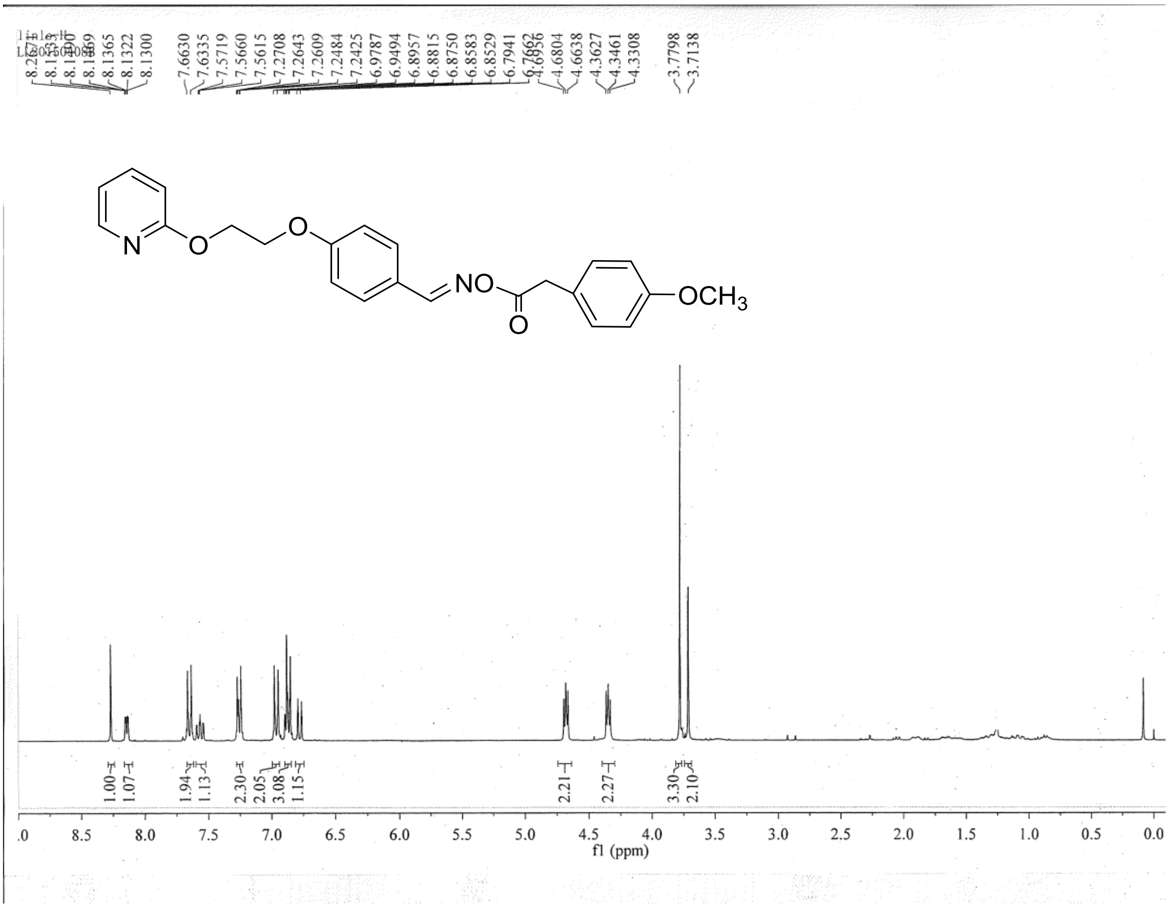

^{13}C-NMR spectrum of compound **5d.**

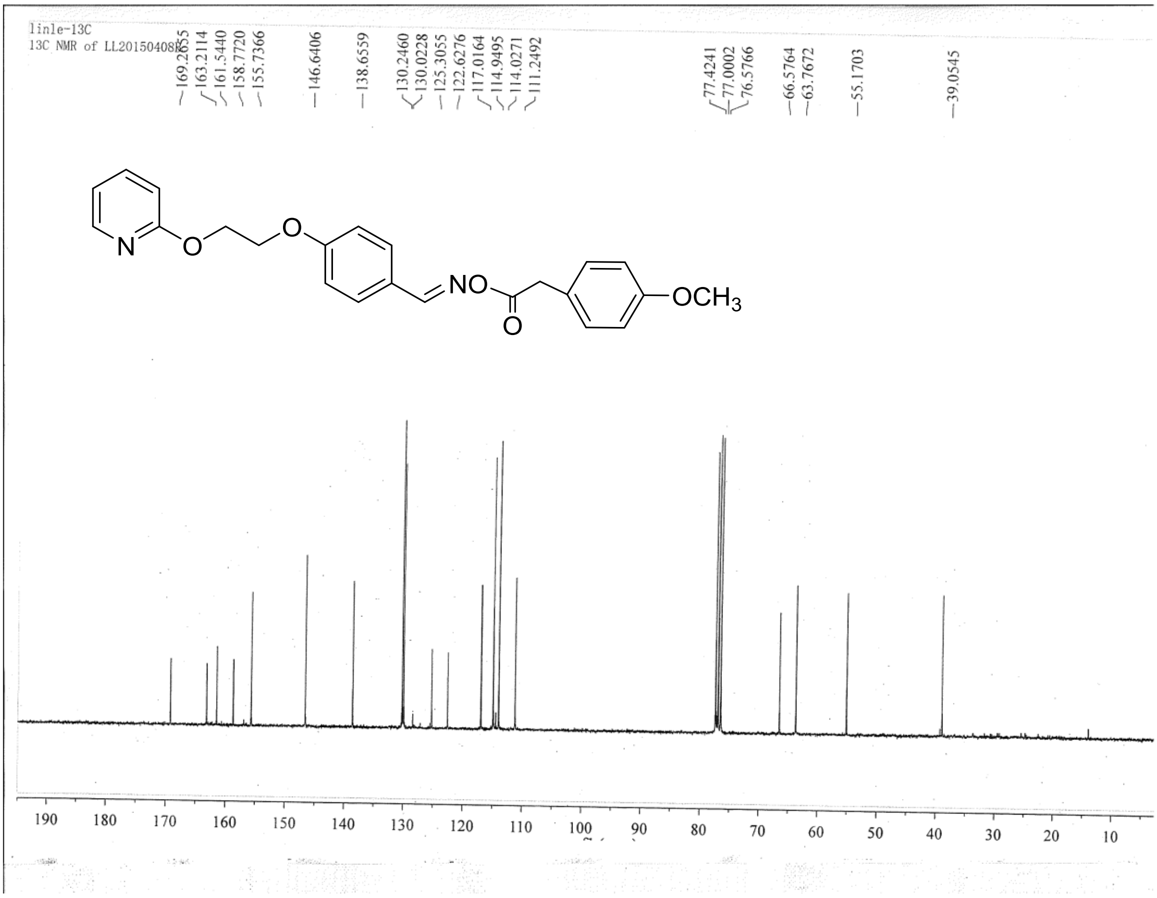

HRMS of compound **5d.**

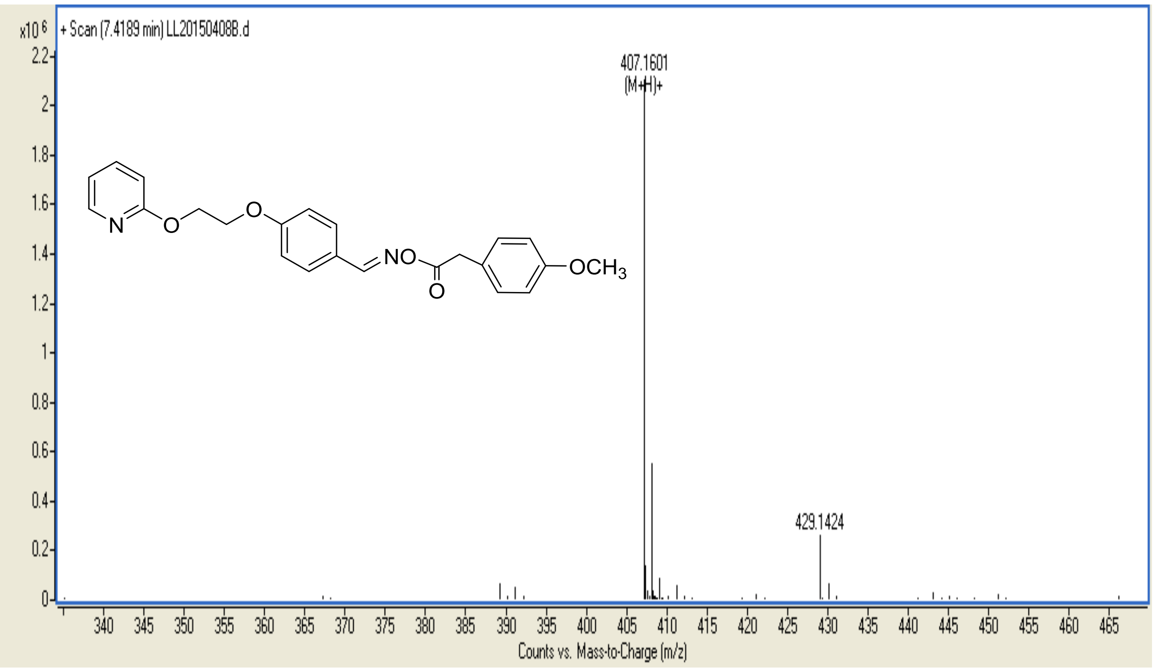

^{1}H-NMR spectrum of compound **5e.**

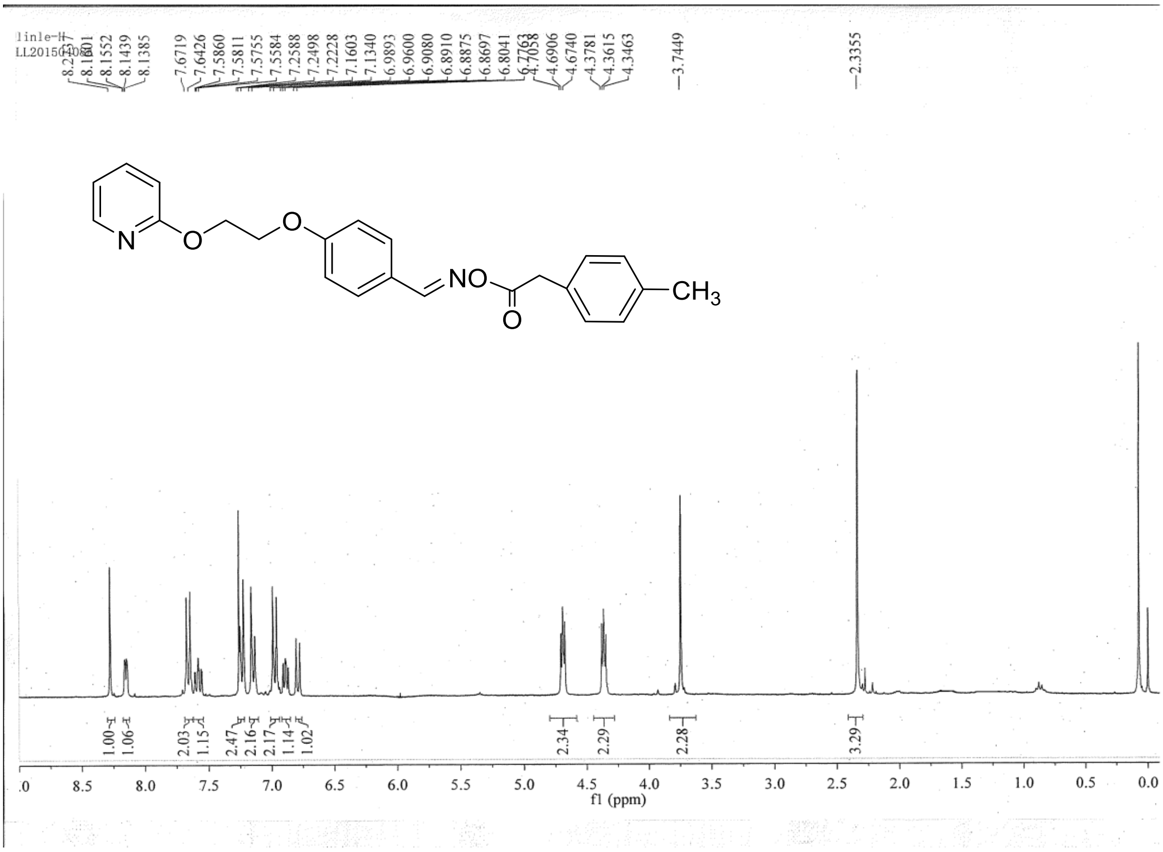

^{13}C-NMR spectrum of compound **5e.**

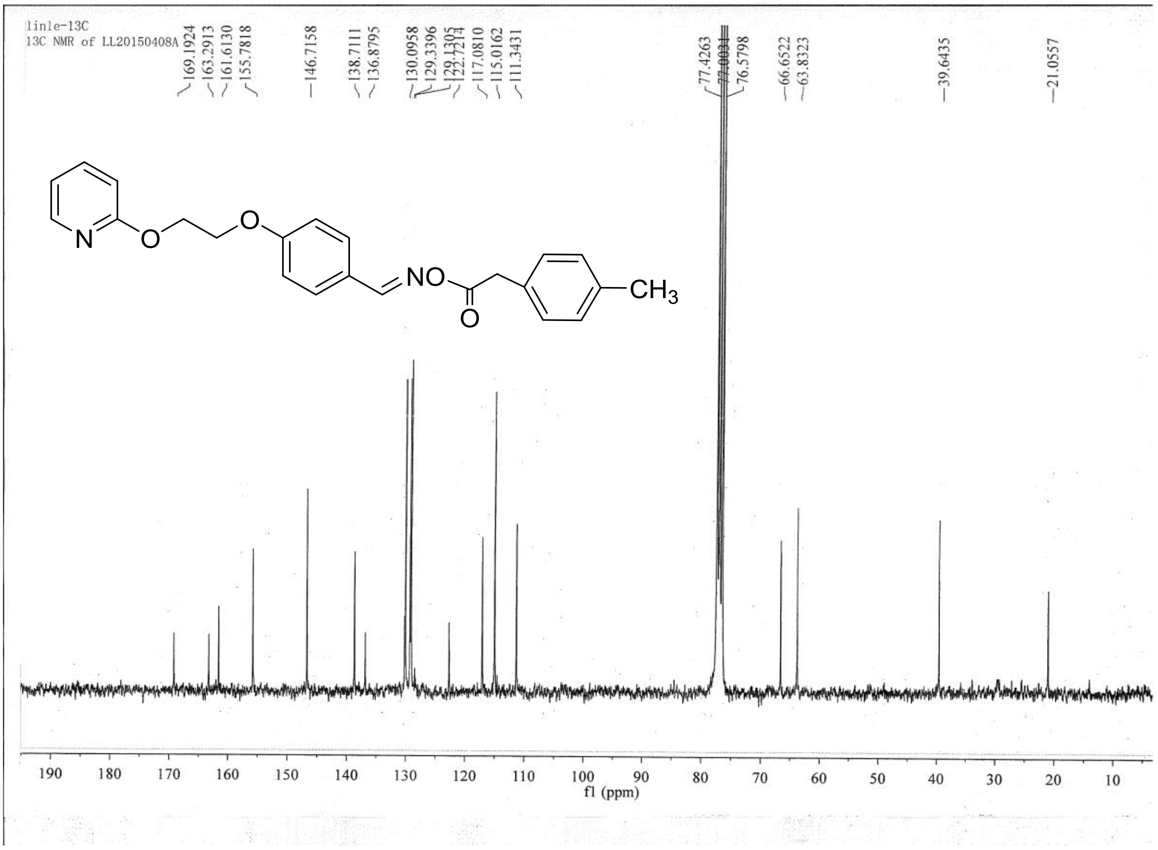

HRMS of compound **5e.**

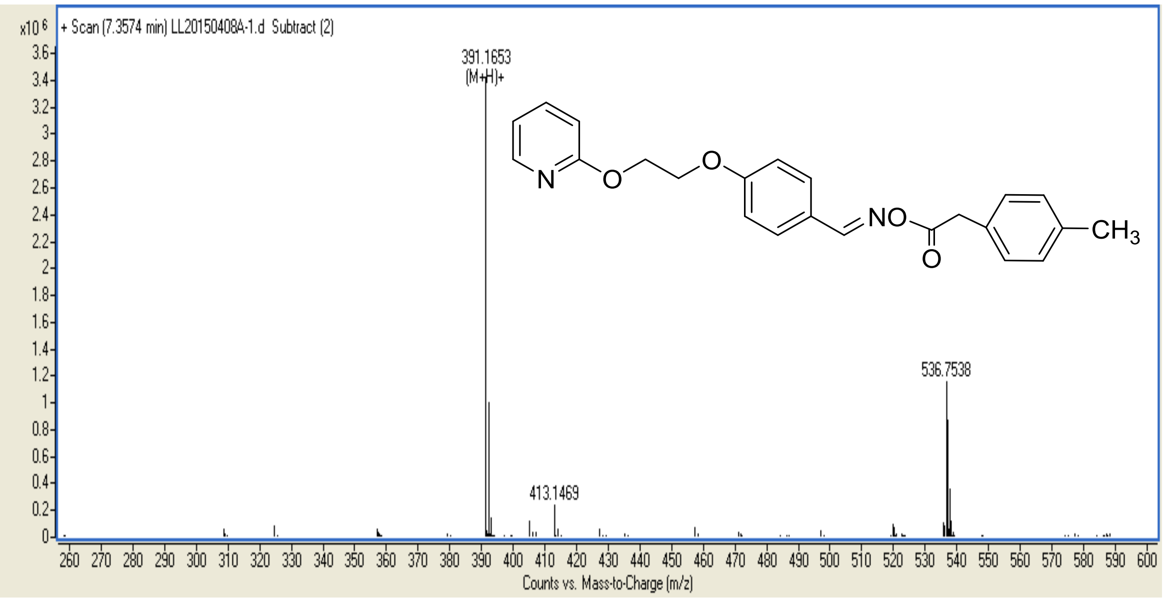

^{1}H-NMR spectrum of compound **5f.**

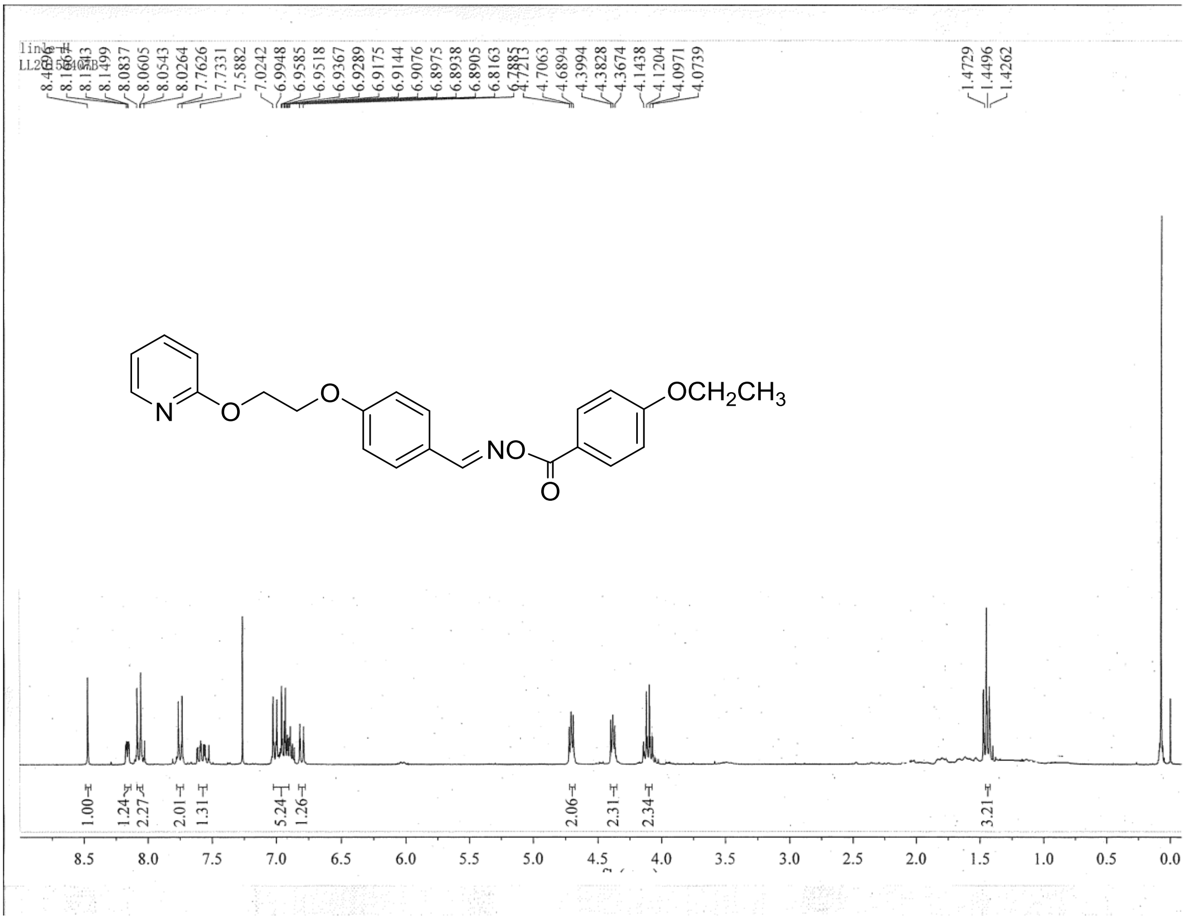

^{13}C-NMR spectrum of compound **5f.**

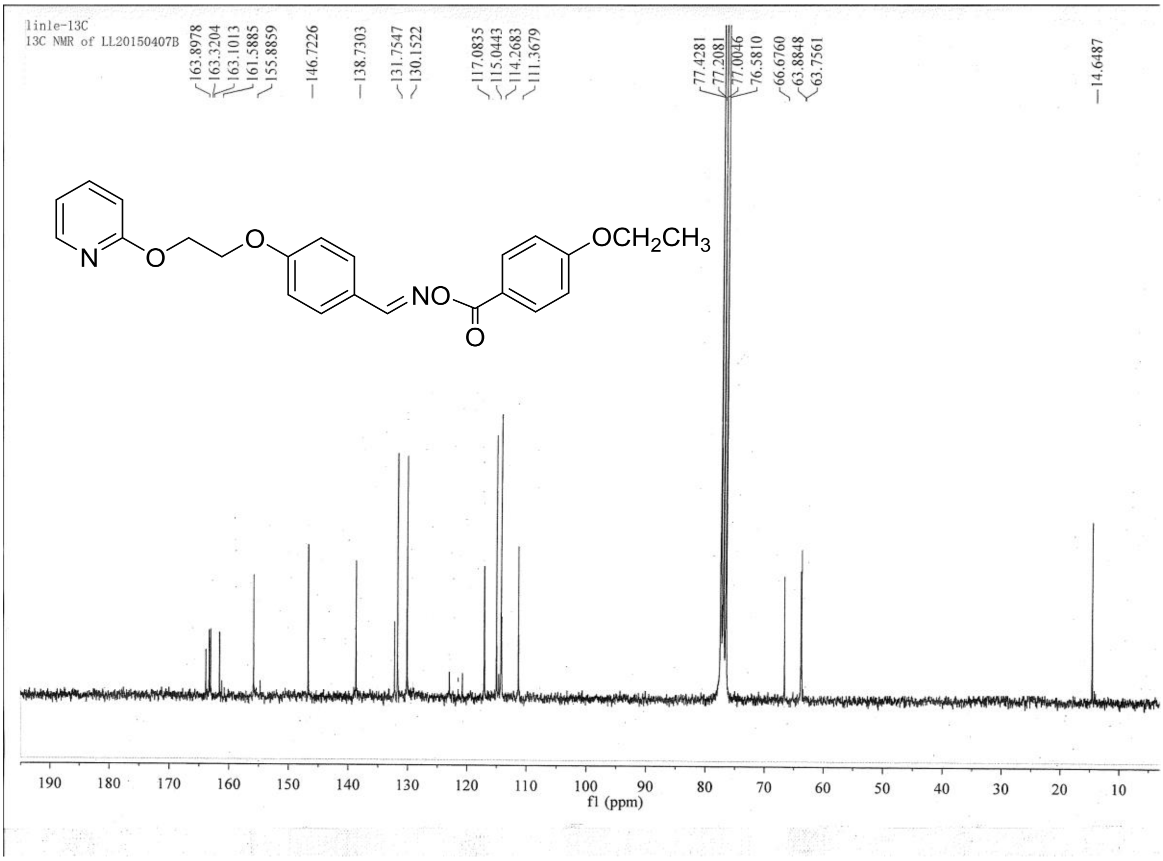

HRMS of compound **5f.**

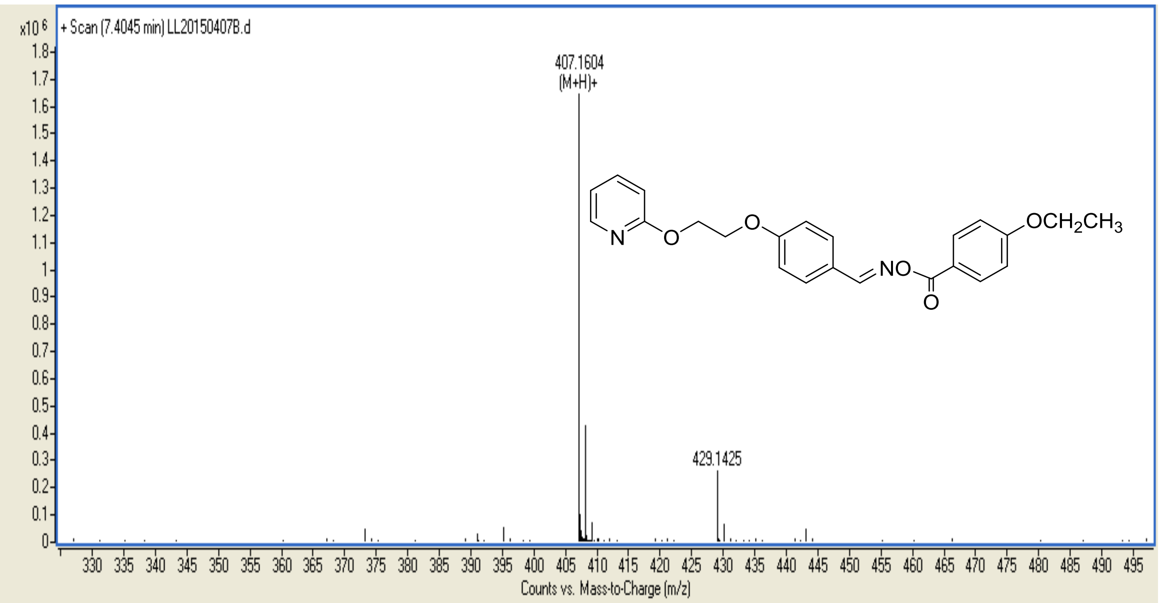

^{1}H-NMR spectrum of compound **5g.**

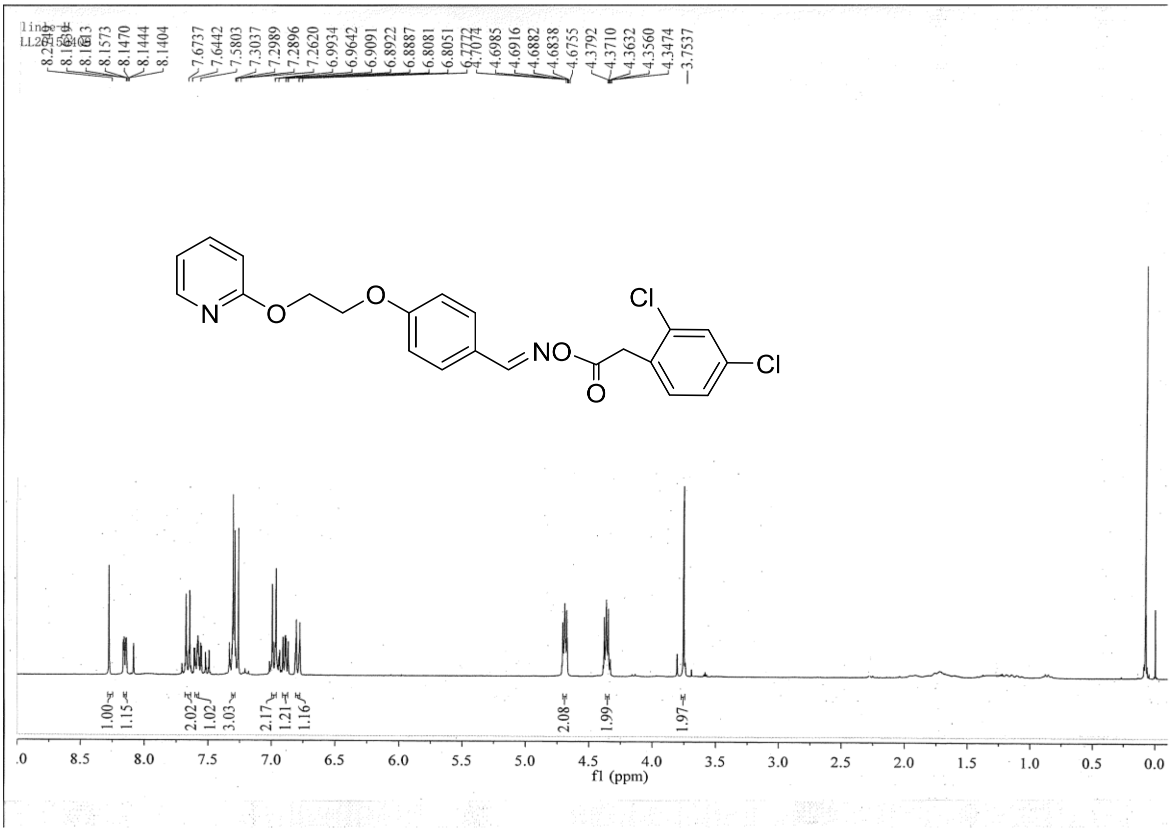

^{13}C-NMR spectrum of compound **5g.**

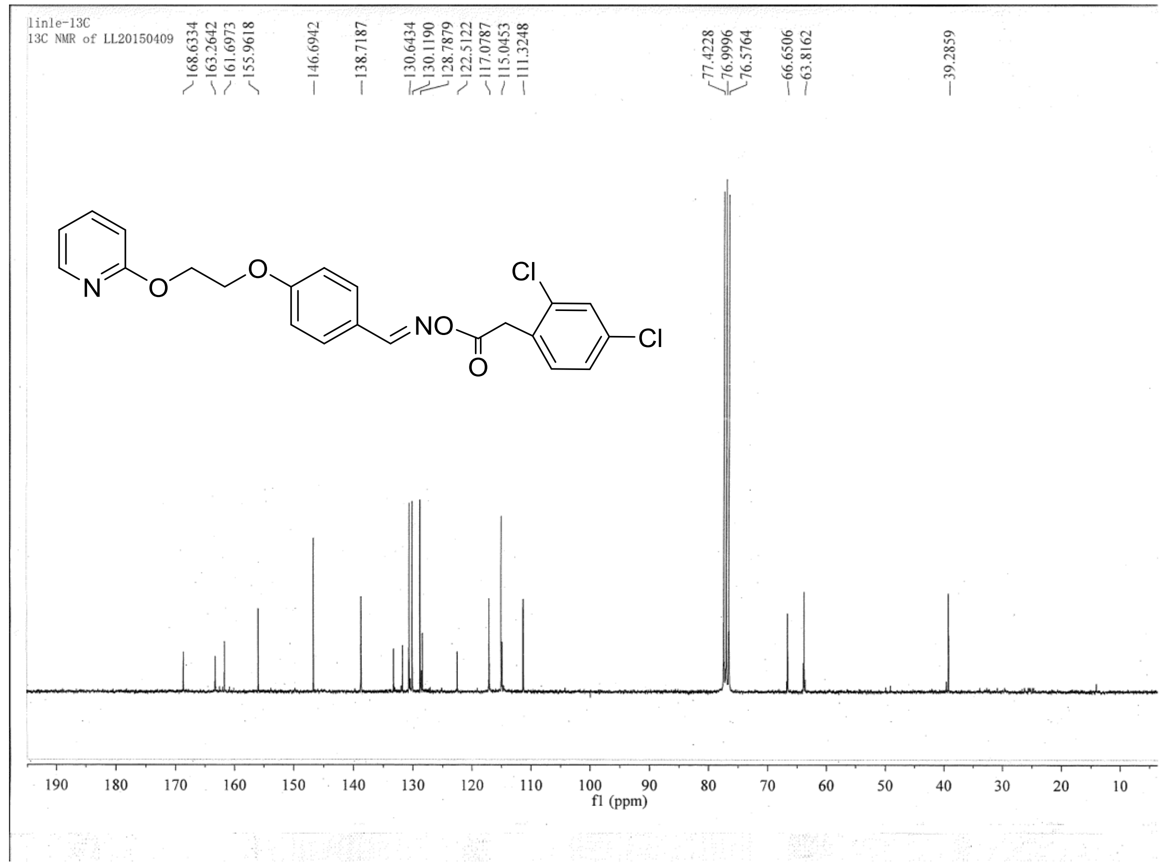

HRMS of compound **5g.**

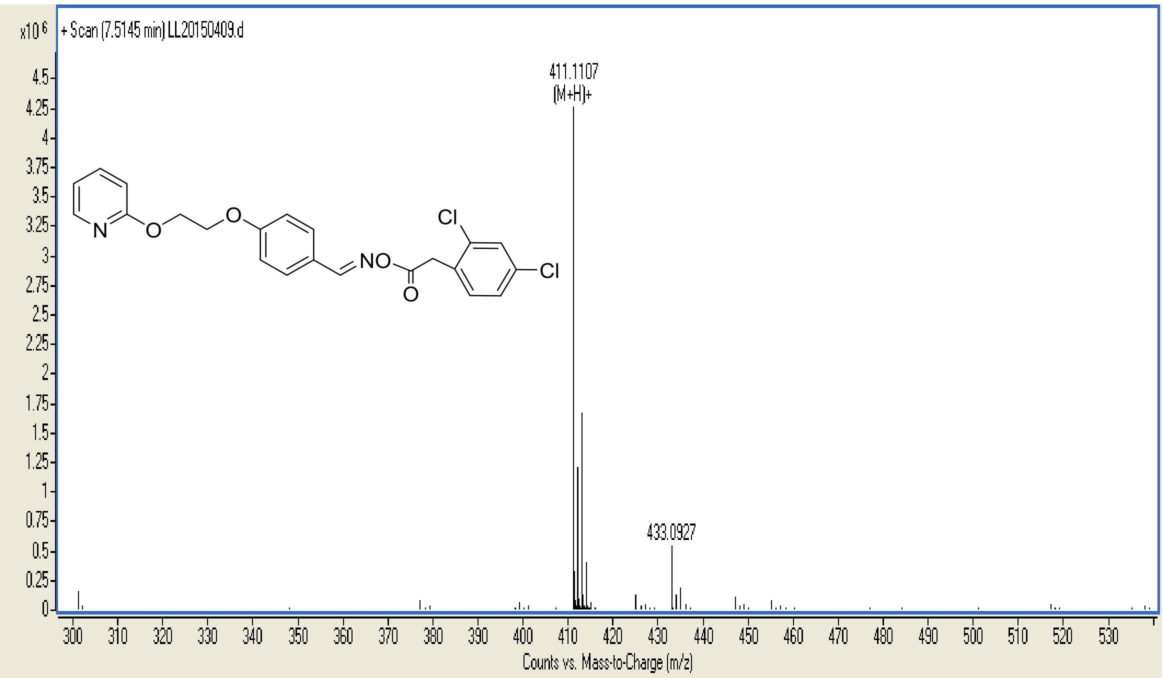

^{1}H-NMR spectrum of compound **5h.**

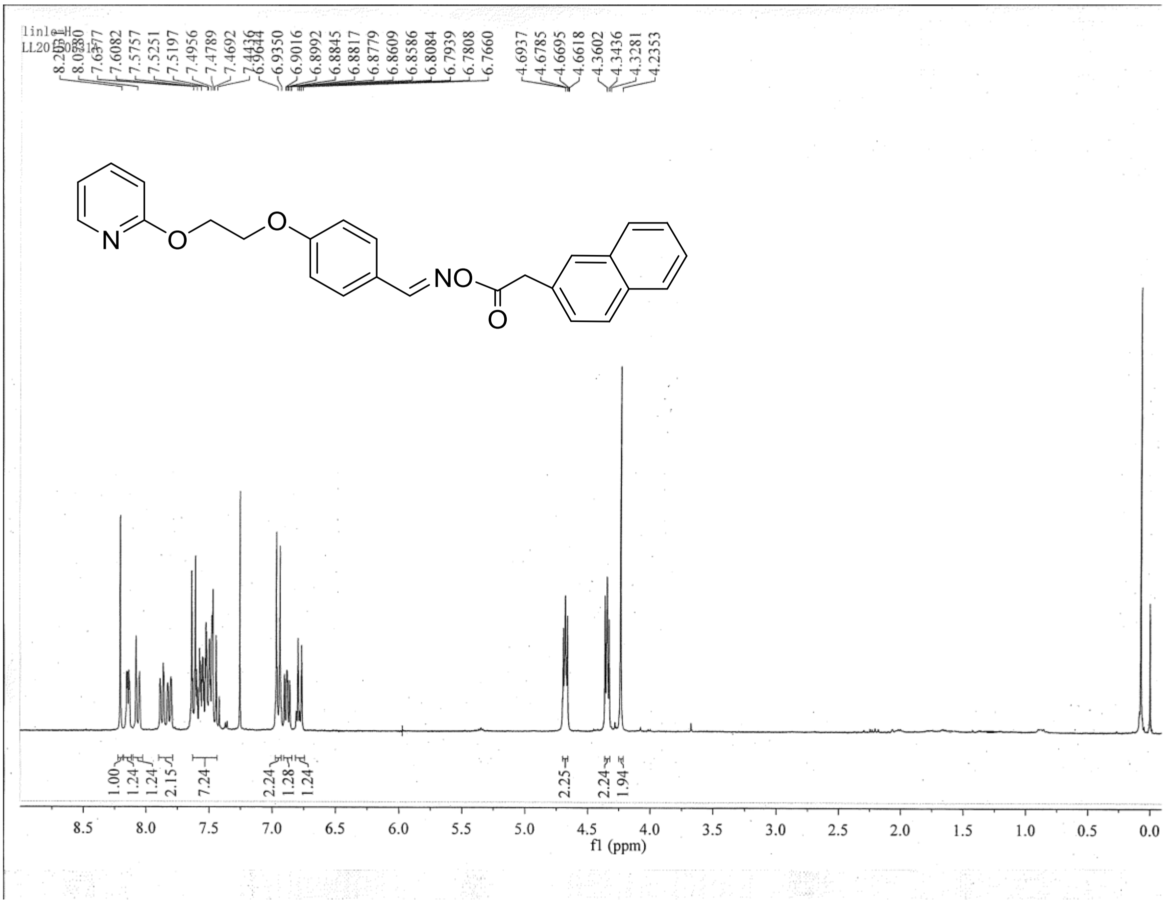

^{13}C-NMR spectrum of compound **5h.**

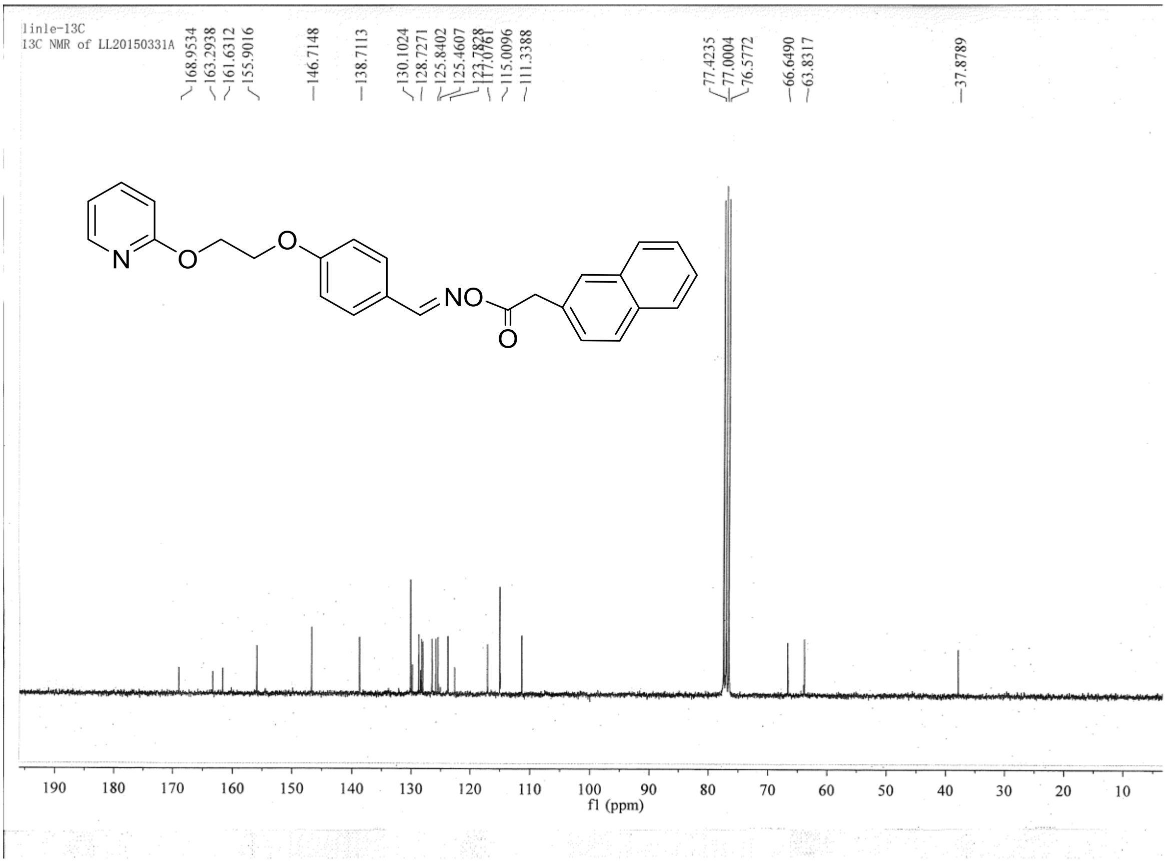

HRMS of compound **5h.**

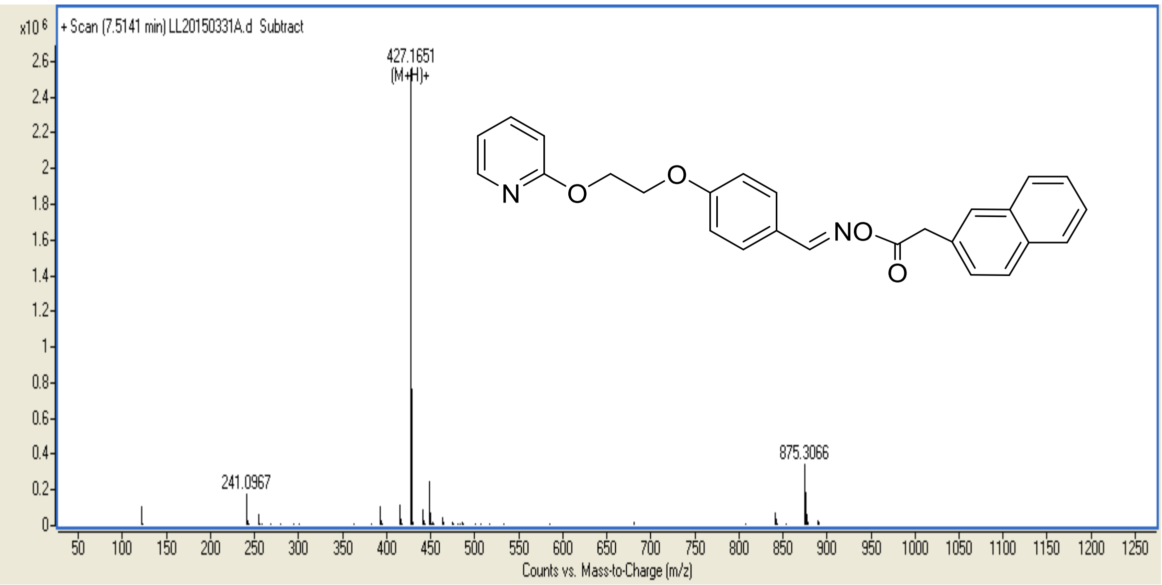

^{1}H-NMR spectrum of compound **5i.**

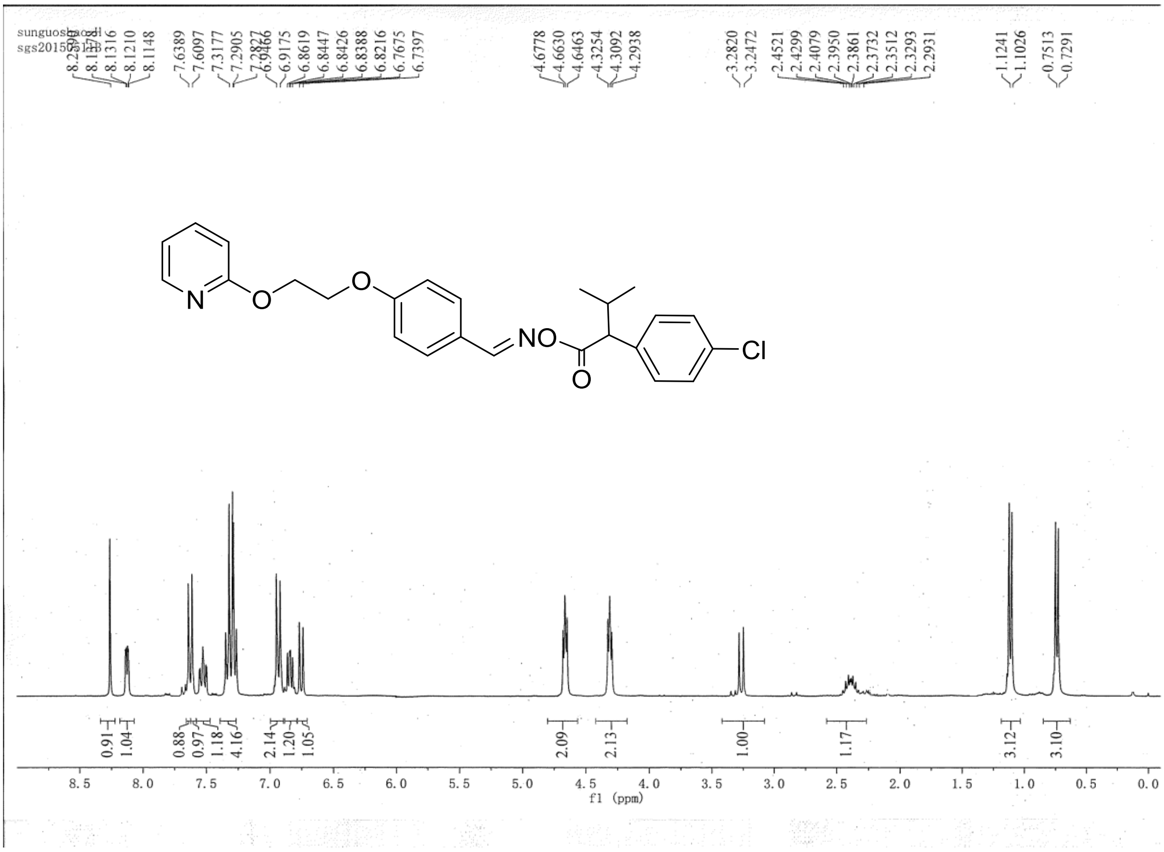

^{13}C-NMR spectrum of compound **5i.**

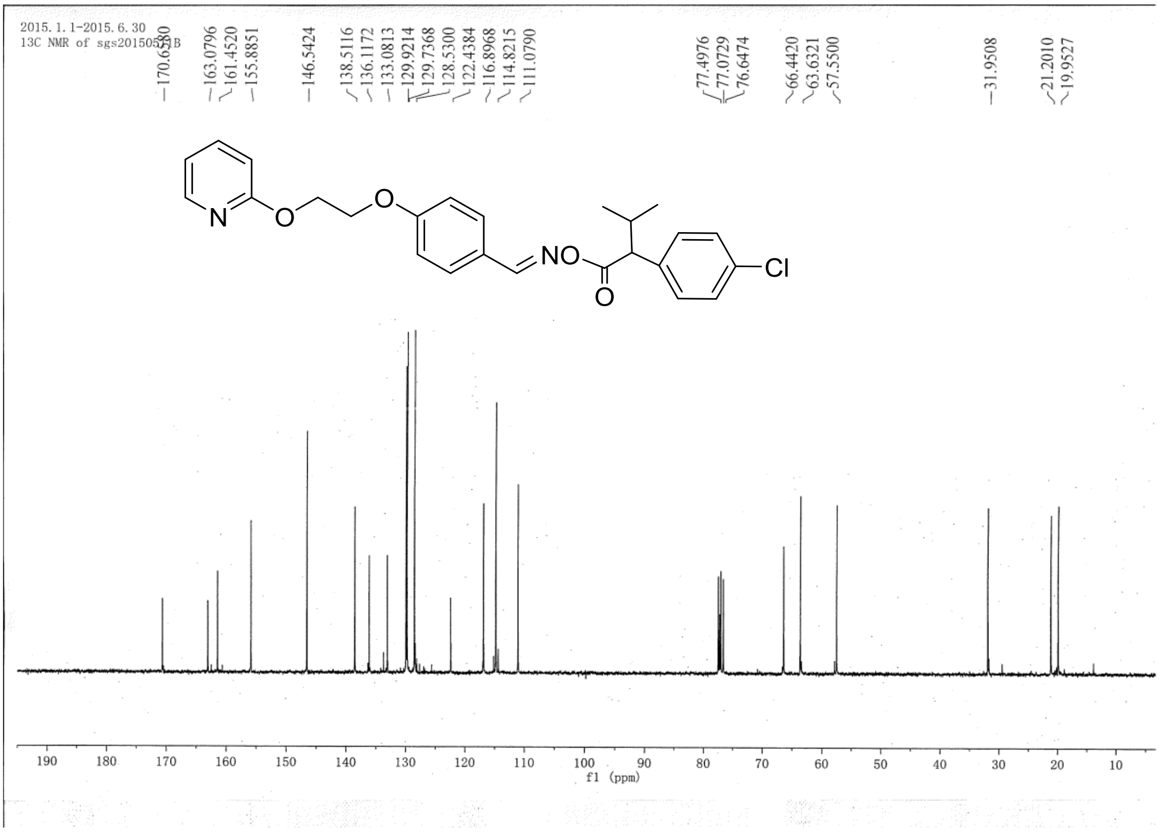

HRMS of compound **5i.**

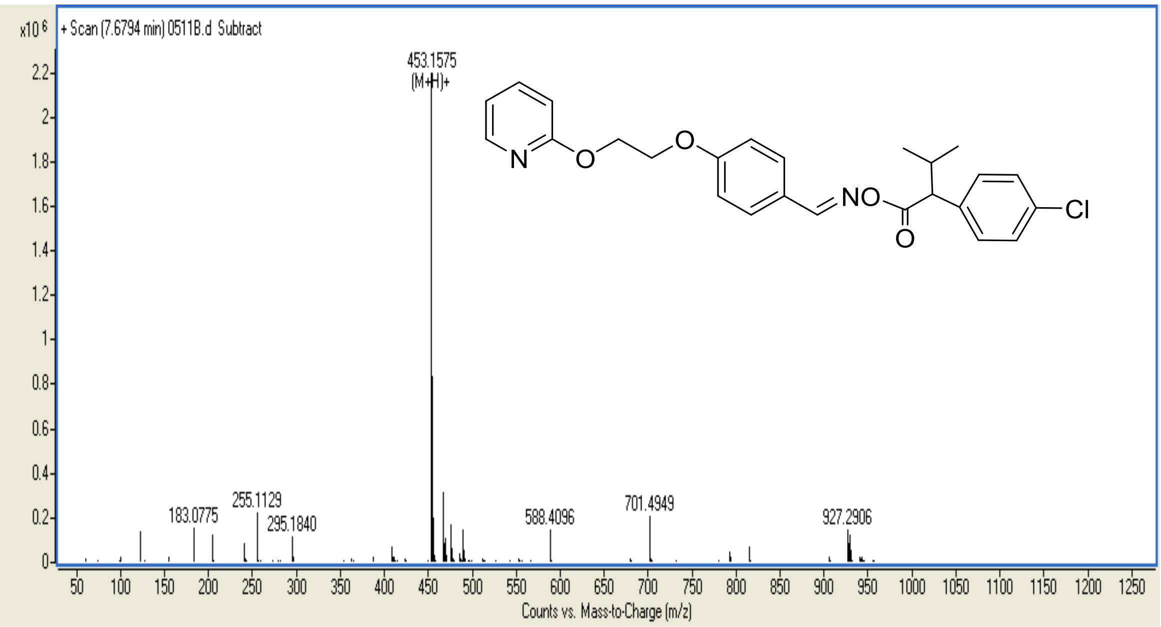

¹H-NMR spectrum of compound **5j.**

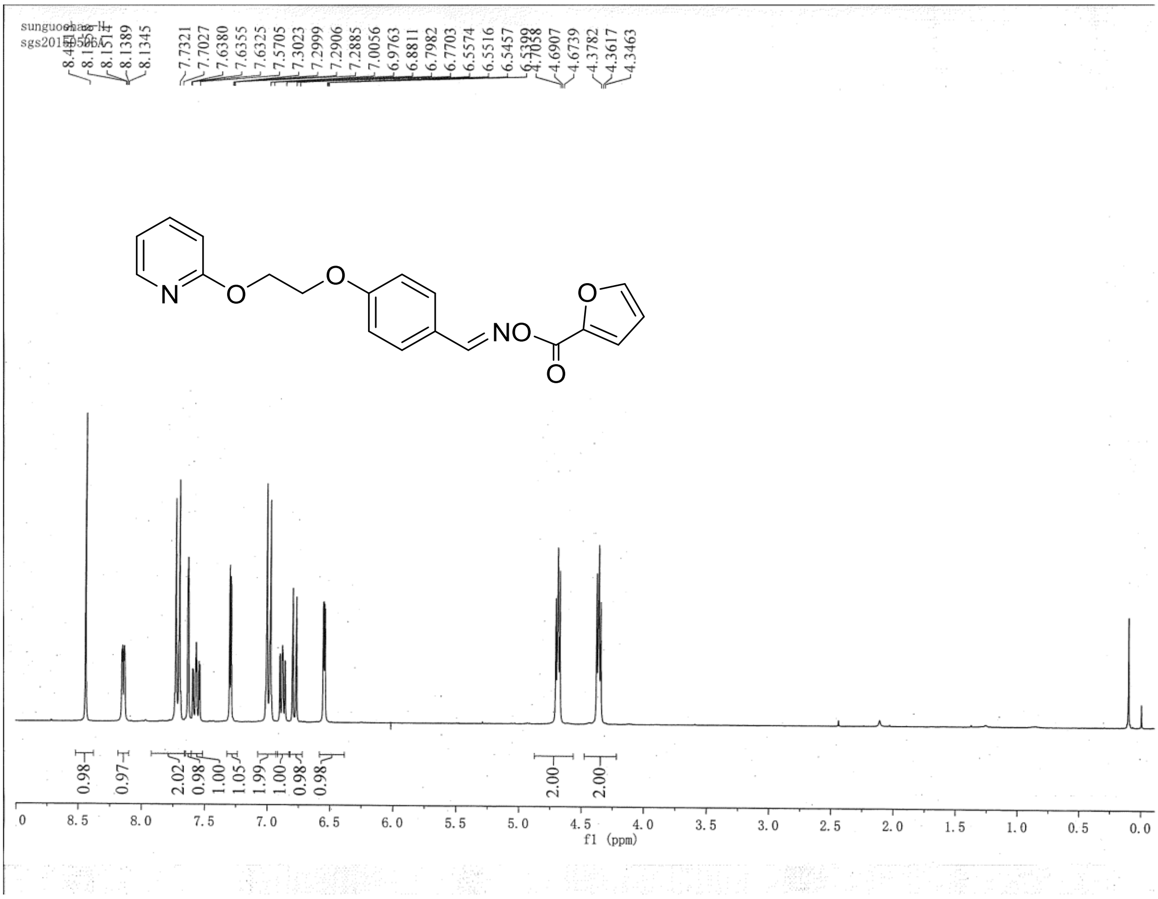

^{13}C-NMR spectrum of compound **5j.**

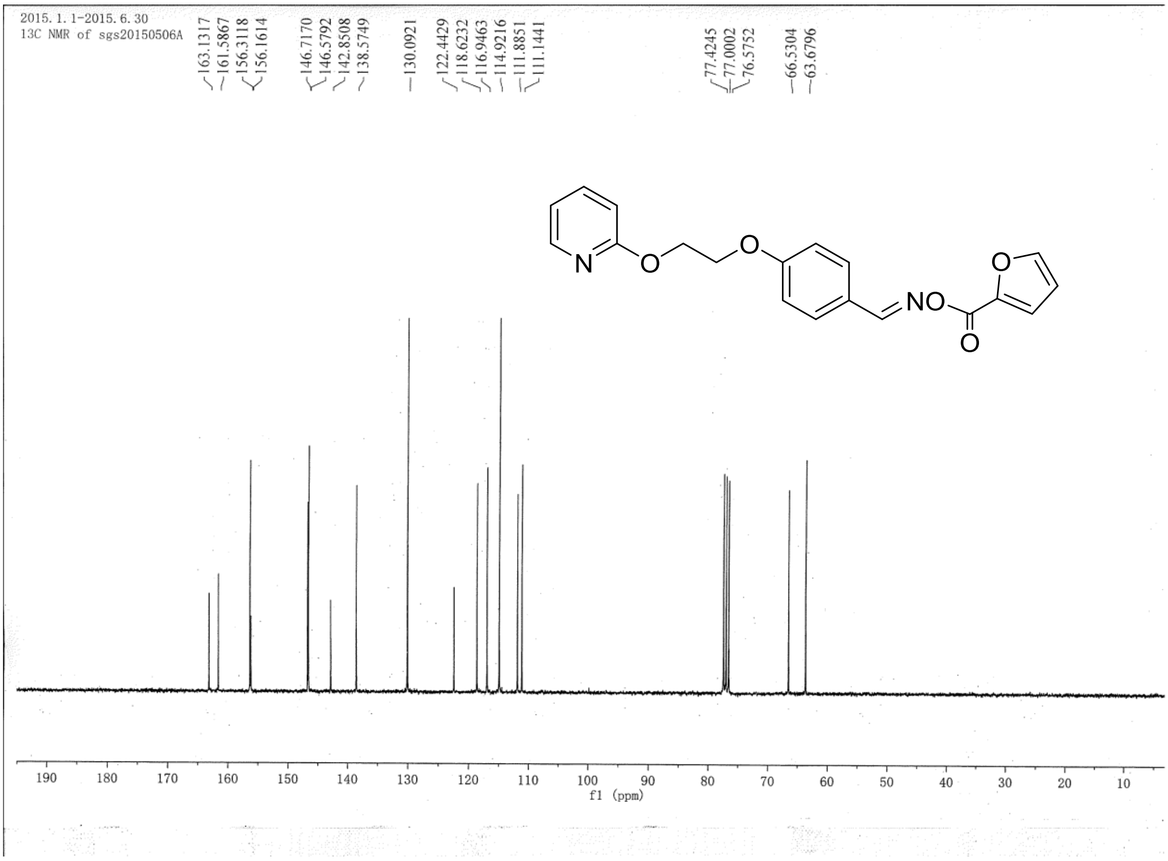

HRMS of compound **5j.**

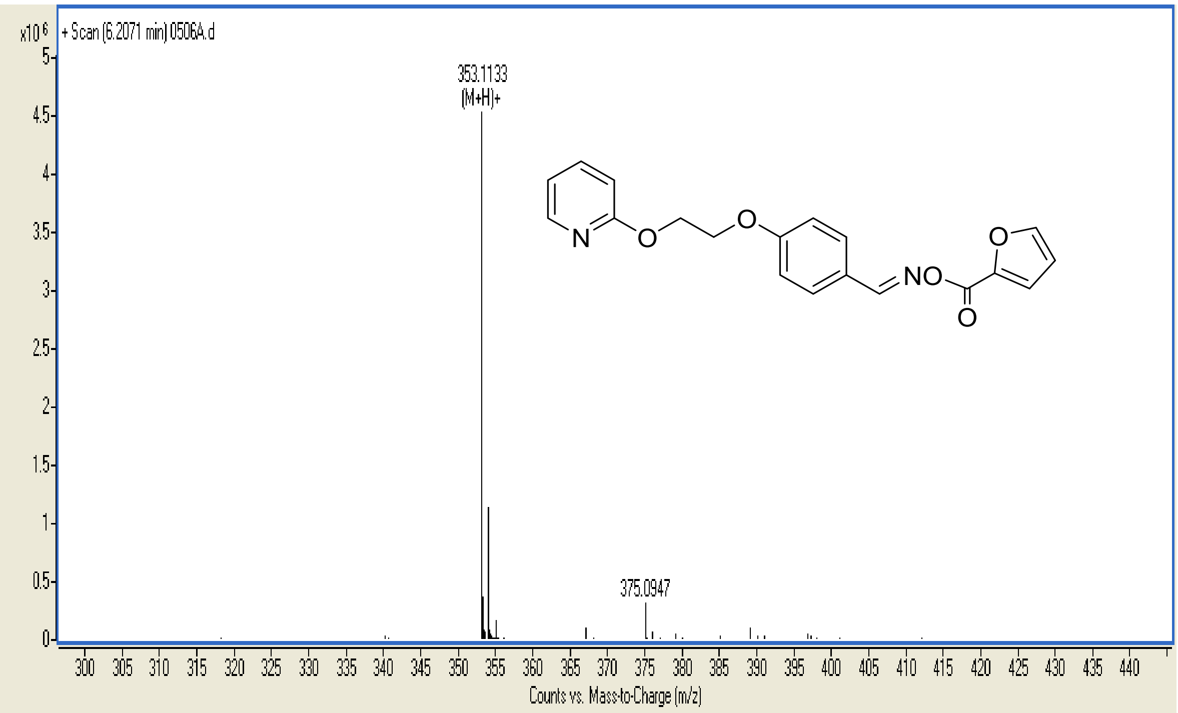

^{1}H-NMR spectrum of compound **5k.**

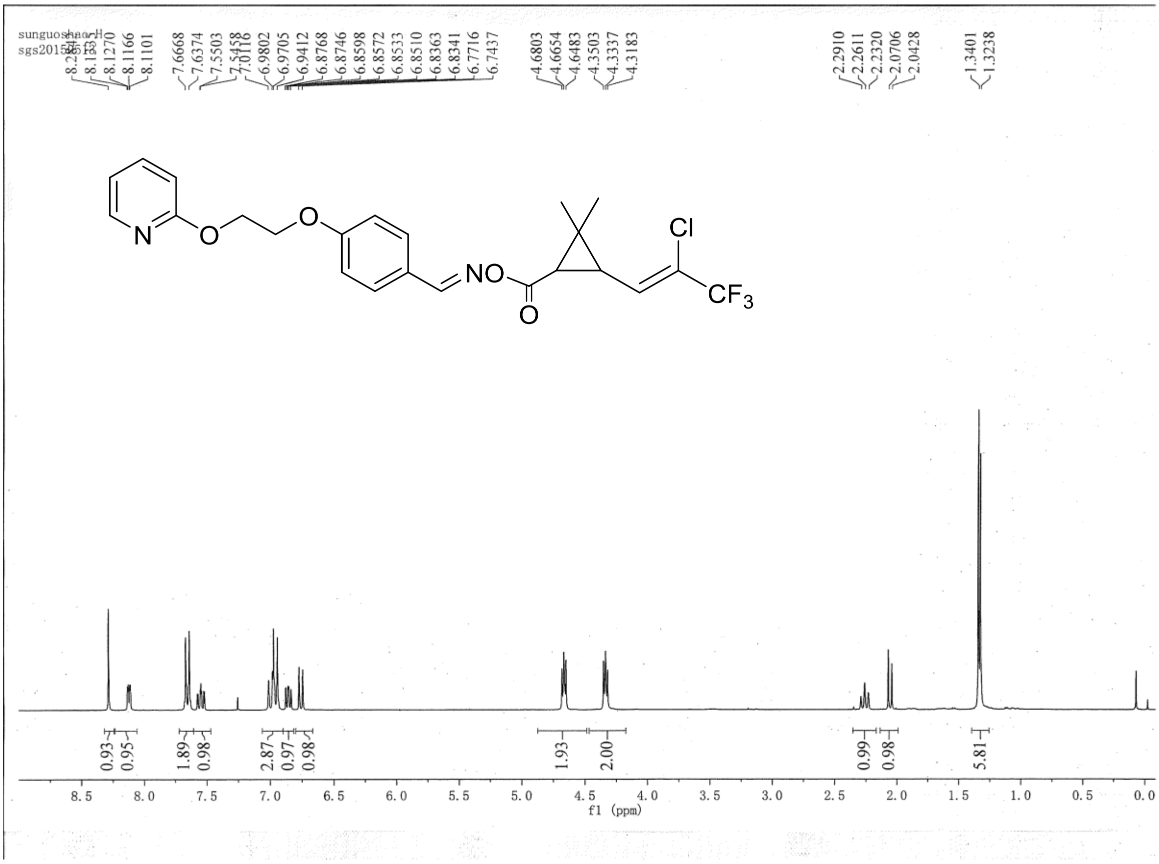

^{13}C-NMR spectrum of compound **5k.**

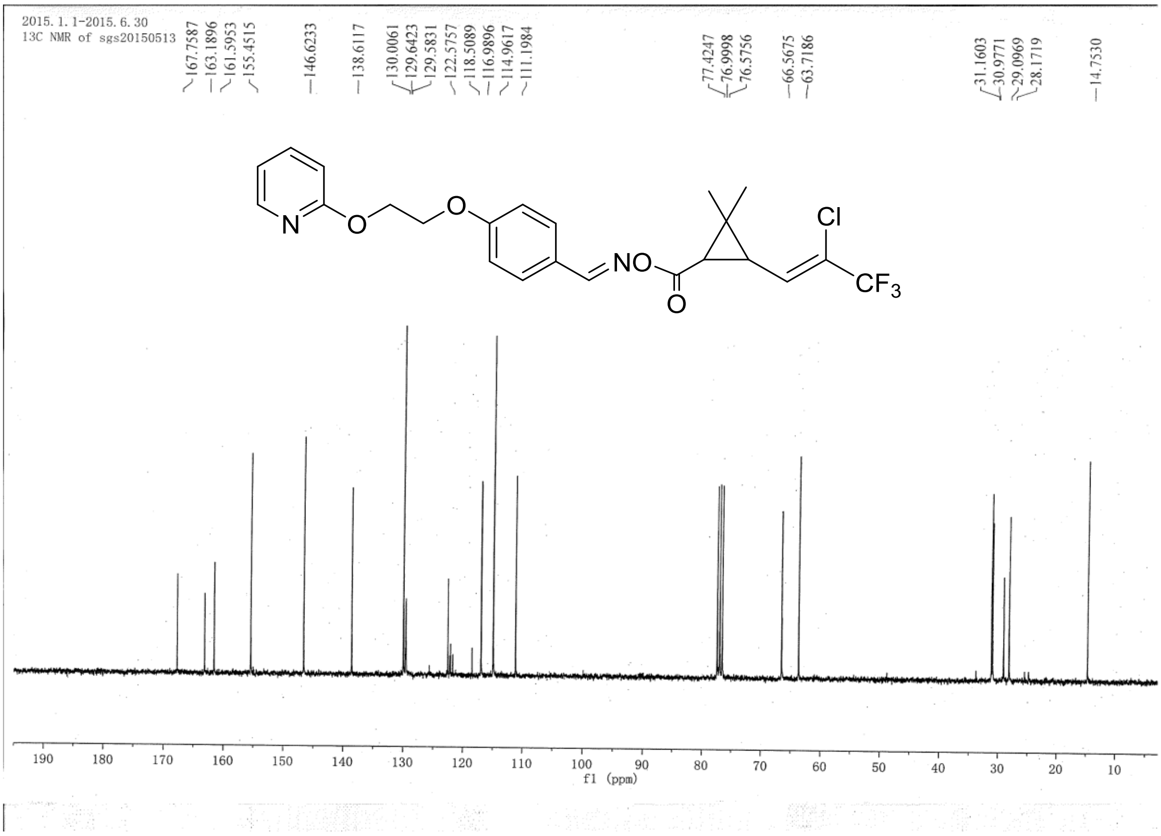

HRMS of compound **5k.**

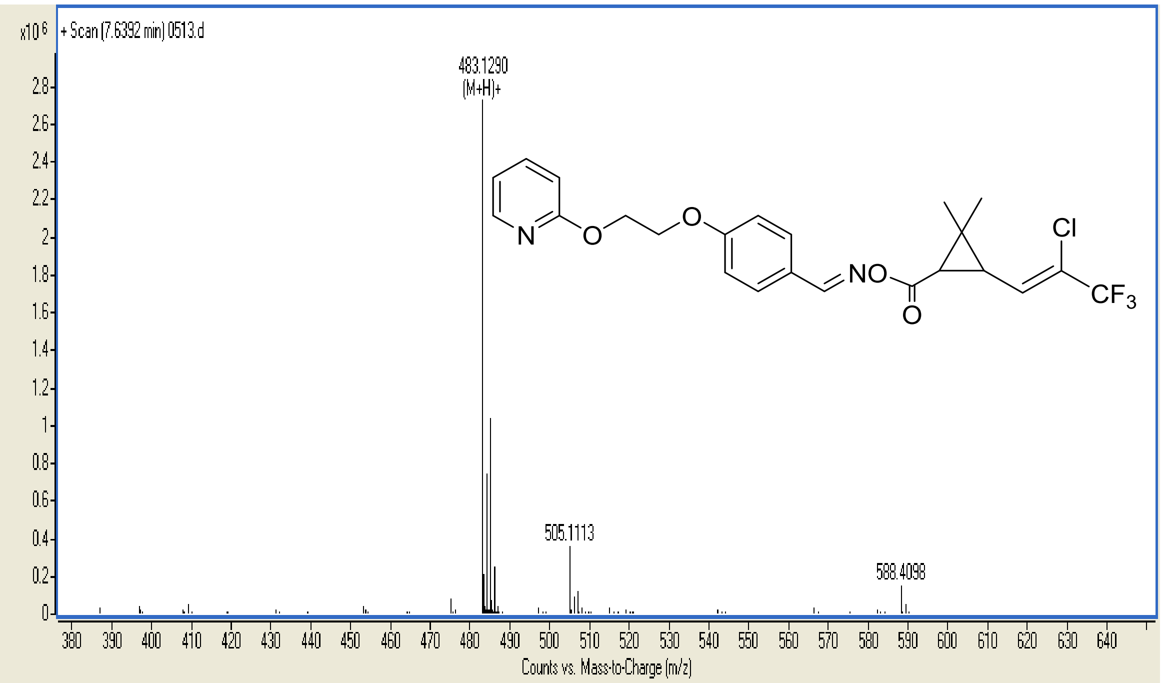

^{1}H-NMR spectrum of compound **5l.**

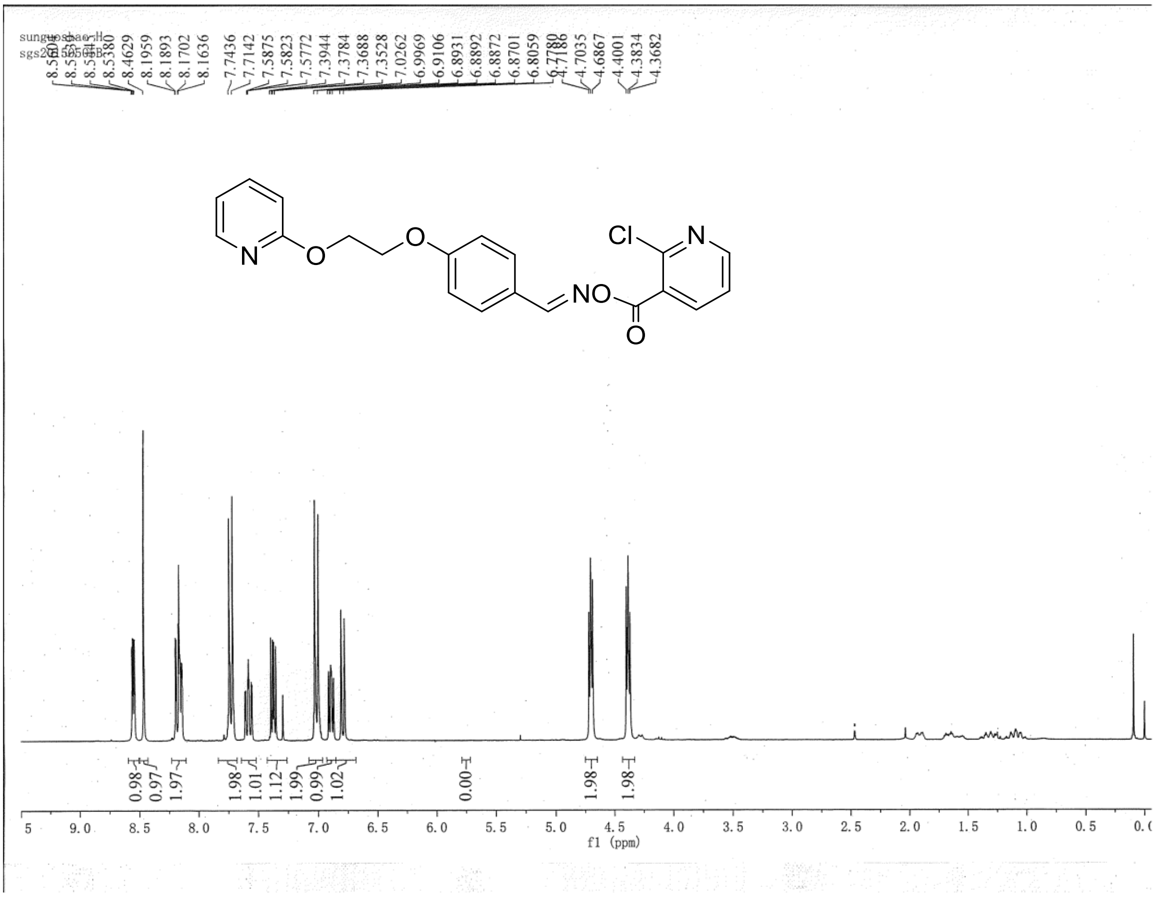

^{13}C-NMR spectrum of compound **5l.**

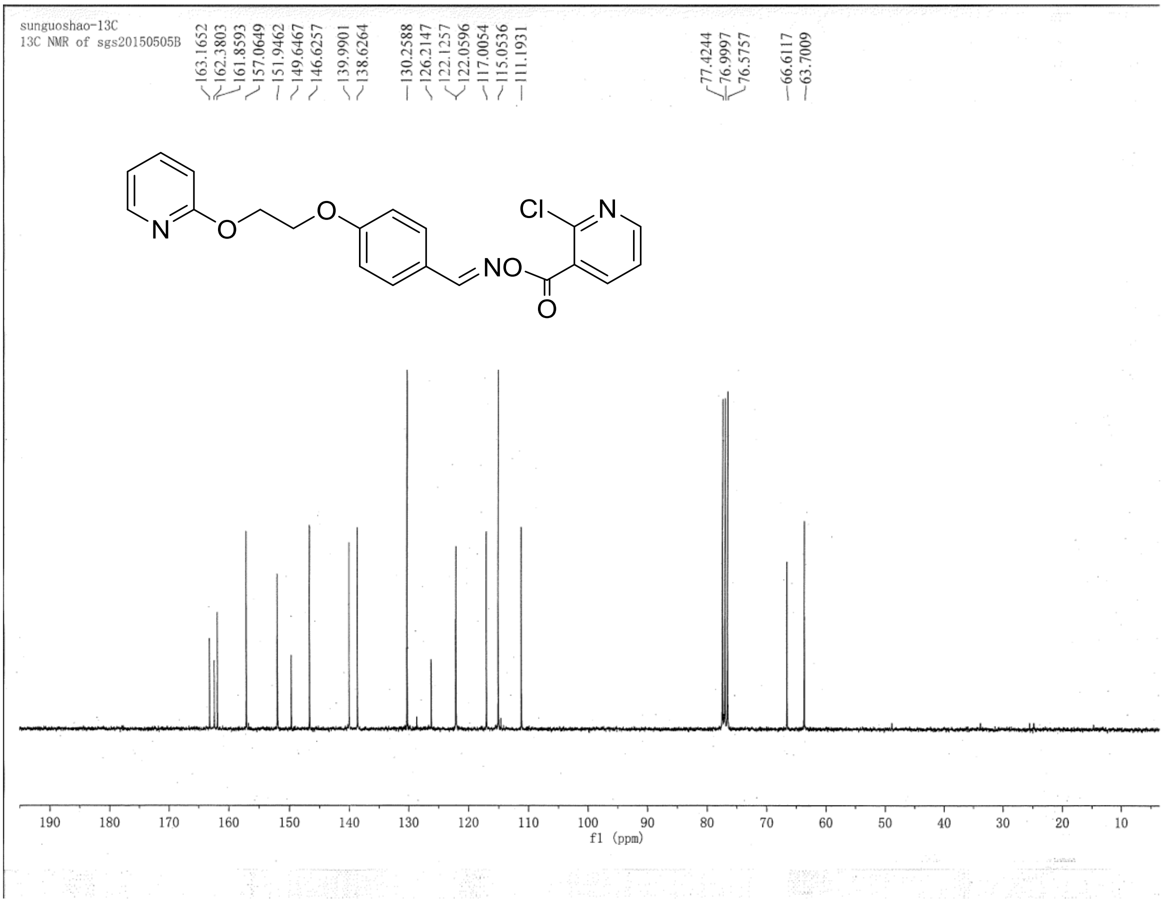

HRMS of compound **5l.**

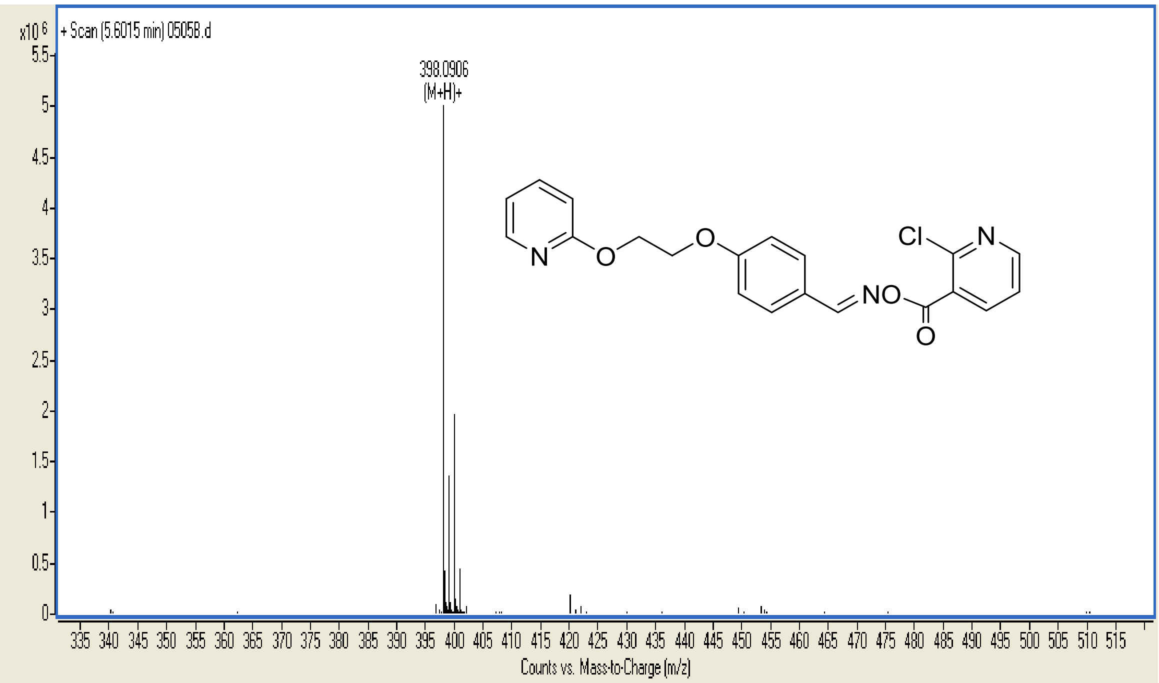

^{1}H-NMR spectrum of compound **5m.**

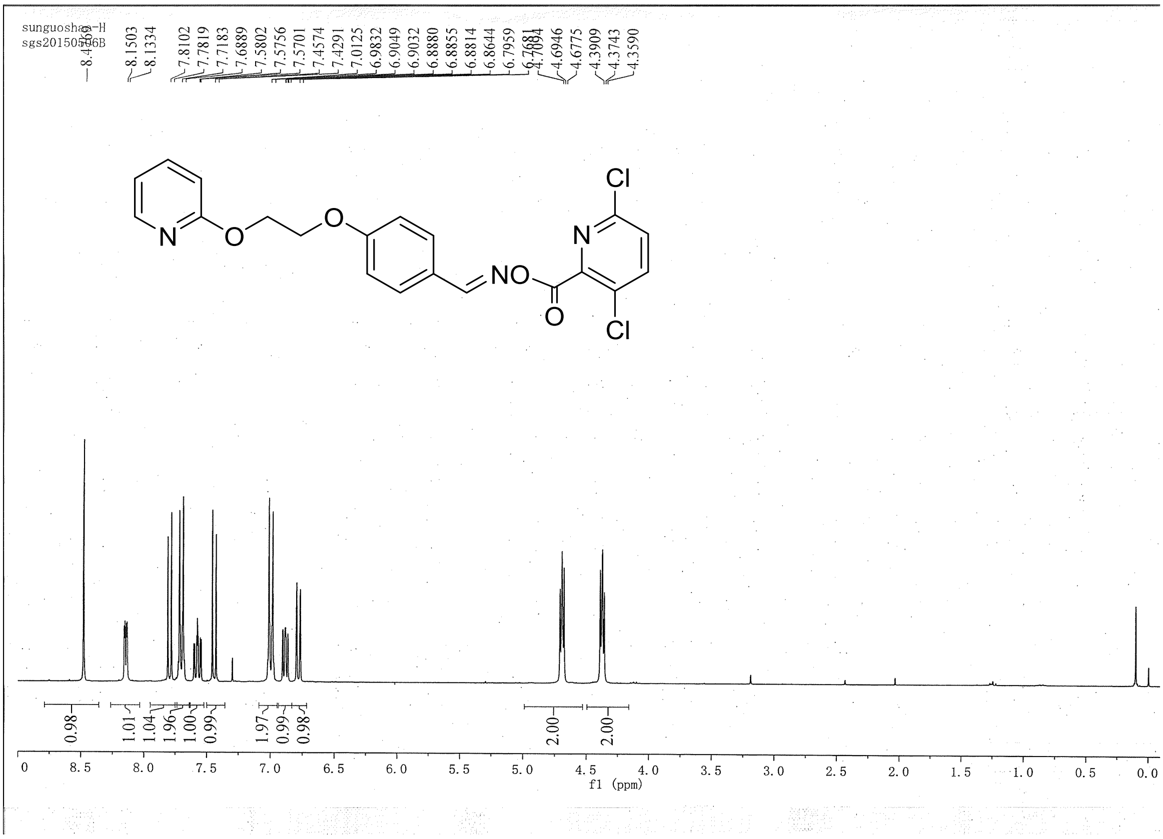

^{13}C-NMR spectrum of compound **5m.**

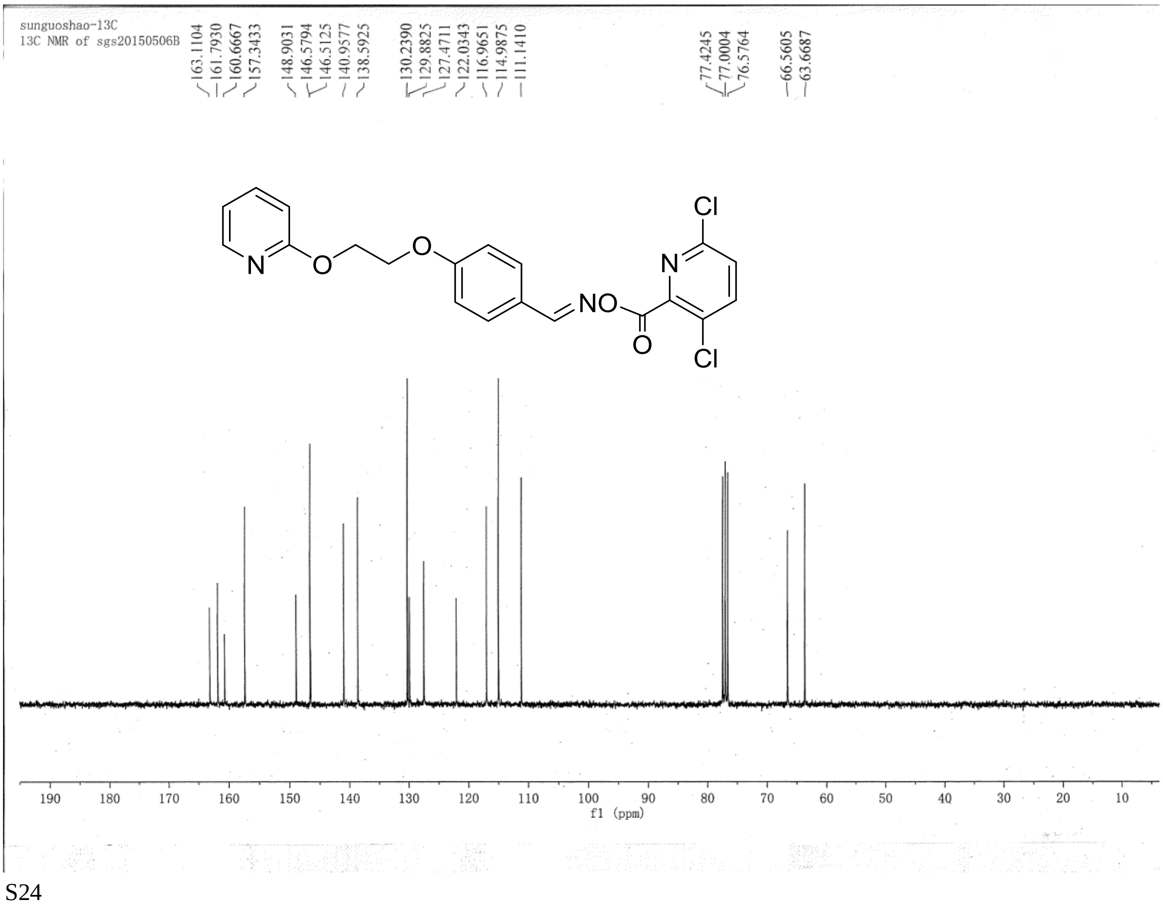

HRMS of compound **5m.**

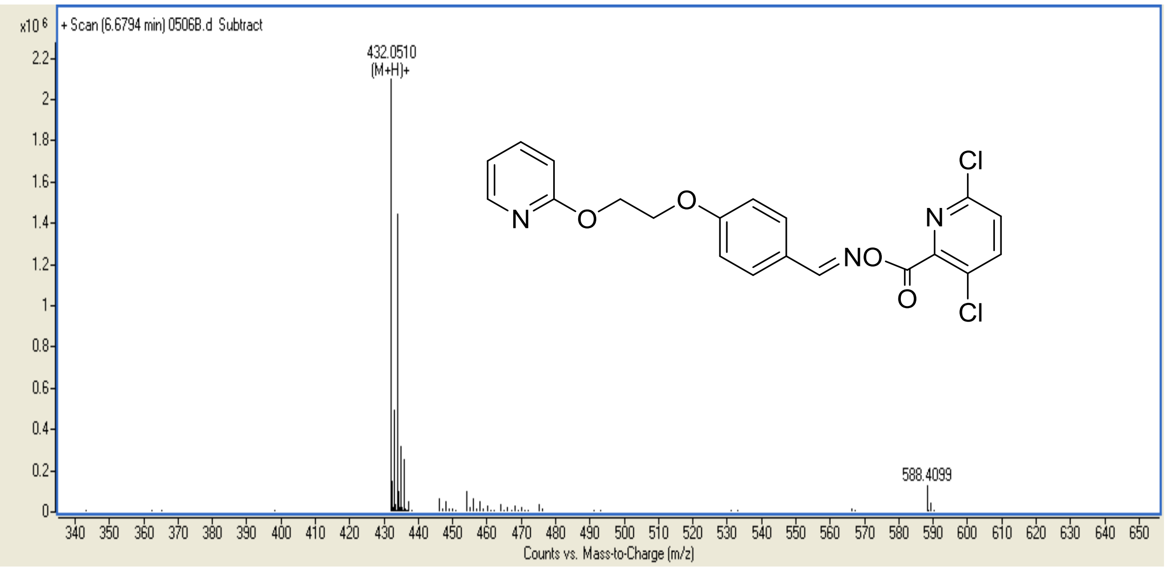

^{1}H-NMR spectrum of compound **5n.**

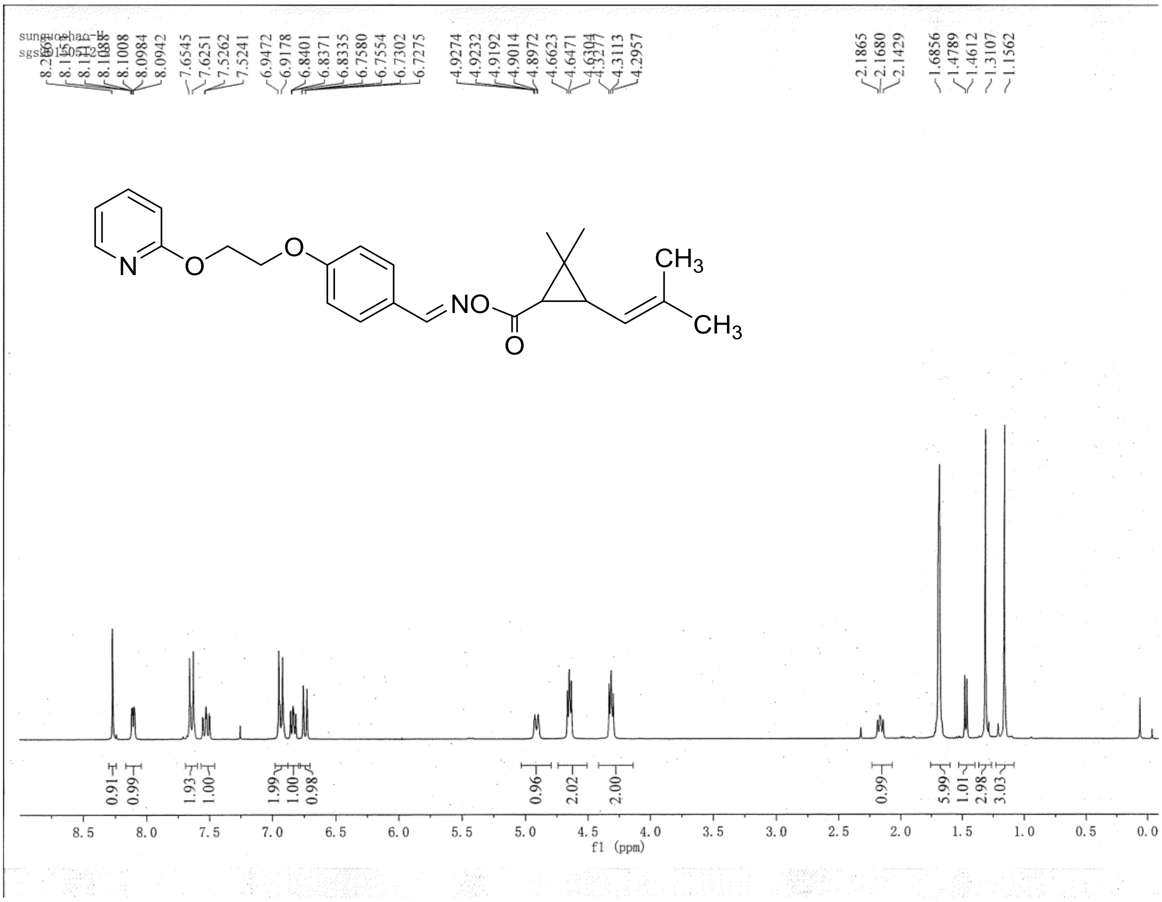

^{13}C-NMR spectrum of compound **5n.**

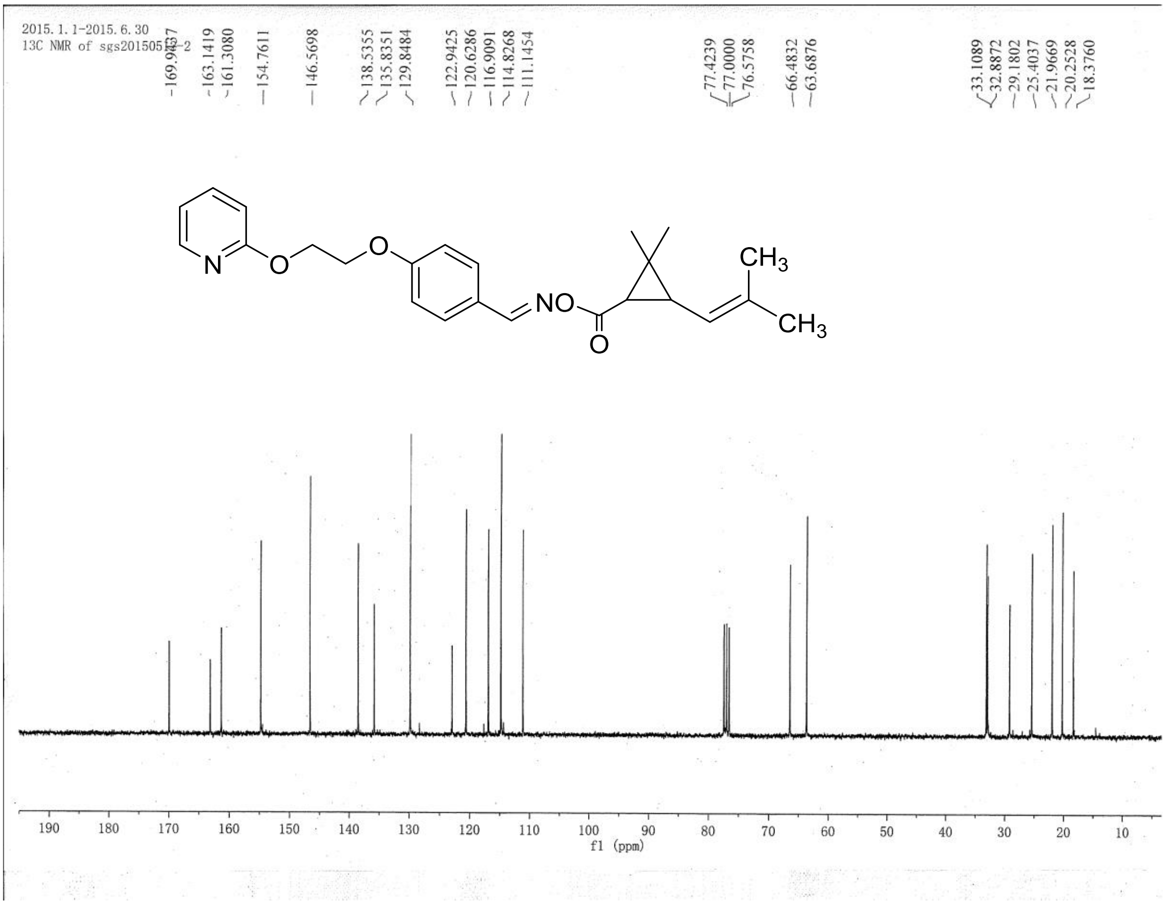

HRMS of compound **5n.**

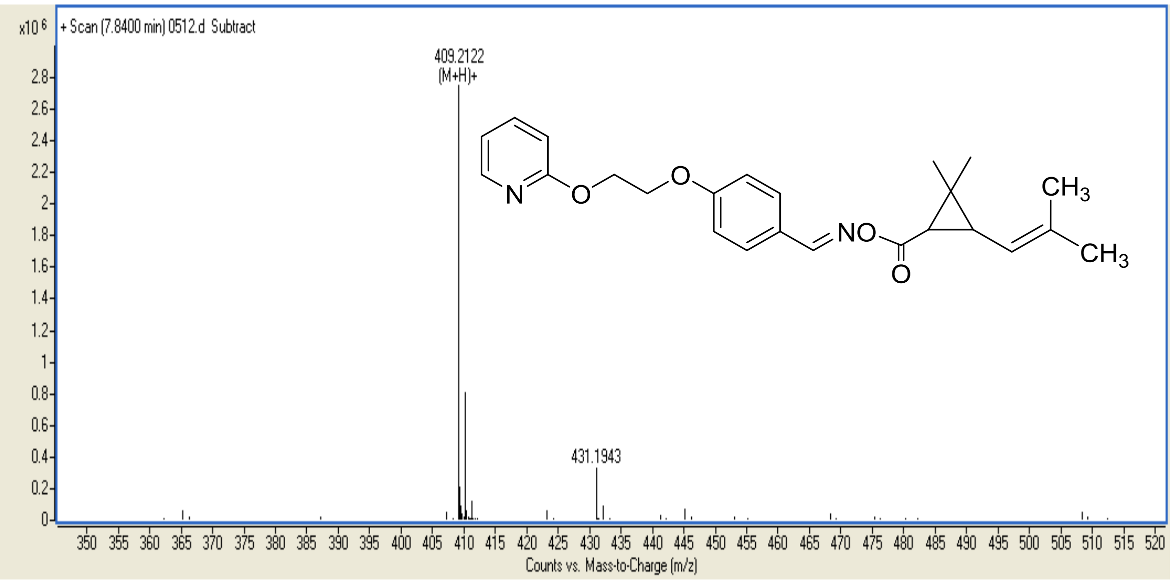

^{1}H-NMR spectrum of compound **5o.**

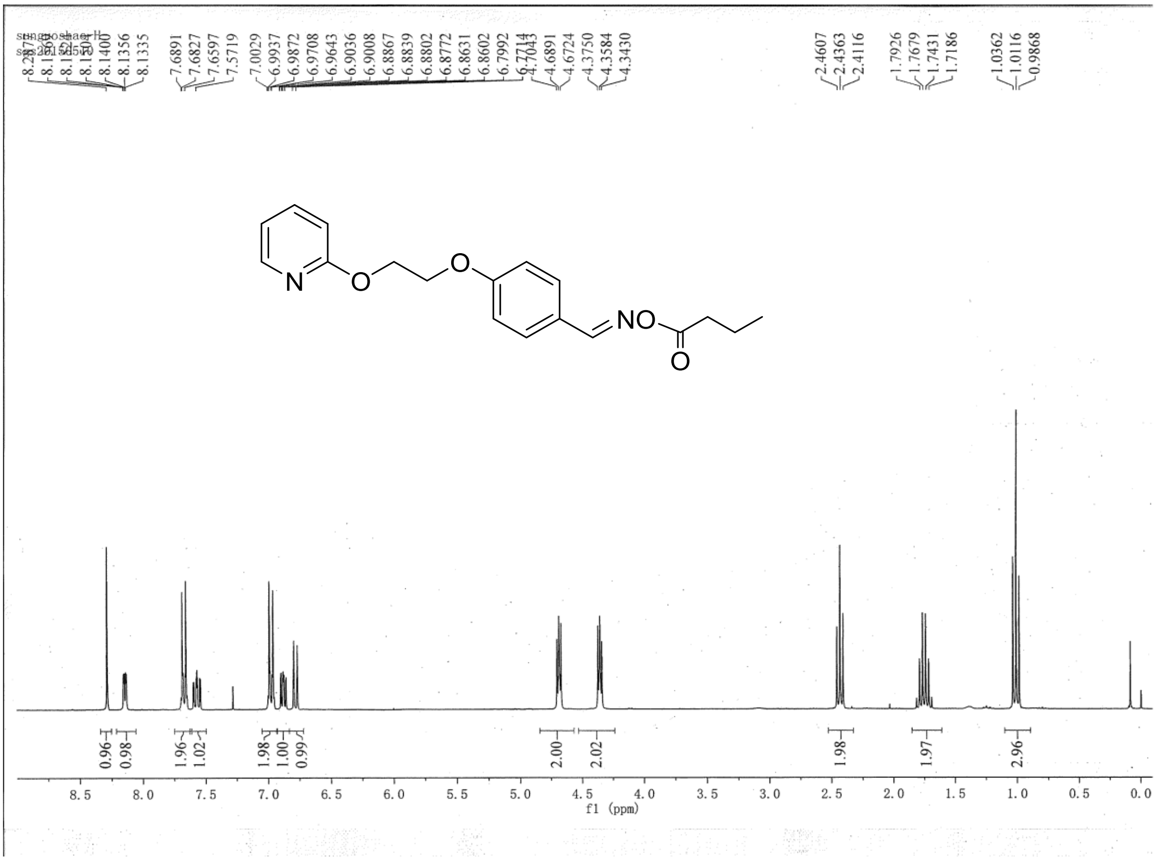

^{13}C-NMR spectrum of compound **5o.**

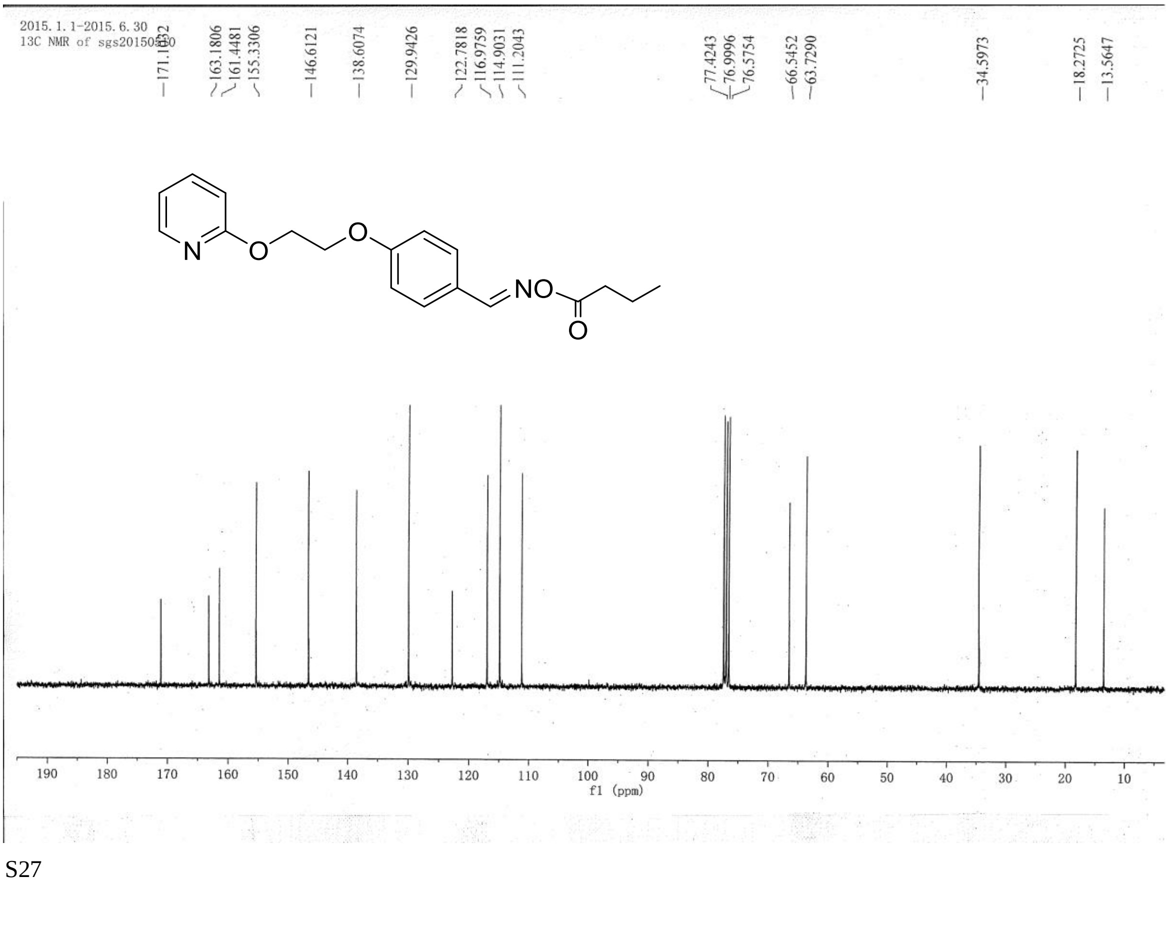

HRMS of compound **5o.**

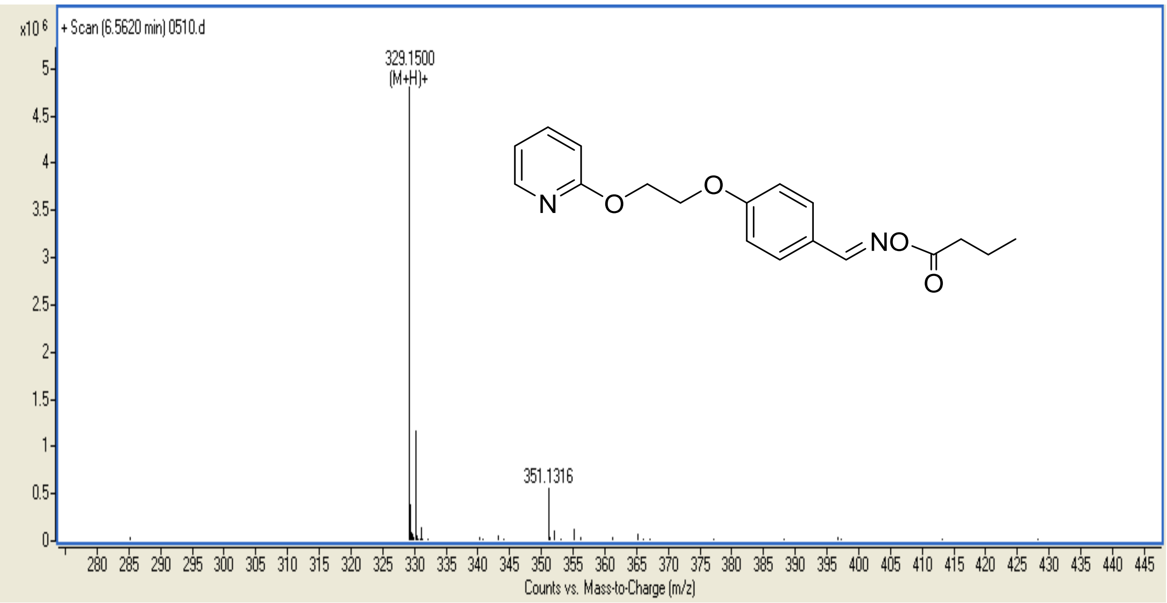

^{1}H-NMR spectrum of compound **5p.**

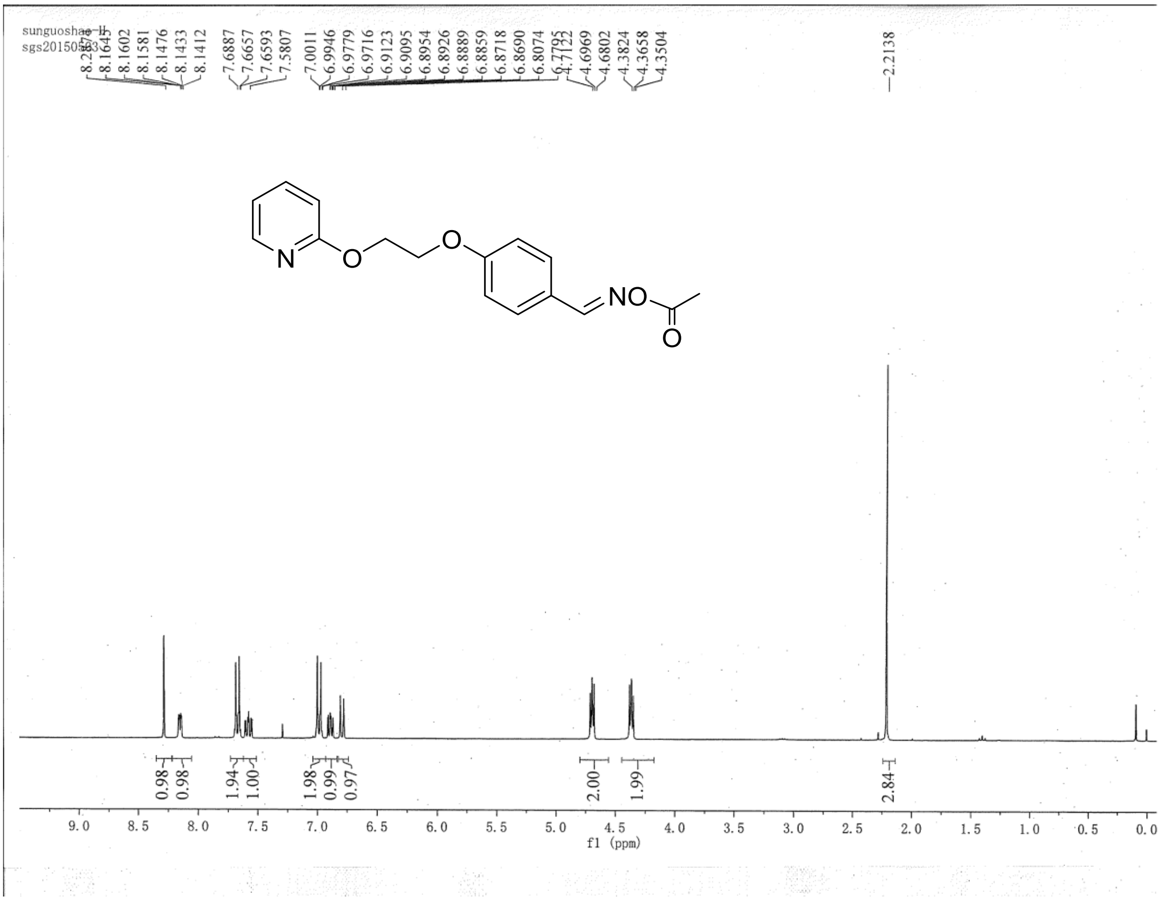

^{13}C-NMR spectrum of compound **5p.**

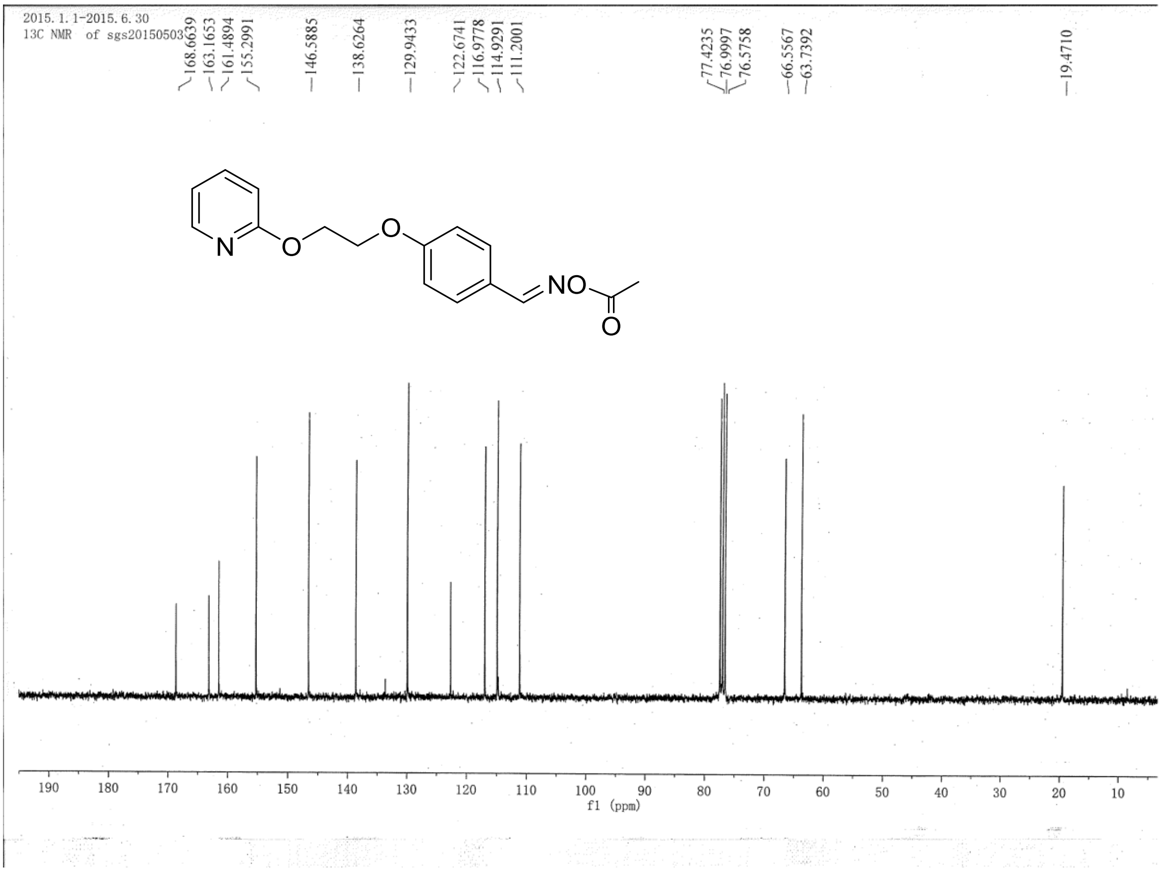

HRMS of compound **5p.**

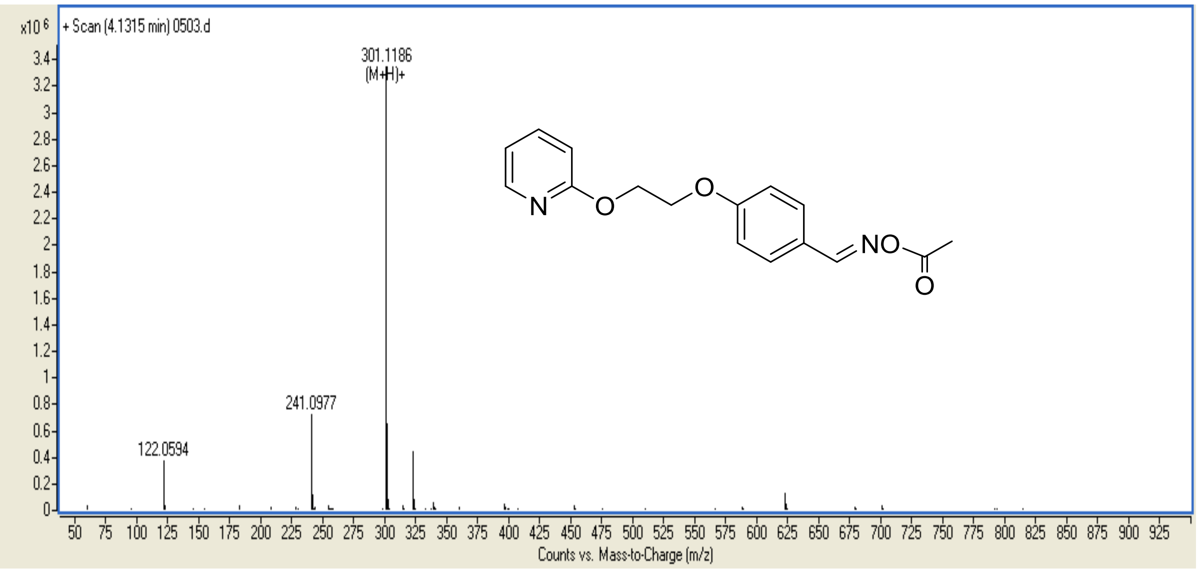

^{1}H-NMR spectrum of compound **5q.**

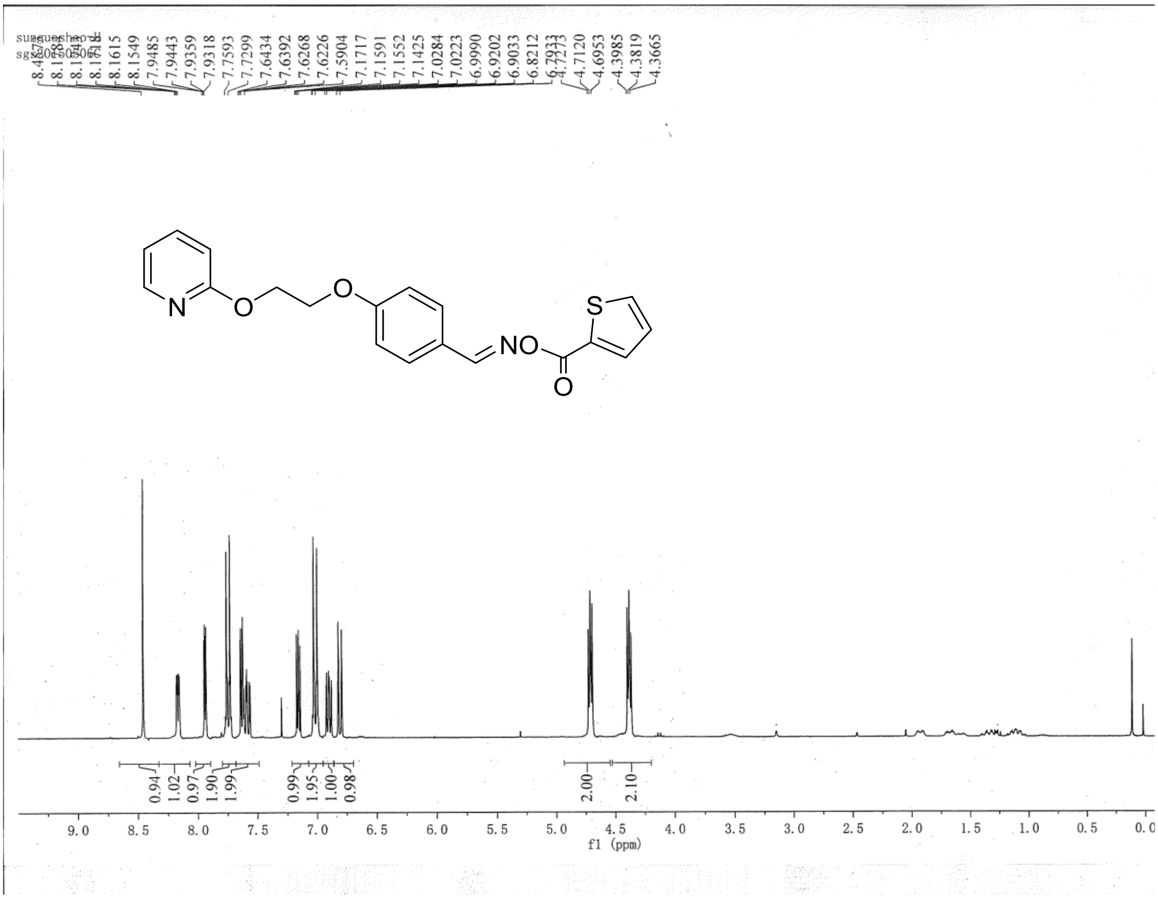

^{13}C-NMR spectrum of compound **5q.**

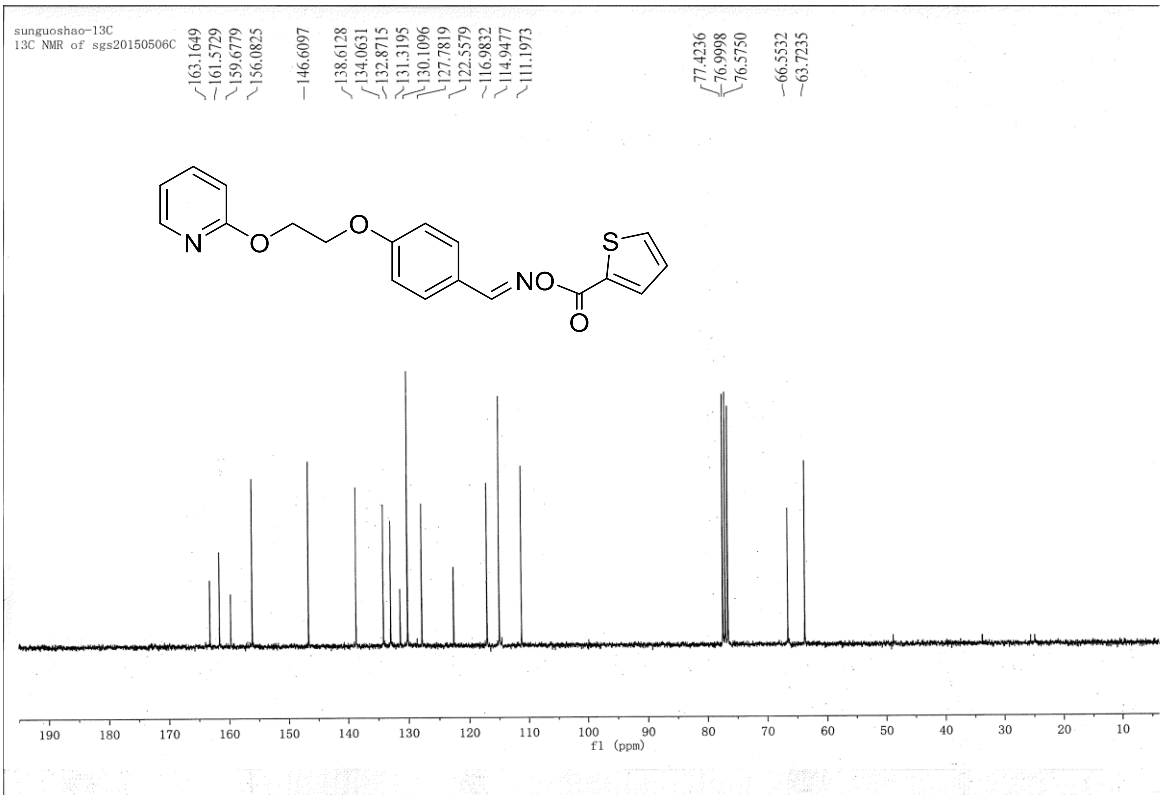

HRMS of compound **5q.**

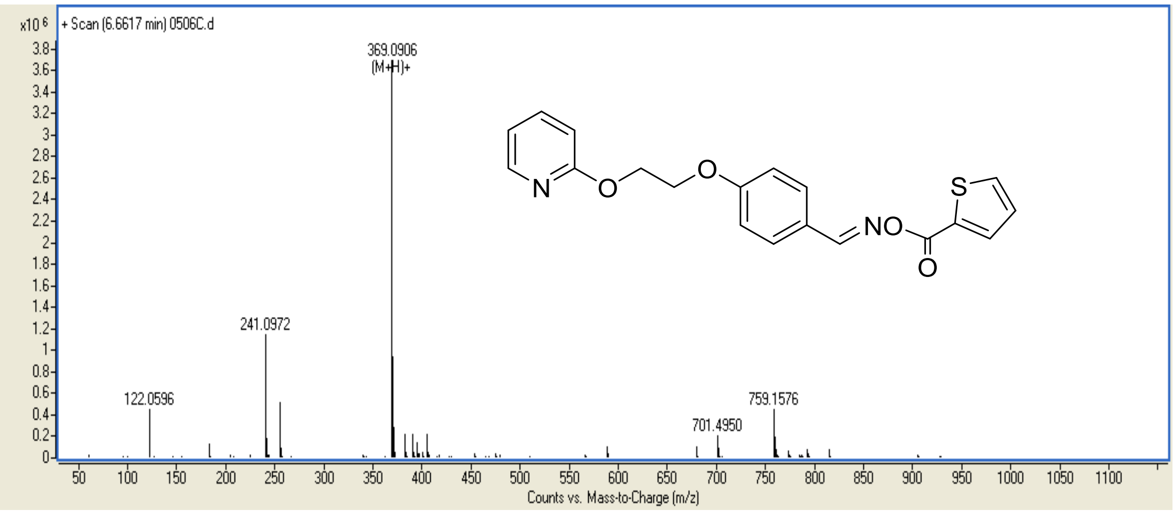

^{1}H-NMR spectrum of compound **5r.**

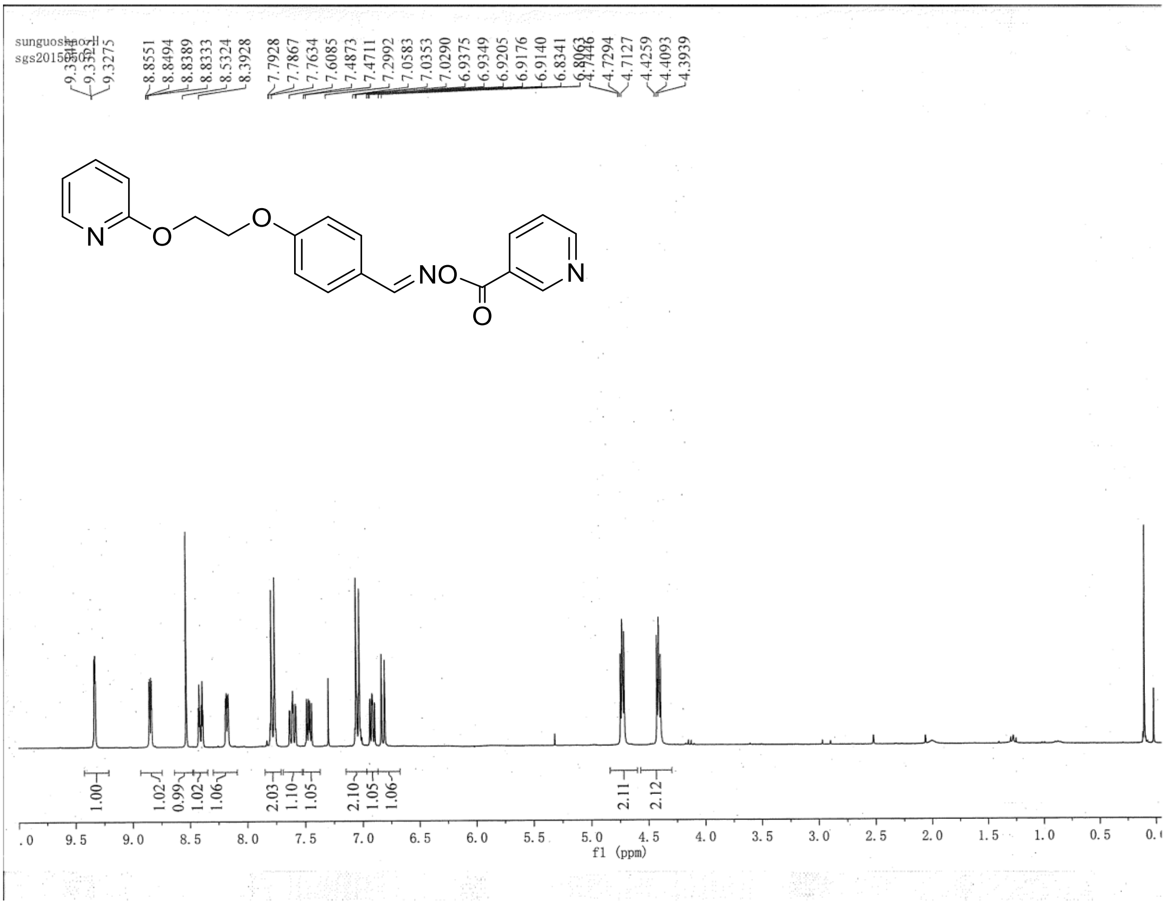

^{13}C-NMR spectrum of compound **5r.**

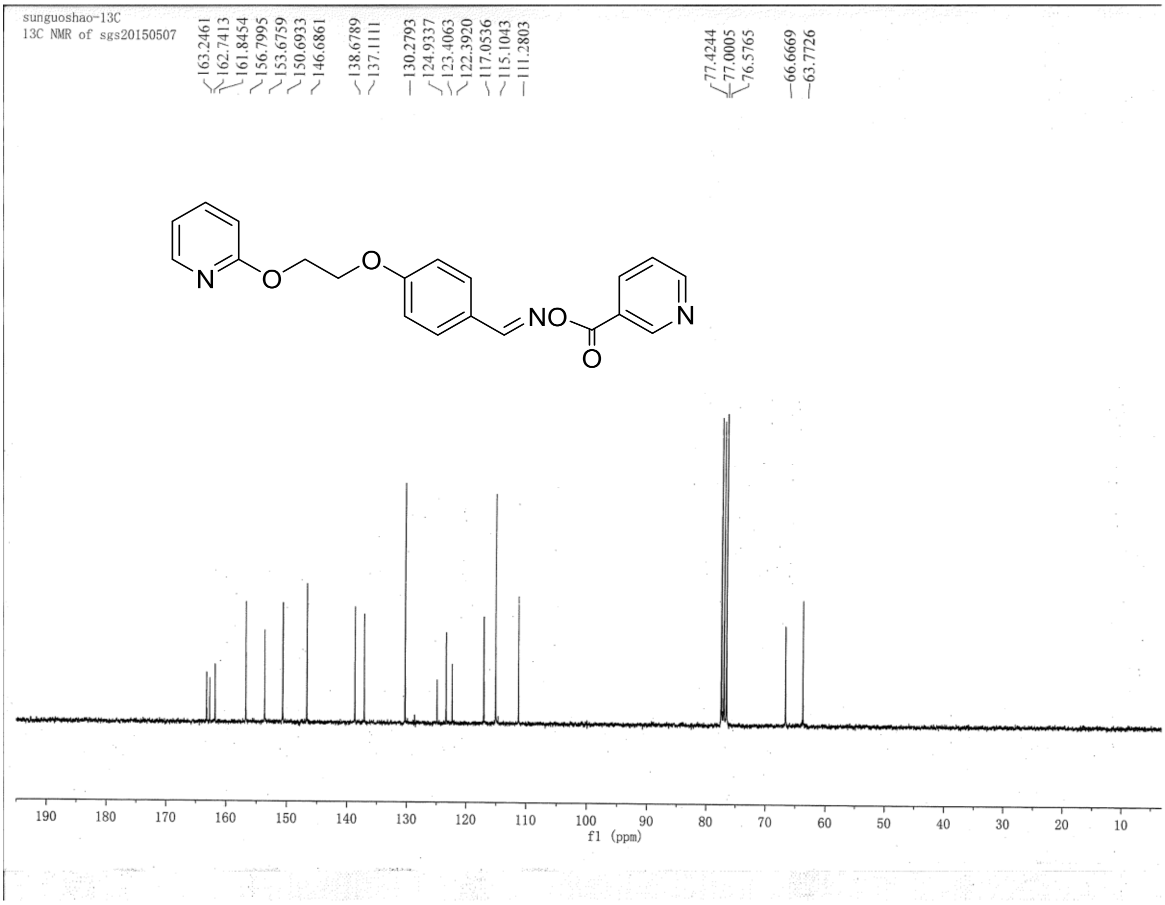

HRMS of compound **5r.**

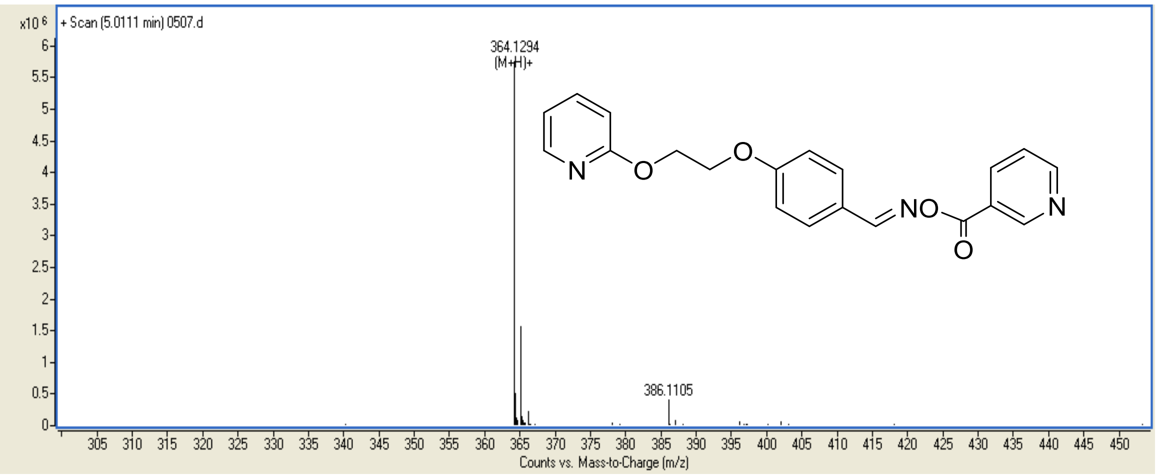

^{1}H-NMR spectrum of compound **5s.**

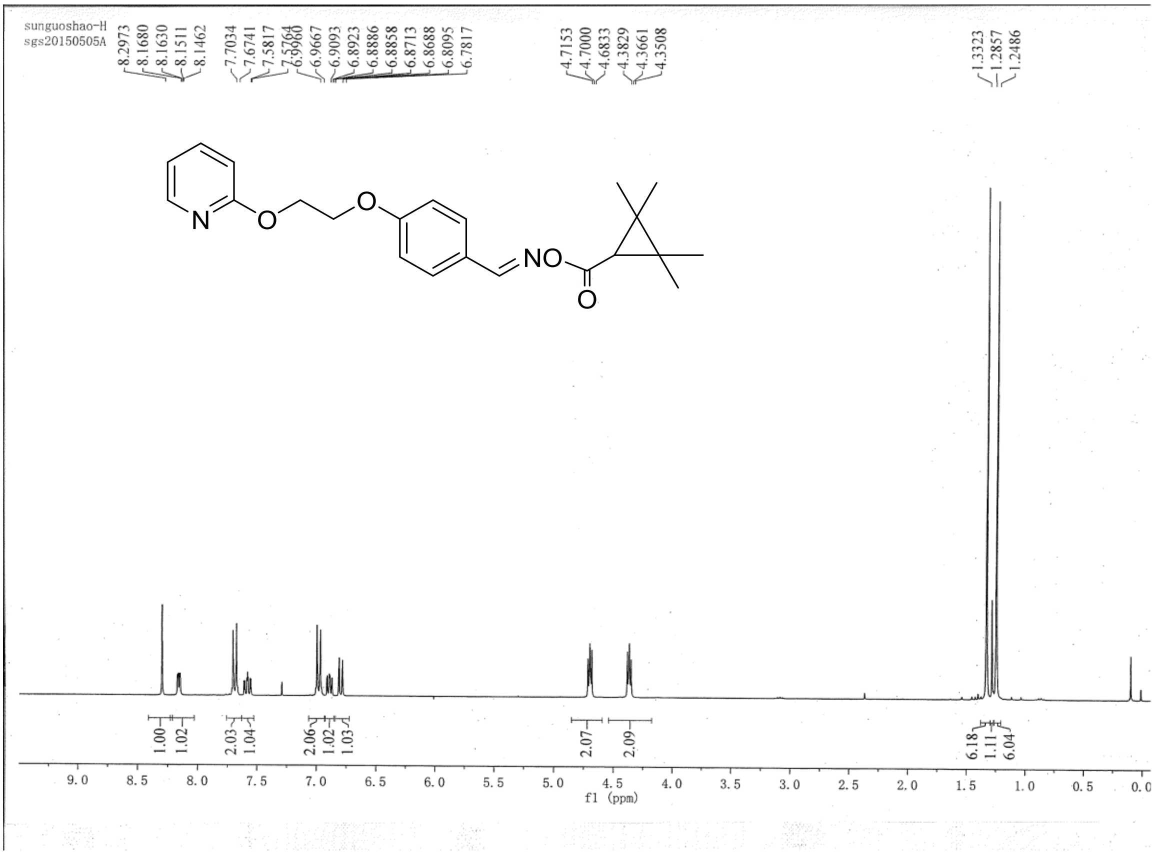

^{13}C-NMR spectrum of compound **5s.**

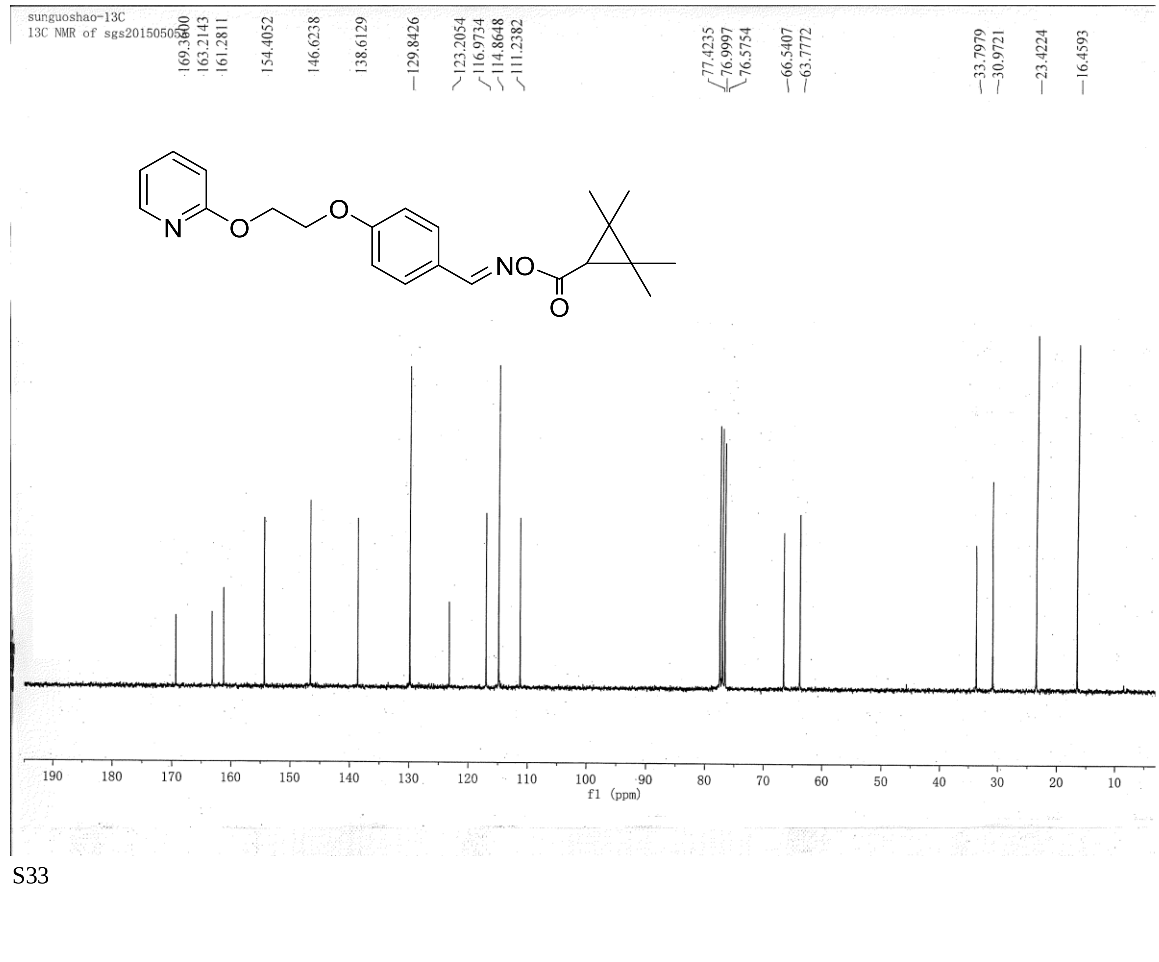

HRMS of compound **5s.**

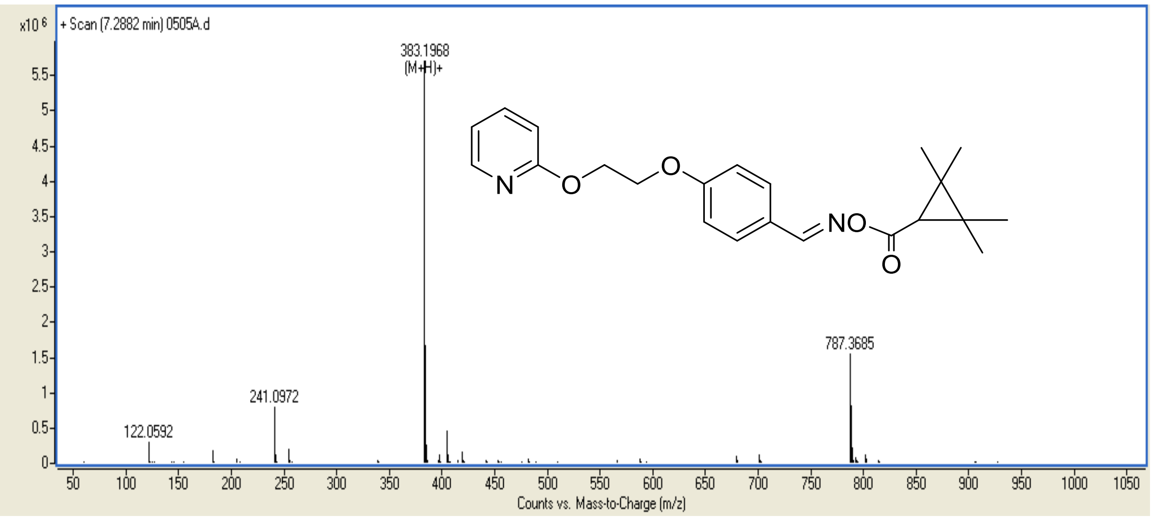

Supplement: Supplementary file 1 [file molecules-22-00958-s001.pdf]
